# Widespread, focal copy number variations (CNV) and whole chromosome aneuploidies in *Trypanosoma cruzi *strains revealed by array comparative genomic hybridization

**DOI:** 10.1186/1471-2164-12-139

**Published:** 2011-03-07

**Authors:** Todd A Minning, D Brent Weatherly, Stephane Flibotte, Rick L Tarleton

**Affiliations:** 1 Center for Tropical and Emerging Global Diseases, University of Georgia, Athens, Georgia, 30602, USA; 2 Department of Zoology, University of British Columbia, Vancouver, British Columbia V6T 1Z4, Canada

## Abstract

**Background:**

*Trypanosoma cruzi *is a protozoan parasite and the etiologic agent of Chagas disease, an important public health problem in Latin America. *T. cruzi *is diploid, almost exclusively asexual, and displays an extraordinarily diverse population structure both genetically and phenotypically. Yet, to date the genotypic diversity of *T. cruzi *and its relationship, if any, to biological diversity have not been studied at the whole genome level.

**Results:**

In this study, we used whole genome oligonucleotide tiling arrays to compare gene content in biologically disparate *T. cruzi *strains by comparative genomic hybridization (CGH). We observed that *T. cruzi *strains display widespread and focal copy number variations (CNV) and a substantially greater level of diversity than can be adequately defined by the current genetic typing methods. As expected, CNV were particularly frequent in gene family-rich regions containing mucins and trans-sialidases but were also evident in core genes. Gene groups that showed little variation in copy numbers among the strains tested included those encoding protein kinases and ribosomal proteins, suggesting these loci were less permissive to CNV. Moreover, frequent variation in chromosome copy numbers were observed, and chromosome-specific CNV signatures were shared by genetically divergent *T. cruzi *strains.

**Conclusions:**

The large number of CNV, over 4,000, reported here uphold at a whole genome level the long held paradigm of extraordinary genome plasticity among *T. cruzi *strains. Moreover, the fact that these heritable markers do not parse *T. cruzi *strains along the same lines as traditional typing methods is strongly suggestive of genetic exchange playing a major role in *T. cruzi *population structure and biology.

## Background

Human infection with *T. *cruz*i*, a vector-borne protozoan parasite, is the cause of Chagas disease, which is a potentially fatal malady endemic to much of Latin America. *T. cruzi *is a member of the order Trypanosomatida, and, like other members of this early diverged group of eukaryotes, is diploid and has a primarily clonal population structure which is extremely diverse [1]. The broad host range of *T. cruzi*, which includes over 100 species of both wild and domestic mammals (reviewed in [2]) may also contribute to its remarkable phenotypic and genetic diversity.

The population structure of *T. cruzi *has been examined by sub-genomic methods, such as multi-locus sequence typing (MLST) and microsatellite analyses, resulting in the classification of *T. cruzi *strains into six discrete typing units (DTU), type I and types IIa-e (reviewed in [3]). A change in nomenclature from these DTU names to TCI-TCVI has recently been suggested [4]. However, in the interest of clarity we will heretofore use the more familiar nomenclature of type I and types IIa-IIe. Although an asexual replication mechanism best explains the population structure of *T. cruzi *as it is currently understood, molecular analyses support the occurrence of at least two hybridization events in the past resulting in mosaic genomes in two of the DTU (types IIa and IIc) and hybrid genomes in another two (types IId and IIe), one of which (CL-Brener-type IIe) was used as the type strain for the *T. cruzi *genome sequencing effort [5].

The genotypic diversity evidenced by sub-genomic methods of analysis suggests that whole genome analyses of *T. cruzi *strain diversity would be fruitful, especially for elucidating the underpinnings of strain diversity in biological characteristics, such as variability in complements of large gene family members. Nearly one half of the *T. cruzi *genome contains repeat sequences largely comprised of thousands of members of large gene families, including trans-sialidases, mucin associated surface proteins (MASP), mucins, retrotransposon hotspot (RHS) proteins, dispersed gene family 1 proteins (DGF), and surface protease gp63 [6]. Many of these have been shown to be important targets of immune responses in infected hosts [7,8]. Isolates of *T. cruzi *(generally referred to as "strains") appear to show a near limitless range of variation in important biological characteristics, among these, the numbers of parasites in the blood and tissues of various hosts, the focus and location of inflammation and thus the morbidity and mortality in these hosts, and susceptibility of these isolates to anti-*T. cruzi *drugs. The current genetic classification cannot fully account for this variation (e.g. all type I strains are not equally virulent) despite the fact that a substantial proportion of this variation is almost certainly based upon genetic differences among isolates.

To date only the reference strain, CL-Brener, has been fully sequenced [6]. Moreover, the genome sequence of the CL-Brener strain has only recently been assembled into chromosome-sized pieces that will facilitate genome-wide strain comparisons [9]. To further explore the degree of genetic variability between *T. cruzi *isolates, and to examine the relationship between genotypic and biological diversity on a genomic scale, we used whole genome oligonucleotide tiling arrays to determine copy number variations (CNV) in 16 *T. cruzi *strains by competitive hybridizations using the CL-Brener strain as reference. Our results partially support the proposed type I - type II dichotomy in the *T. cruzi *population structure but also reveal similarities and differences between *T. cruzi *strains that cannot be explained by the current DTU scheme. These findings suggest that either coevolution of distinct, chromosome-specific CNVs frequently occurs in different *T. cruzi *strains or that chromosome exchange between *T. cruzi *strains is much more common than currently thought. The results also suggest that *T. cruzi *is remarkably permissive to substantial CNV, including whole chromosome CNV.

## Results

Whole genome oligonucleotide tiling arrays (~290,000 spots) were designed as described in the Methods section, using the only fully sequenced *T. cruzi *strain, the hybrid CL Brener, as a template. Because of the hybrid nature of the CL Brener strain, with its "Esmeraldo-like" (Esm) and "non-Esmeraldo-like" (non-Esm) alleles, it was possible to design allele-specific probes for many regions of the genome. Regions of the genome that are rich in genes that are members of large gene families have relatively few probes because the sequence similarities among family members did not permit the design of gene-specific probes.

As a validation of the accuracy of the arrays we performed hybridizations comparing wt *T. cruzi *strains with gene knockout parasite lines generated in our laboratory [10]. Additional file 1 shows the CGH data for one of these hybridizations comparing a knockout strain for enoyl-CoA hydratase/isomerase family protein (tandem genes each singly replaced - [Tc00.1047053511529.160 and Tc00.1047053511529.150]) vs wt *T. cruzi*. The CNV resulting from single knockout of one copy each of these two tandemly arrayed genes is obvious at the whole chromosome view of chromosome 35, panel A. Moreover, the close-up view in panel B reveals overlap of the Esm and non-Esm probes (green and blue spots, respectively) within the coding portions of the genes, but divergence of Esm and non-Esm ratios for the intergenic region. This reflects the fact that the Esm and non-Esm alleles for these two genes are nearly identical, but the Esm and non-Esm sequences for the intergenic region are not.

**Figure 1:**
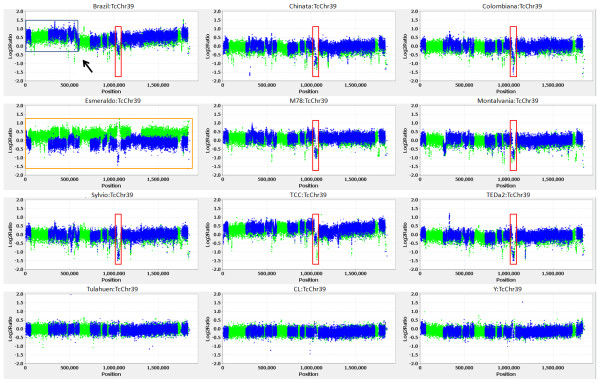
**Representative plots from 12 hybridizations for *T. cruzi *chromosome 39**. Each dot represents an oligonucleotide probe. The CL-Brener strain, which was used as the reference strain for genome sequencing, is hybrid, thus probes were designed to non-Esmeraldo (non-Esm) sequences (blue spots), Esmeraldo-like (Esm) sequences (green spots), non-Esm gene family sequences (black spots), and Esm gene family sequences (gray spots). In each panel positive log2 ratios of signal intensities (test strain/reference strain) represent amplification in the test strain and negative log2 ratios represent deletion in the test strain, relative to CL-Brener, which was the reference strain in all hybridizations. Shown are examples of segmental aneuploidies of different sizes boxed in gray (>500 kbp amplification in Brazil strain relative to CL-Brener -arrow) and red (~40 kbp deletion in several strains). The Esmeraldo hybridization (boxed in orange) shows decreased log2ratios for the non-Esm probes (blue) and increased log2ratios for the Esm probes (green), as expected because Esmeraldo is homozygous for the Esm haplotype.

Genomic DNA samples from 16 *T. cruzi *strains were compared to genomic DNA from the CL-Brener strain by competitive hybridizations on this array and the complete set of figures for all 41 chromosomes for each of the 16 strain comparisons are shown in Additional file 2 and the full dataset can be viewed in Additional files 3 and 4. The data have also been deposited in the Gene Expression Omnibus (http://www.ncbi.nlm.nih.gov/geo/) under the accession GSE23576. The most striking feature of these results is the very large number of both segmental (ranging in size from 500 bp to over 500 kbp) and whole chromosome aneuploidies, representative examples of which are shown in the hybridization results for chromosome 39 for 12 strains (Figure 1). Probes with log2ratios approximating zero are present in equal copy numbers in both the test and reference (CL Brener) strains and probes with positive log2ratios have higher copy number in the test strains versus the reference strain, and thus represent amplifications in the test strain. Probes with negative log2ratios indicate deletions in the test strain and/or sufficient sequence divergence to result in decreased hybridization in the test channel. Segmental aneuploidies (e.g. a 500 kbp segment in the Brazil strain [arrow]) and shared, focal deletions in multiple strains are readily evident (Figure 1 and Additional file 2). Sequence divergence resulting in decreased hybridization intensity can be seen in the Esmeraldo panel where the green dots (representing Esmeraldo allele-specific probes) have positive ratios and blue dots (non-Esmeraldo probes) have negative log2ratios (Figure 1). However, note that the average log2ratio for all probes is near zero in the Esmeraldo panel, suggesting that Esmeraldo is homozygous for the Esmeraldo haplotype as would be expected and that the total number copies of chromosome 39 in this strain is 2 (assuming, of course, that CL-Brener has 2 copies of chromosome 39). The presence of apparent deletions in the test strains that are shared between many genetically divergent test strains (as seen in the red boxed regions) suggests that such instances are *bona fide *deletions rather than sequence divergence.

**Figure 2:**
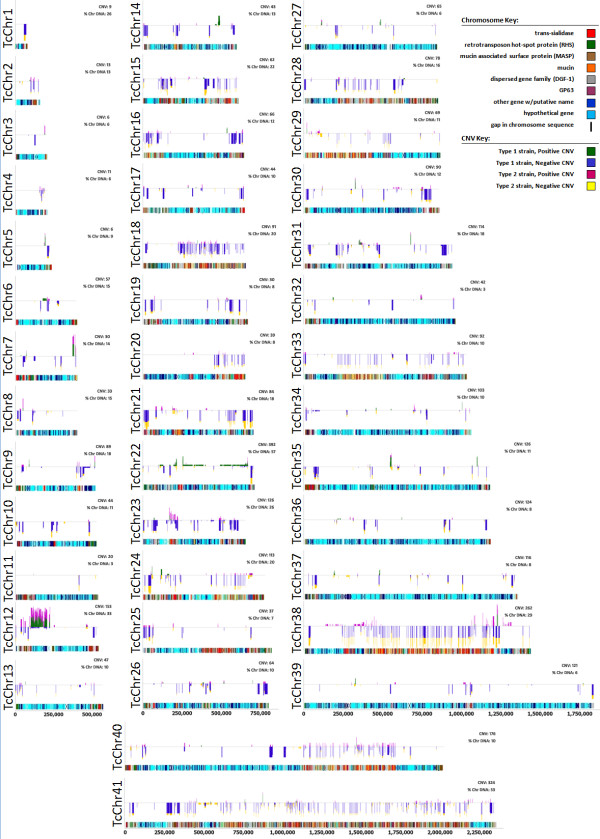
**Graphical view of proportion of strains displaying CNV for each sequence feature on each chromosome**. A sequence feature is defined as either an annotated gene or the un-translated region (UTR) between two genes. CNV criteria were as follows: minimum log2 ratio of signal intensities (test strain/reference) +/- 0.6, minimum number of probes 5, tolerance for extending a CNV 0.1. At the top of each panel is a line drawing representing a chromosome. Vertical bars represent CNV that met the criteria for significance. Vertical bars above the line are amplifications and those below the line are deletions relative to CL-Brener. The height (depth) of the vertical bars is proportional to the number of strains showing that CNV. The vertical bars are colored to indicate strain type (type 1 and type 2) as follows: green, type 1 strains amplification; blue, type 1 strains deletion; maroon, type 2 strains amplification; yellow, type 2 strains deletion. Below each chromosome line drawing is a diagram representing the annotated chromosome. Genes are color coded based on annotation; named genes not belonging to large gene families (dark blue), hypothetical genes (light blue), trans-sialidases (red), mucin associated surface proteins (MASP) (burnt orange), mucins (orange), retrotransposon hotspot (RHS) proteins (green), dispersed gene family 1 (DGF) (gray), surface protease gp63 (purple).

To identify the regions of the *T. cruzi *genome that are most prone to CNV, we annotated segments of the genome that had CNVs relative to the overall average of all strains (Figure 2). 'Hotspots' of CNV were readily evident and were present on every chromosome, although much more prevalent on some (e.g. 18, 38 and 41) than others (e.g. 11, 34, 36, 37, 39). The CNV were also focal and widespread, frequently associated with gene family rich regions of the genome (note that chromosomes 18, 38, and 41 are the most gene family rich chromosomes), but also in core regions. Due to the scarcity of probes within the gene family rich regions a statistical test of the significance of the bias of hotspot regions being located in gene family rich regions of the genome was not possible.

The classes of genes mapping to these hotspot regions are ranked in order of frequency of CNV in Table 1 (for all gene groups comprised of 5 or more genes, each with a minimum of 5 probes per 500bp). Perhaps not surprisingly, genes that are members of large families of surface proteins, such as the mucins, trans-sialidases, mucin-associated surface proteins (MASPs), and surface proteases are among those with the highest number of CNV among the strains tested, with type II mucins showing CNV over 70% of the time. Interestingly, protein kinases, a large and diverse family of related genes, as well as hypothetical proteins were the gene groups with the least association with hotspot regions, suggesting that all or nearly all of them may be under strict copy number control. Other genes that are relatively stable in terms of copy number included genes for ribosomal proteins and DNA repair, consistent with their expected essential roles. CNVs in gene groups represented by less than 5 genes are shown in Additional file 5.

**Table 1:** Distribution of genes associated with hotspot regions, minimum of 5 candidates on the arrays

Gene Name	# Genome Occurrences^1^	#Array Occurrences^2^	# Array Candidates^3^	# Sig CNV^4^	% Sig of Candidates
mucin TcMUCII	728	506	164	121	73.7
serine carboxypeptidase	7	7	6	4	66.6
tryptophanyl-tRNA synthetase	23	8	6	4	66.6
mucin-associated surface	1377	948	437	243	55.6
retrotransposon hot	753	419	82	41	50
ATP-dependent chaperone	5	5	5	2	40
beta galactofuranosyl	66	47	10	4	40
mitogen-activated protein kinase	11	11	10	4	40
Sialidase	3209	789	135	51	37.7
clathrin coat	7	7	7	2	28.5
surface protease	425	258	88	25	28.4
myosin heavy	19	19	17	3	17.6
PIF1 helicase-like	12	12	12	2	16.6
mismatch repair	13	13	13	2	15.3
ATP-dependent DEAD/H	106	88	66	10	15.1
leucine-rich repeat	17	16	16	2	12.5
DNA topoisomerase	9	9	9	1	11.1
protein tyrosine	10	10	9	1	11.1
cytochrome c	20	18	13	1	7.6
protein transport	16	16	14	1	7.1
elongation factor	203	65	15	1	6.6
ABC transporter	33	33	33	2	6
ubiquitin-conjugating enzyme	24	24	23	1	4.3
serine/threonine protein	80	78	78	3	3.8
60S ribosomal	106	106	93	3	3.2
DNA repair	37	36	35	1	2.8
RNA-binding protein	80	72	71	2	2.8
hypothetical protein	11812	10489	9735	122	1.2
protein kinase	311	277	270	1	0.3

1. # genome occurrences = number of instances for the annotation in the genome.

2. # array occurrences = number of annotated genes with probes on the arrays.

3. # array candidates = number of annotated genes represented by probes on the arrays and which had probe density of 5 unique probes per 500 bp. Genes could have insufficient probe density due to repeat regions or they could have too few probes due to length of the gene (minimum of 5 probes).

4. # sig CNV = CNV with a minimum log2 ratio difference of +/- 0.5, for a minimum of 5 probes over a segment size of 500 bp in at least 5 test strains.

**Figure 3:**
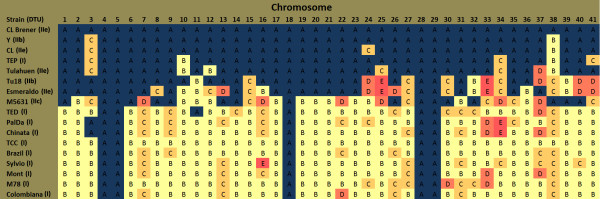
**Chromosomes were typed based on CNV signatures**. Diagnostic CNV were selected based on the criteria that they were at least 500 bp in length, represented by at least 5 oligonucleotide probes, and had log2ratios of at least +/- 1 (tolerance of 0.2) in at least 5 strains. Up to two CNV meeting the criteria were selected for each chromosome based on their ability to differentiate between the strains; i.e. the CNV that separated the strains into the highest number of groups compared to other candidate diagnostic CNV were selected. Thus, for each chromosome there were a finite number of signature types to which strains were assigned based upon the presence/absence of the diagnostic CNV's the direction of change (log2ratio +/- 0.8). Each chromosome type is represented by a different color, with CL-Brener always assigned to type 'A' (blue). The strains were then sorted based on the overall similarity of their chromosome types, taken together, relative to CL-Brener.

The typing of *T. cruzi *into 6 discrete typing units (DTUs) is based upon a relatively small number of loci, including small subunit rRNA, elongation factor 1α, actin, dihydrofolate reductase-thymidylate sythase, and trypanothione reductase among others [11,12]. To determine if this DTU classification system matched to patterns of CNV among typed strains, we 'typed' chromosomes based on their CNV patterns, creating CNV signature types (Additional file 4) and then grouped these CNV-typed *T. cruzi *strains by similarity in the number of shared chromosome types (Figure 3). The typing results suggest that there is substantially more genetic diversity among the type I strains than there is among the type II strains. This is consistent with recent microsatellite analyses of type I strain diversity [13]. However, the apparently greater diversity among type I versus type II strains observed in our study could be a result of the feature selection. Features for typing were selected by an unbiased computational method, but not all of the nearly limitless possible combinations of available CNV features were used in the typing. Although type I and type II strains grouped together in the classification, on an individual chromosome basis some type I strains clearly had chromosomal CNV patterns matching those in type II strains and vice versa. For example, on chromosome 11 Esmeraldo, M5631, and Tu18 (types IIb, IIc, and IIb respectively) each have a CNV that is shared among all of the type I strains (Additional file 4, slide 11). Yet the other type II strains, including Y strain which is also type IIb, do not have this CNV. This CNV, a deletion, corresponds to the locus for proline racemase that may be a target of immune selection [14]. Taken together, these data suggest that either there is extensive homoplasy among *T. cruzi *strains (i.e. the same amplification/deletion events occurring multiple times in strains that are evolving independently) or that chromosome exchange has occurred more frequently in *T. cruzi *than the current two-hybridization event theory would suggest [5].

## Discussion

In this study, we used whole-genome tiling arrays to catalog the diversity in gene copy number among 17 *T. cruzi *strains with widely varying biological characteristics. The overwhelming conclusion from this work is that there is substantial and widespread variation in gene copy numbers among *T. cruzi *isolates. The observation of CNVs in *T. cruzi *is not surprising. The relative infrequency of sexual recombination, coupled with the near absence of transcriptional promoters in *T. cruzi *means that both the generation of genetic diversity and the regulation of the level of proteins is determined in part by within-isolate gene recombination and amplification. Nevertheless, the extent of variation in a relatively small set of isolates is quite dramatic. This variation is evident from the level of entire chromosomes down to individual genes, and CNVs tend to cluster in regions that are heavily populated with repeat sequences, including those encoding the members of gene families of the surface proteins, trans-sialidases, mucins, mucin associated surface proteins (MASP's), as well as retrotransposon hot spot (RHS) proteins, the dispersed gene family 1 (DGF-1), and protease gp63. Multicopy genes are frequently sources of mitotic recombination resulting in amplification and deletion of duplicate genes [15] so their relationship to regions of CNV is also not unexpected. The surface protein families in particular are known to be the major targets of protective immune responses and this selective pressure presumably drives their expansion and variation among strains.

In addition to the expected association of CNV with genes that are under immunological pressure, the presence and absence of CNV among other gene groups may be informative as to their functions and unique activities in *T. cruzi*. A relatively high fraction of serine carboxypeptidases, tryptophanyl aminoacyl tRNA synthetases, beta galactofuranosyl transferases and mitogen-activated protein kinases show CNVs among the *T. cruzi *isolates studied. The tryptophanyl aminoacyl tRNA synthetases are expanded to 10 distinct genes (not including pseudogenes) in *T. cruzi *whereas *T. brucei *and *Leishmania *each have only two, a cytoplasmic and a mitochondrial version. Interestingly, aminoacyl-tRNA synthetase genes in other eukaryotes have been reported to be increased in number and to acquire a diverse set non-enzymatic functions, including as inflammatory and angiogenic cytokines [16]. The location of this expanded set of the tryptophanyl tRNA sythetases genes in hotspots for recombination in *T. cruzi *also suggests that they may be modified and selected for as yet undetermined secondary functions. Similarly, *T. cruzi *has 37 beta-galactofuranosyl transferase genes.(compared to 2 for *L. braziliensis*, 1 each for *L. major *and *L. infantum*, and none for *T. brucei*) located in regions of high between-strain CNV. These enzymes are involved in the synthesis of glycans on mucins in *T. cruzi*. Mucin glycan structure is complex and heterogeneous between *T. cruzi *strains, again consistent with the expansion and variation in transferases among *T. cruzi *strains noted herein [17]. Conversely, only 1 of 270 protein kinases that passed the data filters for this analysis and <2% of the nearly 10,000 hypothetical proteins were associated with hotspot regions, suggesting that variation, amplification and recombination of these genes is not tolerated or provides no selective advantage.

Although this analysis suggests extensive occurrence of whole chromosome aneuploidy in *T. cruzi*, the range of chromosome numbers as well as the absolute number of individual chromosomes in each strain cannot be estimated based upon our analysis. The decreased signal intensities evident across whole chromosomes in this CGH analysis would appear to be deletions but could also be in part due to sequence divergence in the test strains relative to the CL Brener reference sequences. Since individual isolates or strains of *T. cruzi *are thought to evolve without significant between-strain genetic exchange due to the relative absence of sexual recombination [1], the accumulation of sequence variants would be expected. Normally, traditional cytological methods such as metaphase spreads would be used to resolve karyotypic differences between isolates. However, this approach is not possible in the case of *T. cruzi *because *T. cruzi *replicates via endodyogeny, where the nuclear membrane does not break down and chromosomes do not fully condense during replication [18]. Nevertheless it seems highly unlikely that sequence divergence alone could explain our observations of chromosomes with significantly decreased signal intensities in many of the test strains relative to the CL-Brener reference. And certainly whole chromosome *increases *in signal relative to the reference strain cannot be accounted for by sequence divergence, since the target probes are based upon the reference strain sequence and diverged sequences are unlikely to out-compete the homologous sequences. For example, Y, Colombiana, M5631, wtCL, and TeDa2 each appear to have an extra chromosome 3 (Additional file 4, slide 3). It is most reasonable to interpret significant deviation from a log2 ratio of zero for test strain vs. reference strain over the length of a chromosome as being indicative of a real difference in the number of copies of that chromosome in the test and reference strains, supporting the conclusion that *T. cruzi *karyotypes are highly variable between strains and suggesting that there is perhaps no 'euploid' state for *T. cruzi*. This karyotypic plasticity may be another mechanism *T. cruzi *uses to generate diversity in spite of apparently being asexual or nearly so and may to some extent parallel what has been observed for the pathogenic yeast, *Candida glabrata*. Array CGH and pulsed field gel electrophoresis analyses of this haploid, asexual organism revealed extensive variability in the karyotypes of *C. glabrata *strains and suggest that this variability may be linked to drug resistance [19,20].

The biological characteristics of *T. cruzi *strains have been used to classify isolates into biodemes [21,22] and as well the DTU classification has been associated to certain transmission and virulence characteristics of isolates [23-26]. Nevertheless, the link between DTU type and the biological characteristics of a strain are not strong [27]. Likewise, we find that the DTU organization of strains is a poor predictor of patterns of CNV in individual chromosomes. Thus, clear and specific patterns of amplifications and deletions in chromosomes are observed among strains, but these specific patterns are almost never restricted to or predictive of DTU type. This result has two important implications. First, the substantial and even continuous variation in biological characteristics of *T. cruzi *isolates will be difficult to account for using a limited number of genetic markers. Patterns of CNV provides another tool, but this too is not sufficient to predict behaviors that likely have a complex genetic basis. Nevertheless, these typing approaches do provide insights into the population structure of the species and the evolution of individual "strains" that are thought to be genetically isolated from each other. The results of typing of chromosomes by CNV patterns that we present herein provides new insights but also new questions related to genetic exchange in *T. cruzi*. The observation of shared patterns of CNV that do not track with the DTU type of a strain and are not consistent between chromosomes within the same strains can be explained in two ways: either such similar patterns of CNV are arising independently in isolates, perhaps due to common selective forces, or chromosomes of *T. cruzi *are being resorted or exchanged between isolates at a much higher frequency than is currently appreciated. The similarity and complexity of CNV would seem to favor the latter of these possibilities but a mechanism by which such exchange would occur is not clear. The full sequencing of additional *T. cruzi *isolates, as is currently underway, should help discriminate between these possibilities. Moreover, CGH arrays designed from the sequences of multiple *T. cruzi *strains, analogous to multi-species taxonomic arrays for *Saccharomyces cerevisiae *may help resolve the extent and nature of genetic exchange between *T. cruzi *strains and reveal heretofore undiscovered instances of introgression [28].

In addition to these insights into the biology and evolution of *T. cruzi*, the identification of "hot spots" for CNV within the *T. cruzi *genome should also provide guidance in the selection of candidates for vaccines and for targets for drug development. Although selecting candidates that are encoded outside these hot spots does not guarantee the absence of variation or the development of variation between isolates, avoiding genes that are in such hotspots would seem prudent.

## Conclusions

CNV among *T. cruzi *strains are substantial in number, widespread throughout the *T. cruzi *genome, range in size from a few hundred base pairs to whole chromosomes (based upon the assembled CL-Brener genome), and are discordant with traditional DTU assignments for the strains tested. Taken together, these results suggest that there is much more genotypic diversity among *T. cruzi *strains than can be fathomed using traditional typing methods and that genetic exchange, possibly by the proposed "fusion then loss" mechanism [29], occurs between *T. cruzi *strains much more frequently than current dogma supposes. The large number of CNV shared between divergent DTU supports the genetic exchange hypothesis over the hypothesis that the shared CNV arose due to homoplasy.

CNV in *T. cruzi *tended to be concentrated in gene family rich regions of the genome, suggesting that the highly repetitive nature of the sequences in these regions is a strong driver of mitotic recombination in *T. cruzi *whether occurring within diploid strains or in strains resulting from hybridization events.

Neither the traditional DTU assignments for the *T. cruzi *strains used in this study nor the strain groupings based upon CNV patterns are currently good predictors of the biological characteristics of these strains. Thus, further CGH and sequencing studies of a larger panel of well-characterized *T. cruzi *strains is warranted to determine appropriate genotypic markers of *T. cruzi *biological diversity. Moreover, it would seem prudent to use the CNV data reported herein when selecting targets for drug and vaccine studies in order to avoid genes in hotspots of CNV occurrence.

## Methods

### Parasites and DNA isolation

*T. cruzi *strains were selected to represent a biologically diverse sample set from multiple DTU (Table 2). Epimastigotes were cultured in Liver Infusion Tryptose (LIT) medium and harvested as previously described [30]. Genomic DNA was obtained as previously described [31]. Briefly, epimastigotes were washed three times with ice-cold PBS, resuspended at a final density of 2E + 08 cells per ml in lysis buffer (150 mM NaCl, 100 mM EDTA, 100 ug/ml proteinase K, 10 ug/ml RNase A, and 0.5% sodium sarcosinate, pH 8.0) and incubated at 50°C for 30 m. Lysates were then extracted twice with phenol/chloroform and the DNA was ethanol precipitated, then resuspended in 5 mM Tris.Cl, pH 7.5. DNA sample integrity was checked by agarose gel electrophoresis. Genomic DNA for CL-Brener strain and Esmeraldo strains were kindly provided by JM Kelly and B Zingales, respectively.

**Table 2:** *Trypanosoma cruzi *strains used in this study

strain	origin	host	DTU	virulence	drug resistance	reference
Brazil	Brazil	Human	I	high	low	[37]
Chinata	Bolivia	*Triatoma infestans*	I	high	ND	unpublished
Colombiana	Colombia	Human	I	high	moderate	[21]
Esmeraldo	Brazil	Human	IIb	moderate	ND	[38]
M5631	Brazil	*Dasypus novemcinctus*	IIc	ND	ND	[39]
M78	Argentina	Human	I	low	ND	[40]
Montalvania	Brazil	Human	I	high	moderate	Andrade unpublished
PalDa1 (clone 9)	Argentina	*Didelphis albiventris*	I	low	ND	[41]
Sylvio X10/4	Brazil	Human	I	low	ND	[42]
TCC	unknown	unknown	I	low	ND	[43]
TEDa2 (clone 4)	Argentina	*Didelphis albiventris*	I	ND	ND	[41]
TEP6 (clone 5)	Argentina	dog	I	ND	ND	[41]
Tu18 (clone 1)	Bolivia	*Triatoma infestans*	IIb	ND	ND	[44]
Tulahuen	Chile	Human	IIe	high	low	[45]
wtCL	Brazil	*Triatoma infestans*	IIe	high	low	[46]
Y	Brazil	Human	IIb	high	low	[47]
CL-Brener	Brazil	*Triatoma infestans*	IIe	high	ND	[48]

### Array Design

Whole genome tiling arrays for comparative genome analysis were designed as previously described [32] with the notable exception that all the criteria related to sequence similarity with other regions of the genome had to be relaxed in *T. cruzi *in order to ensure proper coverage. We used 50-mer oligonucleotide probes and the microarray had a total capacity of approximately 380,000 probes, approximately 25% of which were used for preliminary quality assessment in this first generation design. The ~290,000 probes relevant for the current work were selected with the following procedure: 1) only the contigs including annotated genes were targeted, 2) the 50-mers occurring more than 4 times in the genome were eliminated, 3) the homopolymers longer than 5 nucleotides were eliminated, 4) only the 50-mer oligonucleotides with a melting temperature Tm within +- 5°C of the median melting temperature were kept (where Tm = 0.41GC + constant), 5) the oligonucleotides requiring more than 150 cycles [33] to synthesize were eliminated, 6) the oligonucleotides with a self-folding energy smaller than -1 kcal/mol according to a hybrid-ss-min calculation [34] were eliminated, and 7) from the probes passing all the above filters ~290,000 were selected with a strategy designed to maximize the uniformity of coverage of the entire genome. However, for some regions of the genome, gene family rich regions in particular, the DNA sequences were highly repetitive resulting in low tiling densities for those regions. For the same reason probes were not designed to sequencing scaffolds that were not used in the genome assembly. Construction of the arrays was performed by Roche NimbleGen Inc. (see reference [35] for additional details).

### Hybridization, Scanning, and Image Analysis

Genomic DNA from *T. cruzi *strains was sonicated and labeled per the Nimblegen protocol. Labeling was by random-primed synthesis using Cy3/5-labeled random nonamers, and mixed probes were hybridized to the arrays per the manufacturer's protocol. Microarrays were scanned using a ScanArray 5000 (Perkin Elmer) and signal intensity files were generated using NimbleScan software. Ratios of fluorescence intensities were calculated without applying any background subtraction and the log2ratio values were normalized following a LOESS regression and visualized using the R statistical software [36]. The data from these experiments have been deposited in the Gene Expression Omnibus (http://www.ncbi.nlm.nih.gov/geo/) under the accession GSE23576.

## Authors' contributions

TAM designed and performed the microarray experiments, analyzed and interpreted the data, and wrote the manuscript. DBW assisted in the bioinformatics and data analysis and wrote the CGH_Viewer program. SF designed the microarrays, performed validation experiments, and wrote the within-hybridization normalization program. RLT initiated and guided the project, analyzed and interpreted data, and wrote the manuscript. All authors have read and approved the final manuscript.

## Supplementary Material

Additional file 1 **Microsoft PowerPoint file of theCGHViewer view of *T. cruzi *chromosome 35 showing the CNV generated by knockout of one copy each of ECH1 and ECH2 (enoyl-CoA hydratase/isomerase family protein; Tc00.1047053511529.160, Tc00.1047053511529.150)**. Each dot represents an oligonucleotide probe. The CL-Brener strain, which was used as the reference strain for genome sequencing, is hybrid, thus probes were designed to non-Esmeraldo (non-Esm) sequences (blue), Esmeraldo-like (Esm) sequences (green), non-Esm gene family sequences (black), and Esm gene family sequences (gray). Positive log2 ratios of signal intensities (wild type strain/knockout strain) represent deletion in the knockout strain and negative log2 ratios represent amplification in the knockout strain, relative to wt *T. cruzi*. Units for the X axis (Position) are base pairs. Inset in panel A is the GBrowse view of the locus (ECH genes purple circle). Panel B is a close-up view of the locus on chromosome 35. Click here for file

Additional File 2 **PowerPoint file of all of the array data ordered by chromosome**. Representative plots from 16 hybridizations for each *T. cruzi *chromosome. Each dot represents an oligonucleotide probe. The CL-Brener strain, which was used as the reference strain for genome sequencing, is hybrid, thus probes were designed to non-Esmeraldo (non-Esm) sequences (blue), Esmeraldo-like (Esm) sequences (green), non-Esm gene family sequences (black), and Esm gene family sequences (gray). In each panel positive log2 ratios of signal intensities (test strain/reference) represent amplification in the test strain and negative log2 ratios represent deletion in the test strain, relative to CL-Brener, which was the reference strain in all hybridizations. Boxed regions were the features selected for chromosome typing as explained in Figure 3. The different patterns observed for each chromosome are displayed at the bottom with letters corresponding to the typing letters in Figure 3. Blue boxes denote lower copy number in the test strain versus the reference strain, red boxes higher copy number in the test strain, and black boxes equal copy number in the test and reference strains. In each case for each chromosome, the CL-Brener type was the default type "A." Also, while two strains may have been assigned to the same CNV signature type for a given chromosome, they were not necessarily identical for that chromosome, as not every single CNV for every single chromosome was used in the typing (such an analysis would render every chromosome for every strain unique and make finding common patterns impossible). Note that chromosomes 4, 5, 18, 28, and 29 did not present sufficiently informative typing regions. Thus, all strains were type "A" for these chromosomes. Also, the CNV were haplotype specific. Therefore, in some cases this made the up or down calls (box color) appear incorrect, especially if the log2ratio for the feature was off the scale making it appear as if the boxed region is referring to the other haplotype. For example see PalDa for chromosome 8. In the boxed region the green (Esm) probes appear to be up yet the box is blue, indicating lower copy number, because the feature refers to non-Esm probes which are off the scale of the figure. Lastly, the boxes are guides to identifying the CNV used for typing, but they do not represent the exact bounds of those typing regions. In some cases due to the scale of the figure CNV that are close to the typing CNV appear to be part of the typing CNV. Click here for file

Additional file 3 **A java-based executable file for viewing all of the CGH array data from this study**. The CGH data were visualized and explored using 'CGH_Viewer,' which was written in the Java programming language. Additional file 2 is a Windows executable (.exe) that will install the CGH_Viewer, along with Java if it is not detected, on the target computer. The CGH_Viewer takes as input the mapping of probes from the microarray to the assembled chromosomes of *T. cruzi *as well as the results of 1 or more experiments between 2 strains. The probe-to-chromosome mapping file and the 16 experimental files are provided in the "Data" sub-folder. The results (dot plots of log2 ratios) of multiple chromosomes and multiple experiments may be viewed simultaneously. Zooming on an area of interest on a chromosome will show the same region across all experiments. The data can be filtered based on 1) the haplotype of sequence from which the probes were designed as well, 2) the ID or name of the sequence from which the probe was designed, 3) by raw intensity of the probe (a quality measurement), or 4) a sub-region of a chromosome. For additional information, see the provided documentation in the CGH_Viewer program folder (c:\Program Files\CGH_Viewer\). Click here for file

Additional file 4 **Excel file containing the average normalized log2 ratios of signal intensities (test strain/CL Brener) for each coding and non-coding region in the *T. cruzi *genome for each of the hybridizations performed in this study**. Esmeraldo-like (Esm) and non-Esmeraldo (non-Esm) probes for the indicated regions are averaged separately. Density is the genomic range (in base pairs) divided by the number of probes covering that range. Click here for file

Additional file 5 **MSWord file containing a chart of the distribution of genes associated with hotspot regions having less than 5 candidates on the arrays**. Candidates were determined by having at least 5 probes within a maximum sequence size of 500 bp. Click here for file
