# Supplementary material for: Widespread, focal copy number variations (CNV) and whole chromosome aneuploidies in Trypanosoma cruzi strains revealed by array comparative genomic hybridization
Source: BMC Genomics. 2011 Mar 7;12:139. doi: 10.1186/1471-2164-12-139 (PMC3060142; doi:10.1186/1471-2164-12-139)
Supplement: Additional file 1 — Microsoft PowerPoint file of theCGHViewer view of T. cruzi chromosome 35 showing the CNV generated by knockout of one copy each of ECH1 and ECH2 (enoyl-CoA hydratase/isomerase family protein; Tc00.1047053511529.160, Tc00.1047053511529.150). Each dot represents an oligonucleotide probe. The CL-Brener strain, which was used as the reference strain for genome sequencing, is hybrid, thus probes were designed to non-Esmeraldo (non-Esm) sequences (blue), Esmeraldo-like (Esm) sequences (green), non-Esm gene family sequences (black), and Esm gene family sequences (gray). Positive log2 ratios of signal intensities (wild type strain/knockout strain) represent deletion in the knockout strain and negative log2 ratios represent amplification in the knockout strain, relative to wt T. cruzi. Units for the X axis (Position) are base pairs. Inset in panel A is the GBrowse view of the locus (ECH genes purple circle). Panel B is a close-up view of the locus on chromosome 35. [file 1471-2164-12-139-S1.PPT]

## Slide 1
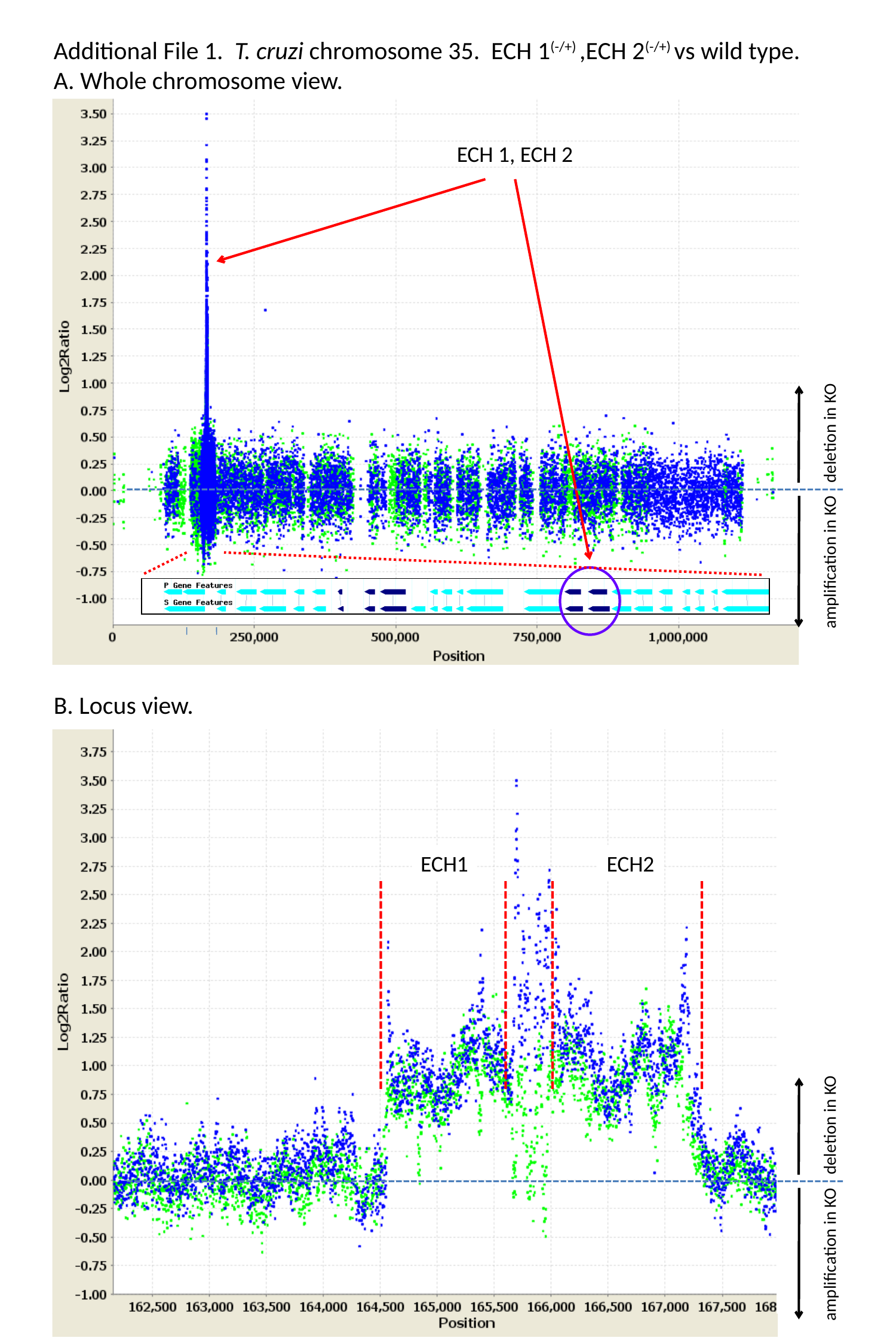

Additional File 1. T. cruzi chromosome 35. ECH 1(-/+) ,ECH 2(-/+) vs wild type.
A. Whole chromosome view.
ECH 1, ECH 2
deletion in KO
amplification in KO
B. Locus view.
ECH1
ECH2
deletion in KO
amplification in KO
