# Supplementary material for: Widespread, focal copy number variations (CNV) and whole chromosome aneuploidies in Trypanosoma cruzi strains revealed by array comparative genomic hybridization
Source: BMC Genomics. 2011 Mar 7;12:139. doi: 10.1186/1471-2164-12-139 (PMC3060142; doi:10.1186/1471-2164-12-139)
Supplement: Additional File 2 — PowerPoint file of all of the array data ordered by chromosome. Representative plots from 16 hybridizations for each T. cruzi chromosome. Each dot represents an oligonucleotide probe. The CL-Brener strain, which was used as the reference strain for genome sequencing, is hybrid, thus probes were designed to non-Esmeraldo (non-Esm) sequences (blue), Esmeraldo-like (Esm) sequences (green), non-Esm gene family sequences (black), and Esm gene family sequences (gray). In each panel positive log2 ratios of signal intensities (test strain/reference) represent amplification in the test strain and negative log2 ratios represent deletion in the test strain, relative to CL-Brener, which was the reference strain in all hybridizations. Boxed regions were the features selected for chromosome typing as explained in Figure 3. The different patterns observed for each chromosome are displayed at the bottom with letters corresponding to the typing letters in Figure 3. Blue boxes denote lower copy number in the test strain versus the reference strain, red boxes higher copy number in the test strain, and black boxes equal copy number in the test and reference strains. In each case for each chromosome, the CL-Brener type was the default type "A." Also, while two strains may have been assigned to the same CNV signature type for a given chromosome, they were not necessarily identical for that chromosome, as not every single CNV for every single chromosome was used in the typing (such an analysis would render every chromosome for every strain unique and make finding common patterns impossible). Note that chromosomes 4, 5, 18, 28, and 29 did not present sufficiently informative typing regions. Thus, all strains were type "A" for these chromosomes. Also, the CNV were haplotype specific. Therefore, in some cases this made the up or down calls (box color) appear incorrect, especially if the log2ratio for the feature was off the scale making it appear as if the boxed region is referring to the ot [file 1471-2164-12-139-S2.PPT]

## Slide 1
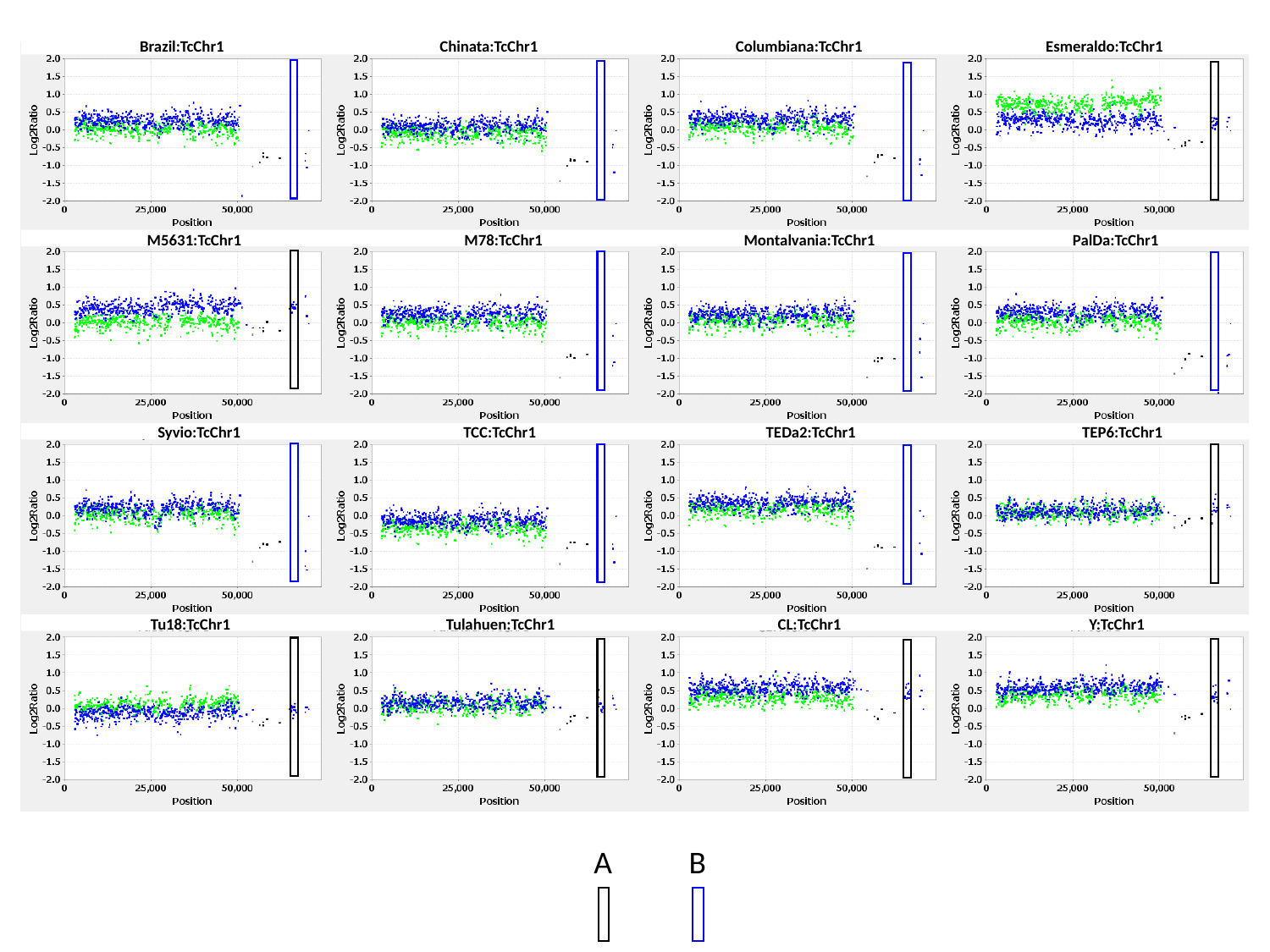

Brazil:TcChr1 Chinata:TcChr1 Columbiana:TcChr1 Esmeraldo:TcChr1
 M5631:TcChr1 M78:TcChr1 Montalvania:TcChr1 PalDa:TcChr1
 Syvio:TcChr1 TCC:TcChr1 TEDa2:TcChr1 TEP6:TcChr1
 Tu18:TcChr1 Tulahuen:TcChr1 CL:TcChr1 Y:TcChr1
A
B

## Slide 2
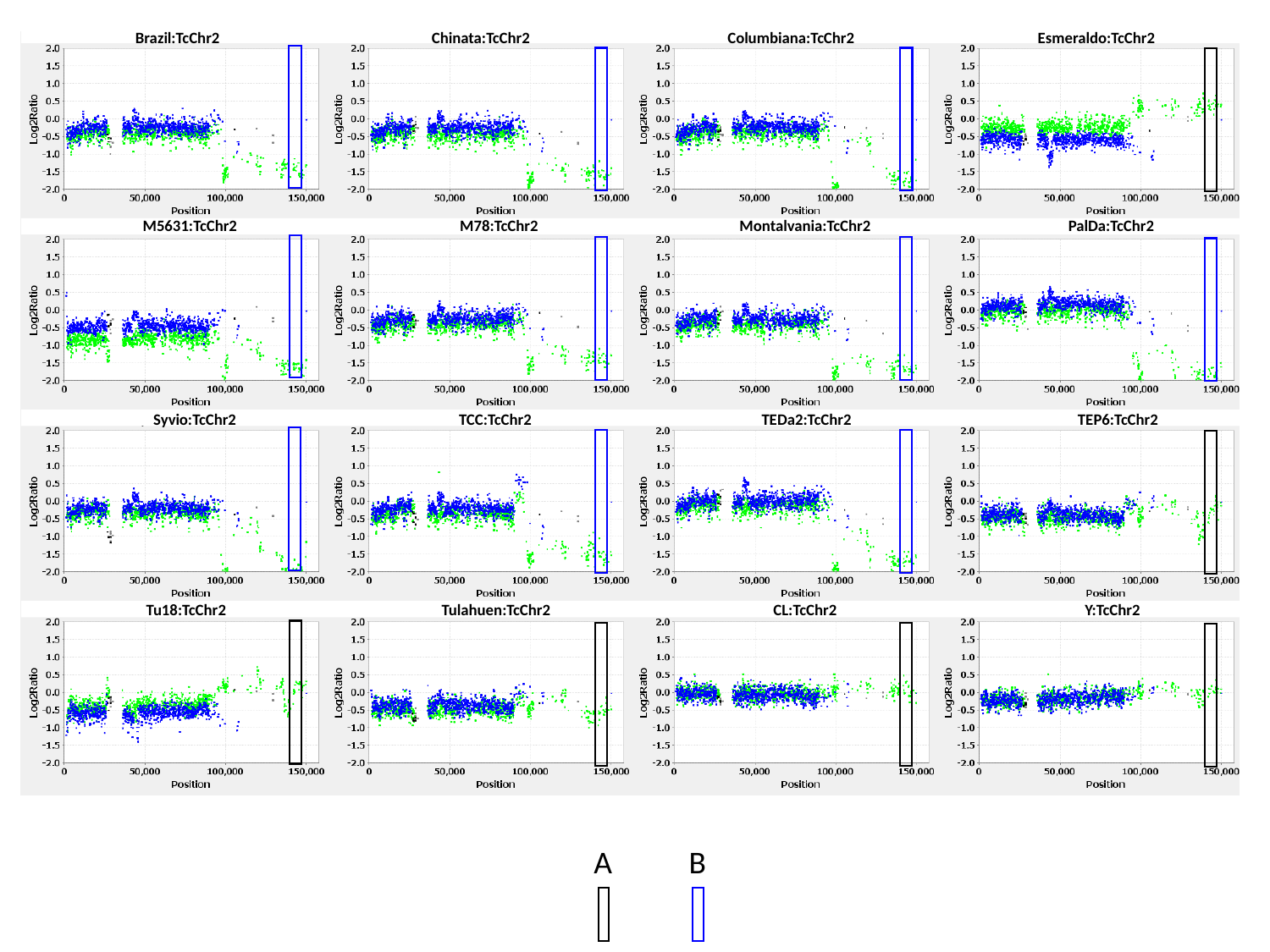

Brazil:TcChr2 Chinata:TcChr2 Columbiana:TcChr2 Esmeraldo:TcChr2
 M5631:TcChr2 M78:TcChr2 Montalvania:TcChr2 PalDa:TcChr2
 Syvio:TcChr2 TCC:TcChr2 TEDa2:TcChr2 TEP6:TcChr2
 Tu18:TcChr2 Tulahuen:TcChr2 CL:TcChr2 Y:TcChr2
A
B

## Slide 3
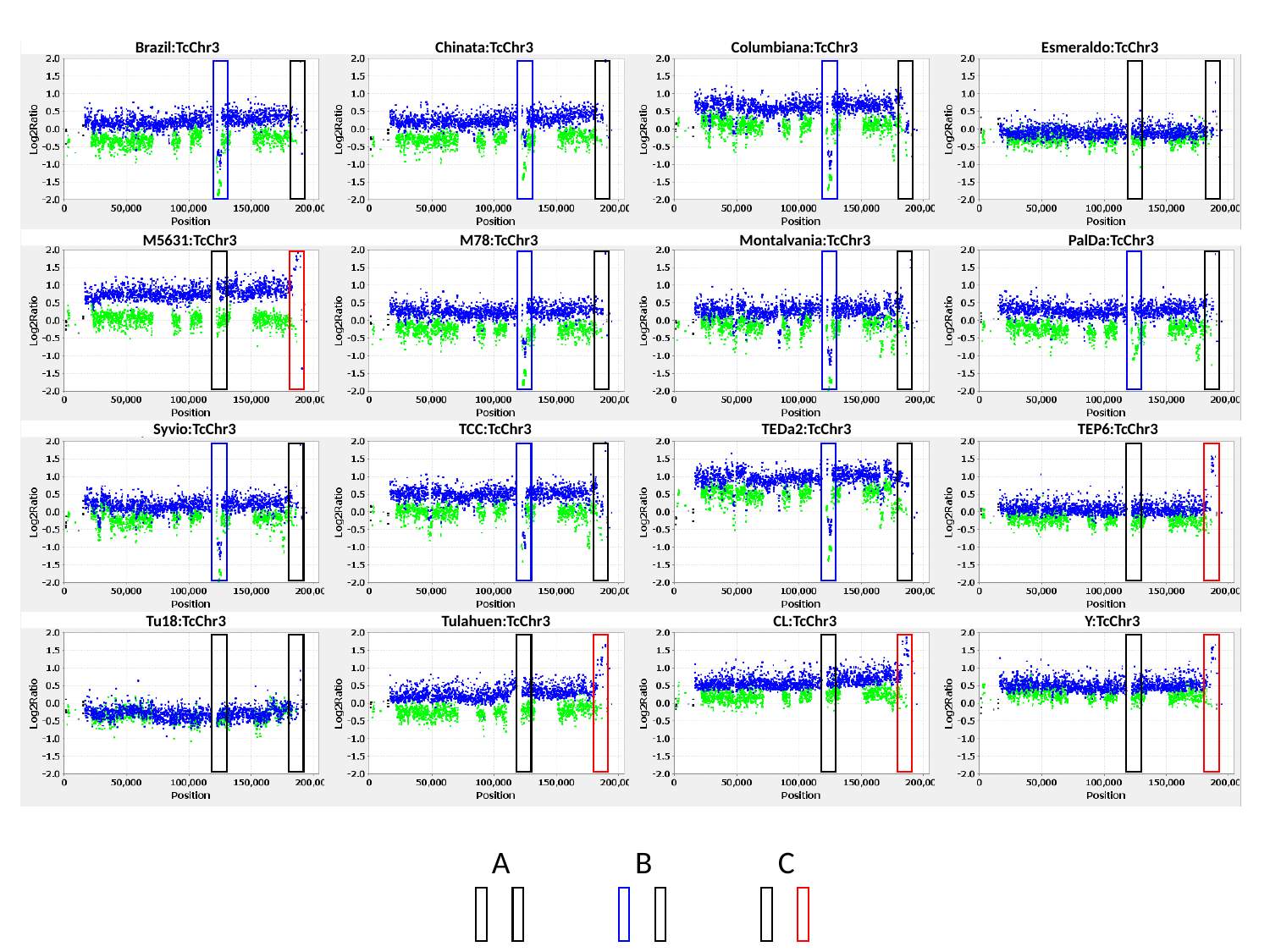

Brazil:TcChr3 Chinata:TcChr3 Columbiana:TcChr3 Esmeraldo:TcChr3
 M5631:TcChr3 M78:TcChr3 Montalvania:TcChr3 PalDa:TcChr3
 Syvio:TcChr3 TCC:TcChr3 TEDa2:TcChr3 TEP6:TcChr3
 Tu18:TcChr3 Tulahuen:TcChr3 CL:TcChr3 Y:TcChr3
A
B
C

## Slide 4
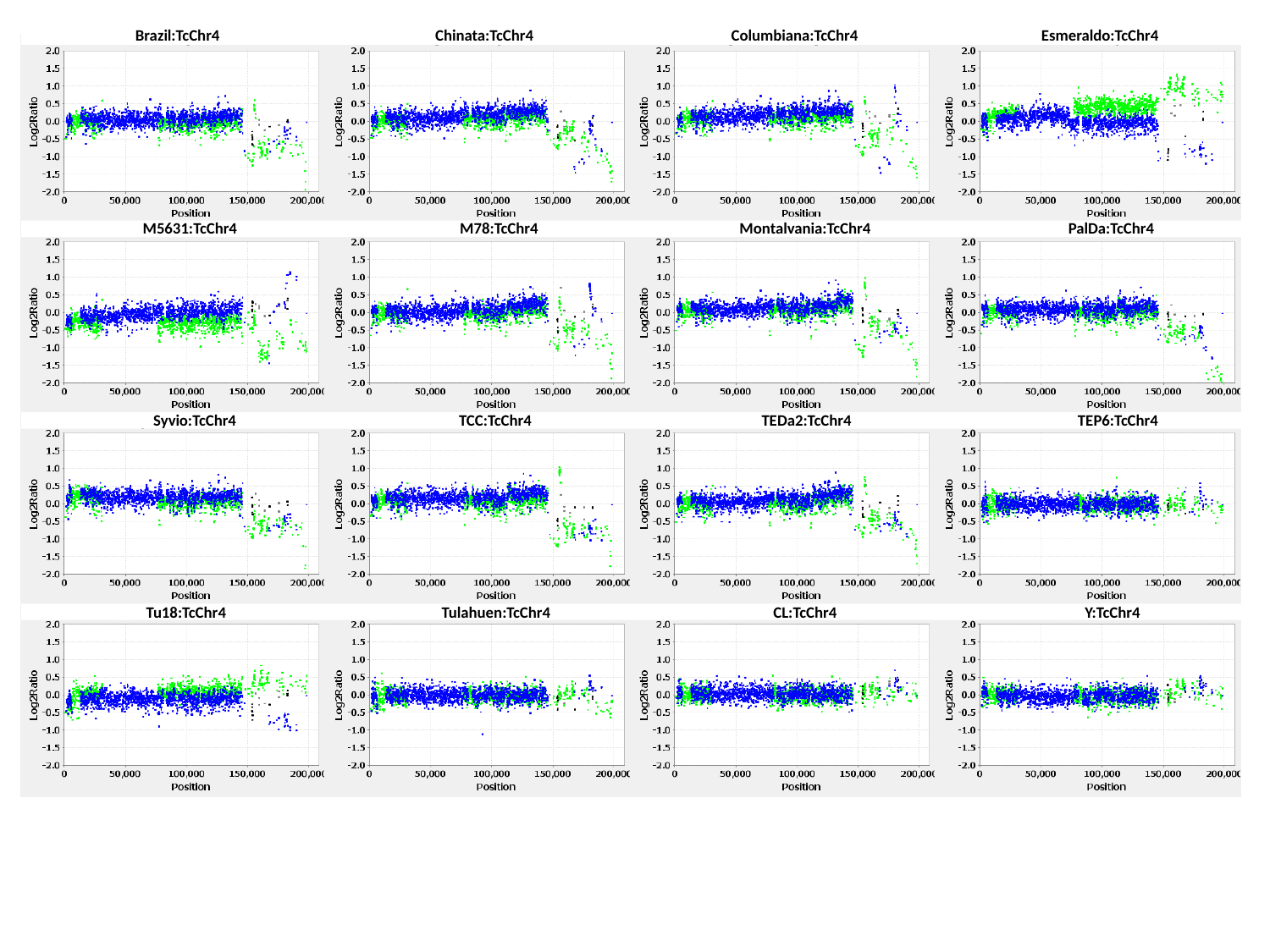

Brazil:TcChr4 Chinata:TcChr4 Columbiana:TcChr4 Esmeraldo:TcChr4
 M5631:TcChr4 M78:TcChr4 Montalvania:TcChr4 PalDa:TcChr4
 Syvio:TcChr4 TCC:TcChr4 TEDa2:TcChr4 TEP6:TcChr4
 Tu18:TcChr4 Tulahuen:TcChr4 CL:TcChr4 Y:TcChr4

## Slide 5
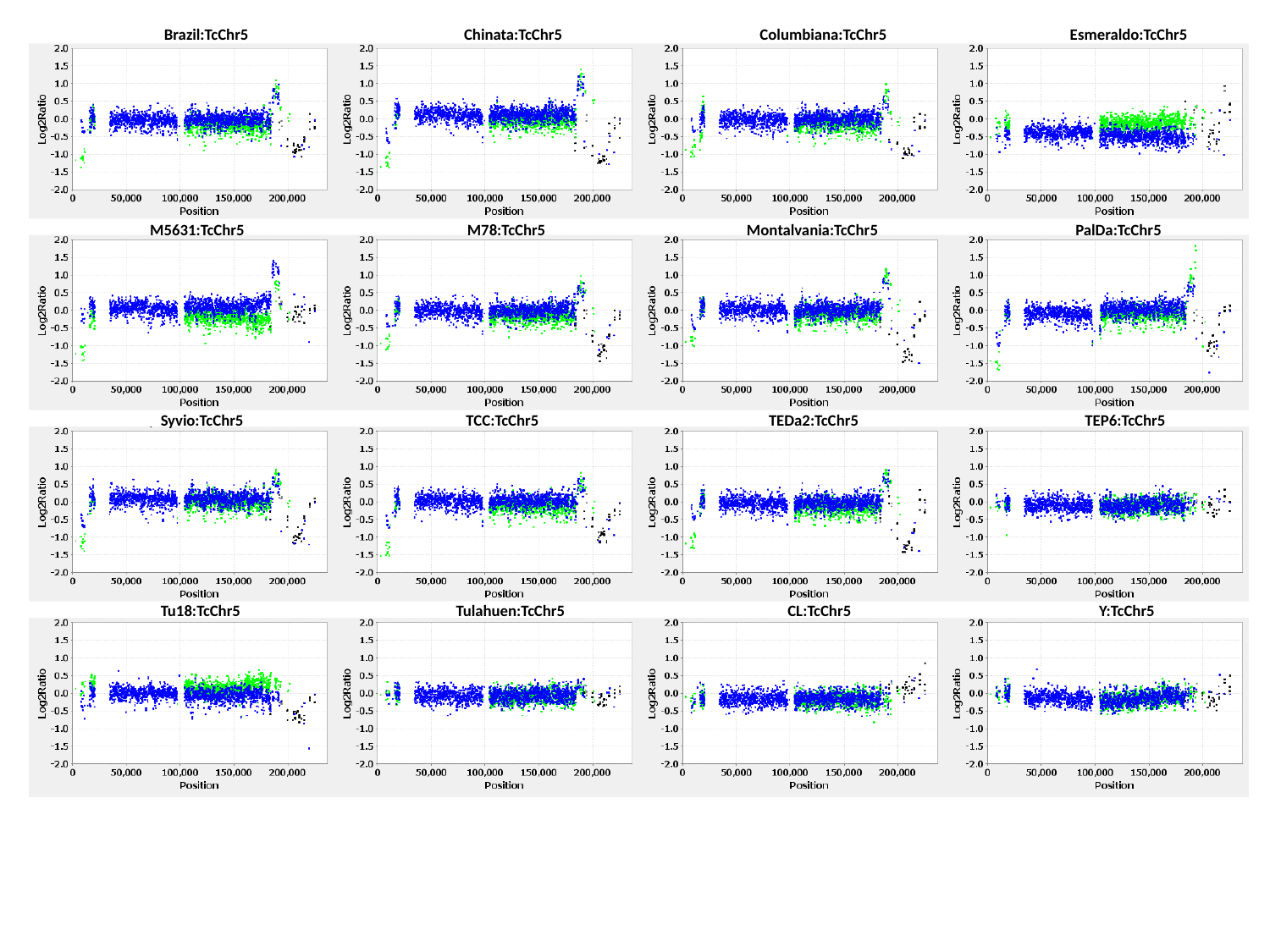

Brazil:TcChr5 Chinata:TcChr5 Columbiana:TcChr5 Esmeraldo:TcChr5
 M5631:TcChr5 M78:TcChr5 Montalvania:TcChr5 PalDa:TcChr5
 Syvio:TcChr5 TCC:TcChr5 TEDa2:TcChr5 TEP6:TcChr5
 Tu18:TcChr5 Tulahuen:TcChr5 CL:TcChr5 Y:TcChr5

## Slide 6
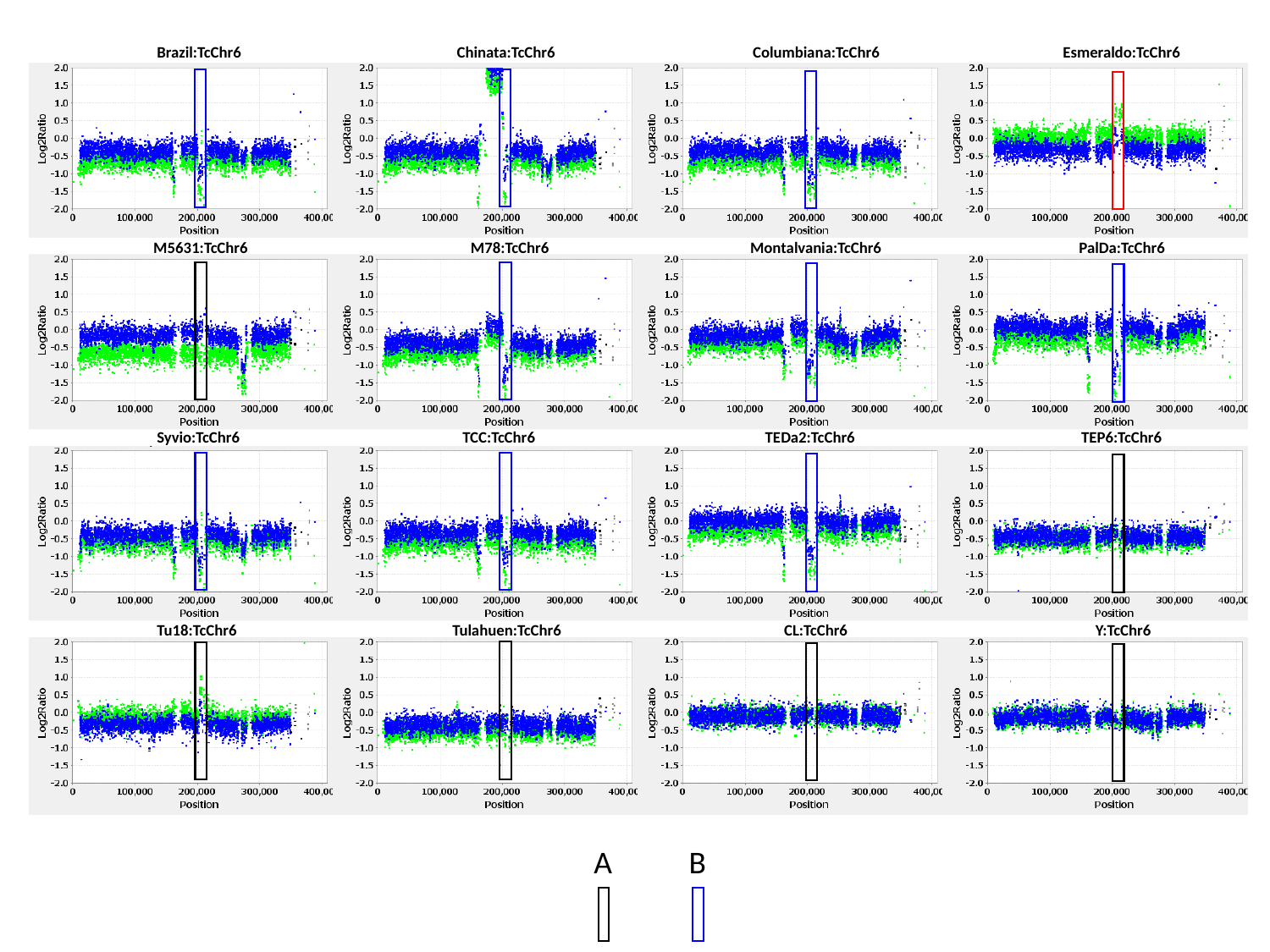

Brazil:TcChr6 Chinata:TcChr6 Columbiana:TcChr6 Esmeraldo:TcChr6
 M5631:TcChr6 M78:TcChr6 Montalvania:TcChr6 PalDa:TcChr6
 Syvio:TcChr6 TCC:TcChr6 TEDa2:TcChr6 TEP6:TcChr6
 Tu18:TcChr6 Tulahuen:TcChr6 CL:TcChr6 Y:TcChr6
A
B

## Slide 7
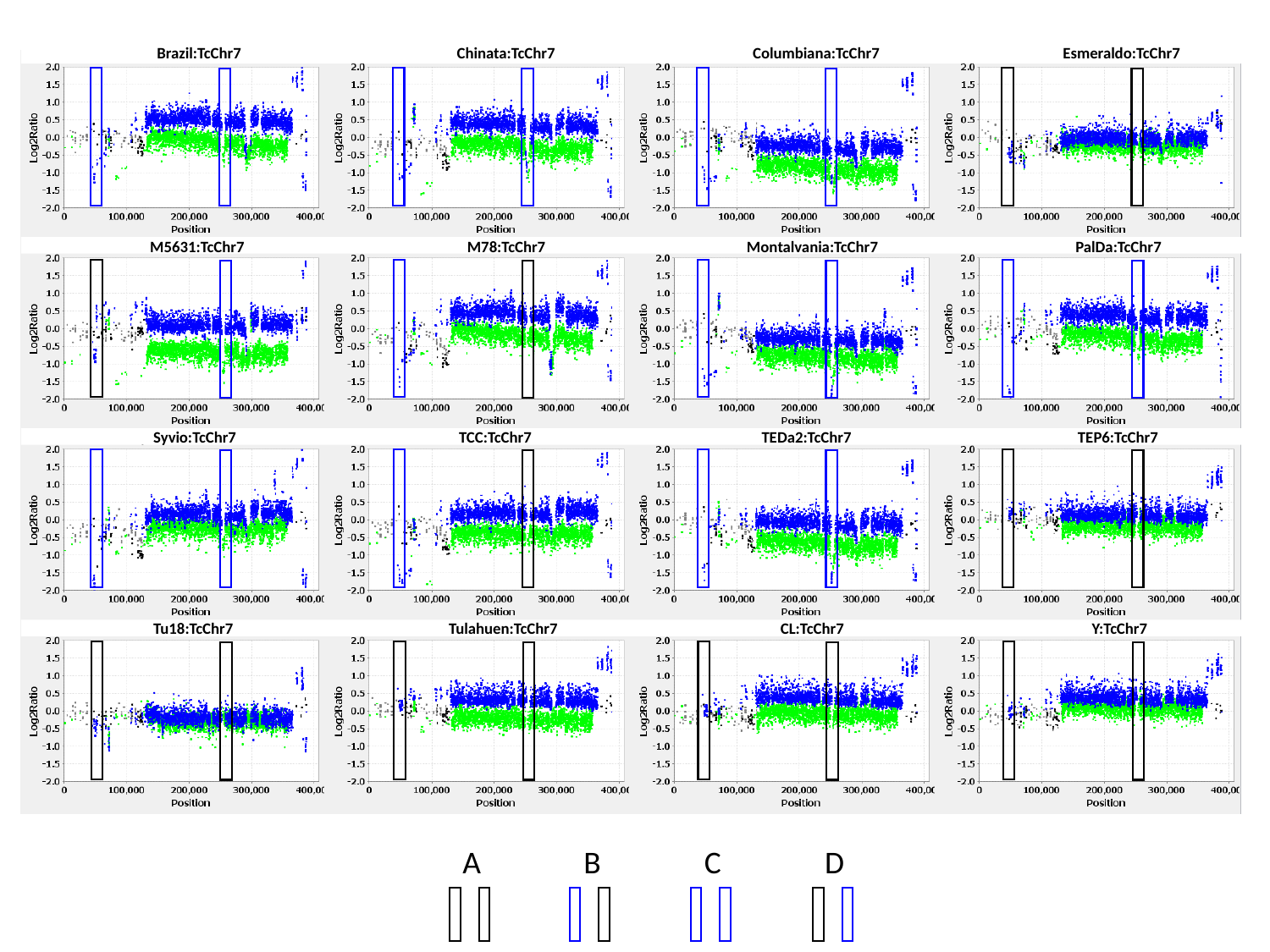

Brazil:TcChr7 Chinata:TcChr7 Columbiana:TcChr7 Esmeraldo:TcChr7
 M5631:TcChr7 M78:TcChr7 Montalvania:TcChr7 PalDa:TcChr7
 Syvio:TcChr7 TCC:TcChr7 TEDa2:TcChr7 TEP6:TcChr7
 Tu18:TcChr7 Tulahuen:TcChr7 CL:TcChr7 Y:TcChr7
A
B
C
D

## Slide 8
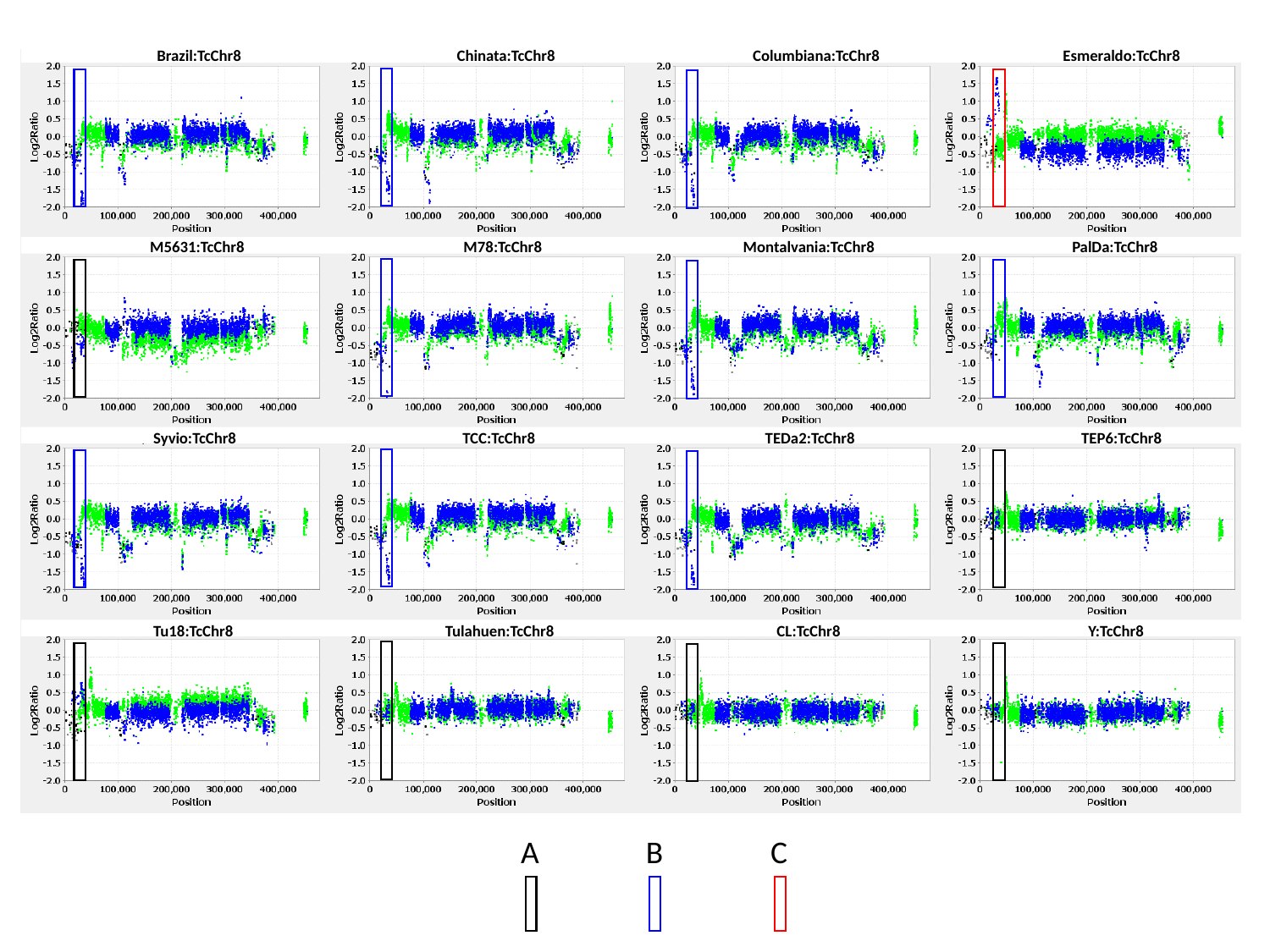

Brazil:TcChr8 Chinata:TcChr8 Columbiana:TcChr8 Esmeraldo:TcChr8
 M5631:TcChr8 M78:TcChr8 Montalvania:TcChr8 PalDa:TcChr8
 Syvio:TcChr8 TCC:TcChr8 TEDa2:TcChr8 TEP6:TcChr8
 Tu18:TcChr8 Tulahuen:TcChr8 CL:TcChr8 Y:TcChr8
A
B
C

## Slide 9
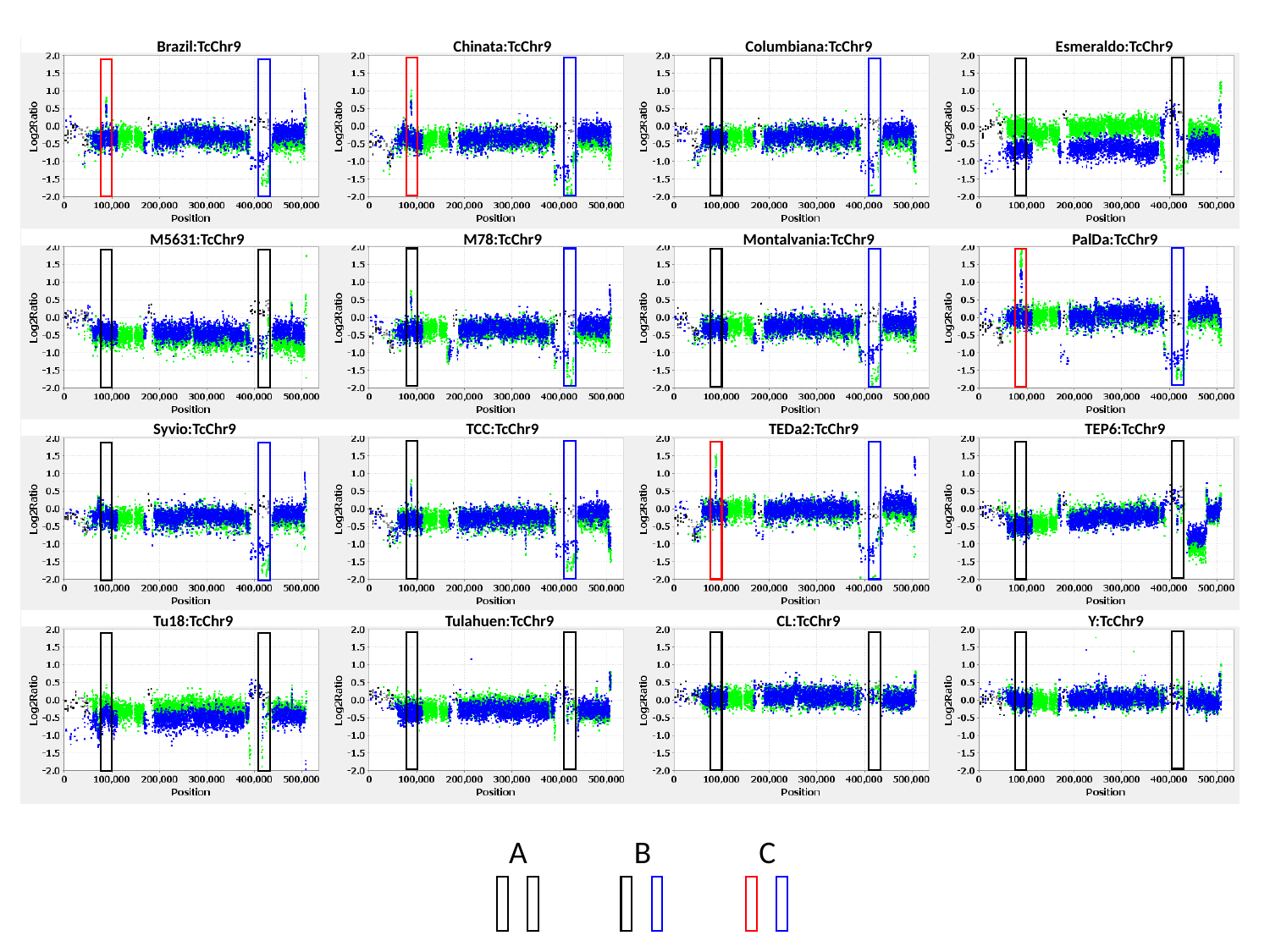

Brazil:TcChr9 Chinata:TcChr9 Columbiana:TcChr9 Esmeraldo:TcChr9
 M5631:TcChr9 M78:TcChr9 Montalvania:TcChr9 PalDa:TcChr9
 Syvio:TcChr9 TCC:TcChr9 TEDa2:TcChr9 TEP6:TcChr9
 Tu18:TcChr9 Tulahuen:TcChr9 CL:TcChr9 Y:TcChr9
A
B
C

## Slide 10
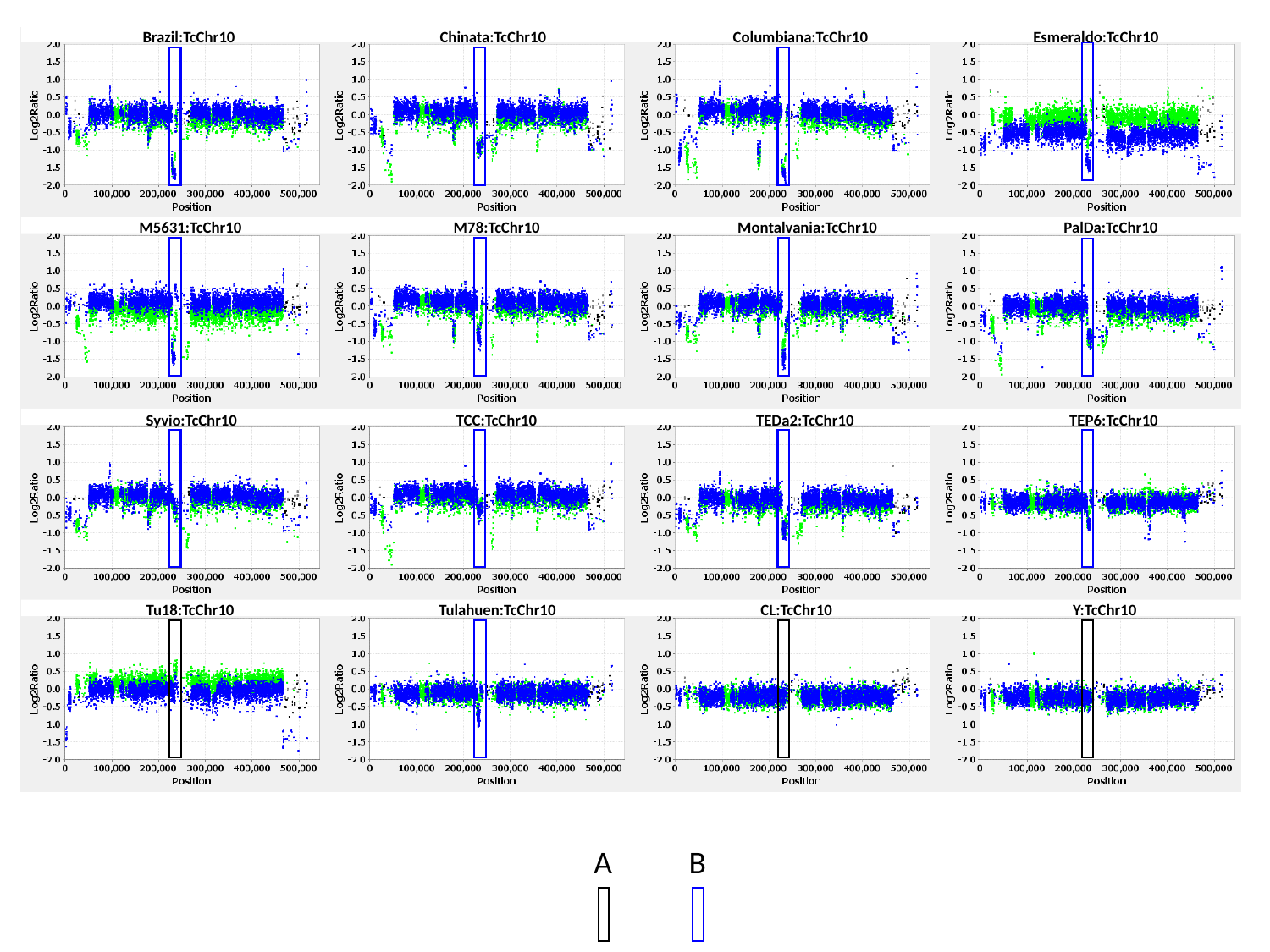

Brazil:TcChr10 Chinata:TcChr10 Columbiana:TcChr10 Esmeraldo:TcChr10
 M5631:TcChr10 M78:TcChr10 Montalvania:TcChr10 PalDa:TcChr10
 Syvio:TcChr10 TCC:TcChr10 TEDa2:TcChr10 TEP6:TcChr10
 Tu18:TcChr10 Tulahuen:TcChr10 CL:TcChr10 Y:TcChr10
A
B

## Slide 11
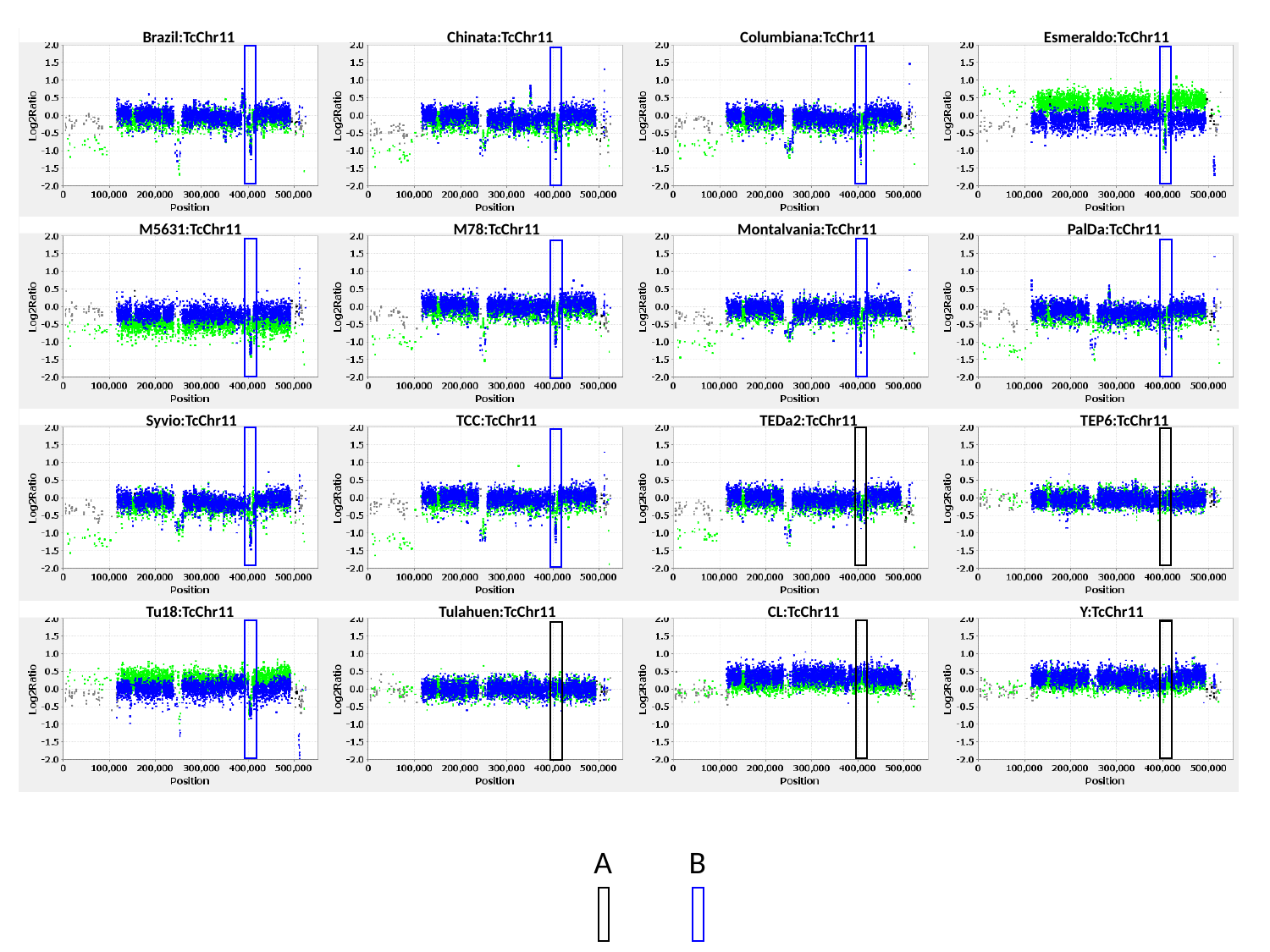

Brazil:TcChr11 Chinata:TcChr11 Columbiana:TcChr11 Esmeraldo:TcChr11
 M5631:TcChr11 M78:TcChr11 Montalvania:TcChr11 PalDa:TcChr11
 Syvio:TcChr11 TCC:TcChr11 TEDa2:TcChr11 TEP6:TcChr11
 Tu18:TcChr11 Tulahuen:TcChr11 CL:TcChr11 Y:TcChr11
A
B

## Slide 12
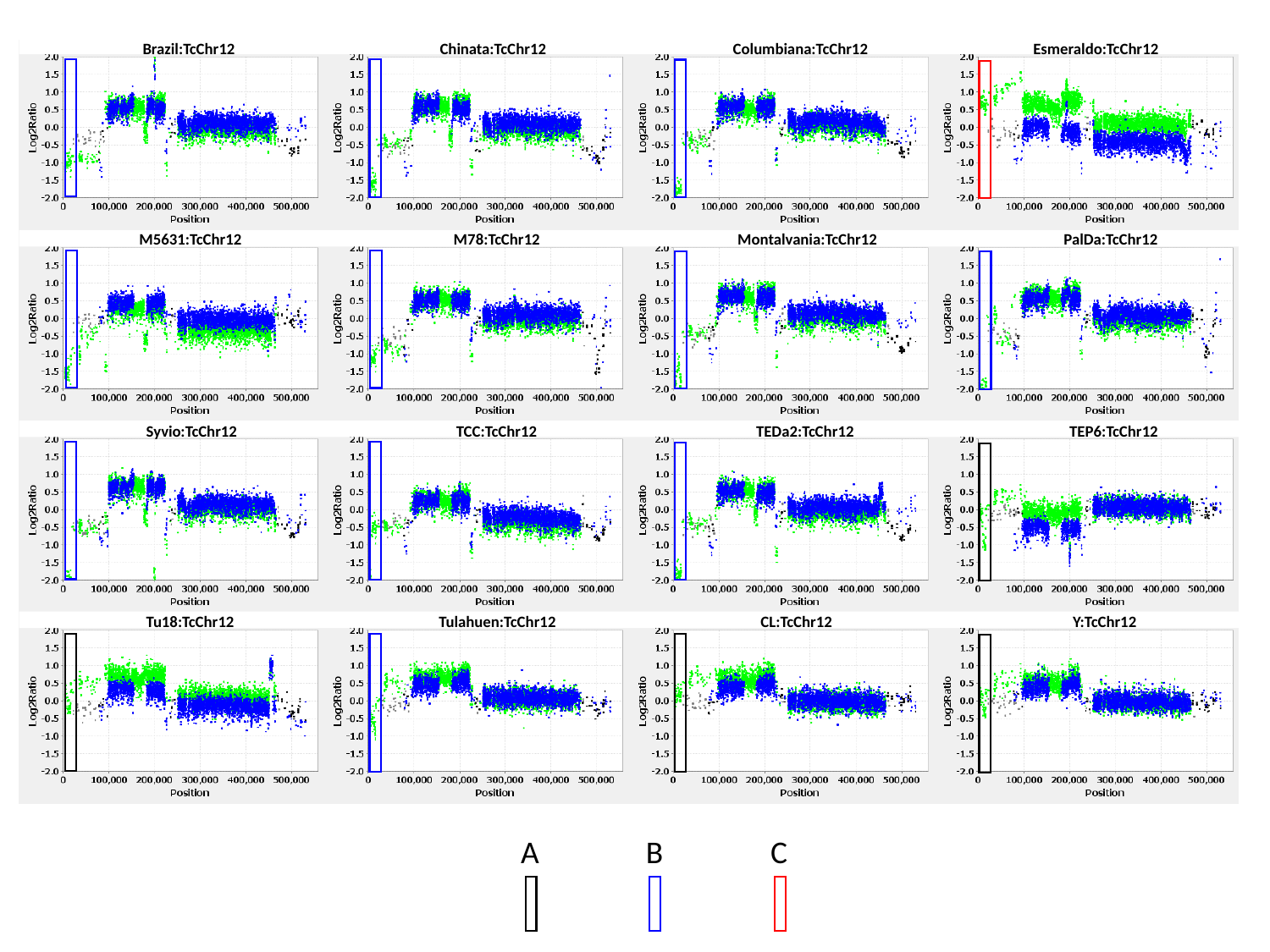

Brazil:TcChr12 Chinata:TcChr12 Columbiana:TcChr12 Esmeraldo:TcChr12
 M5631:TcChr12 M78:TcChr12 Montalvania:TcChr12 PalDa:TcChr12
 Syvio:TcChr12 TCC:TcChr12 TEDa2:TcChr12 TEP6:TcChr12
 Tu18:TcChr12 Tulahuen:TcChr12 CL:TcChr12 Y:TcChr12
A
B
C

## Slide 13
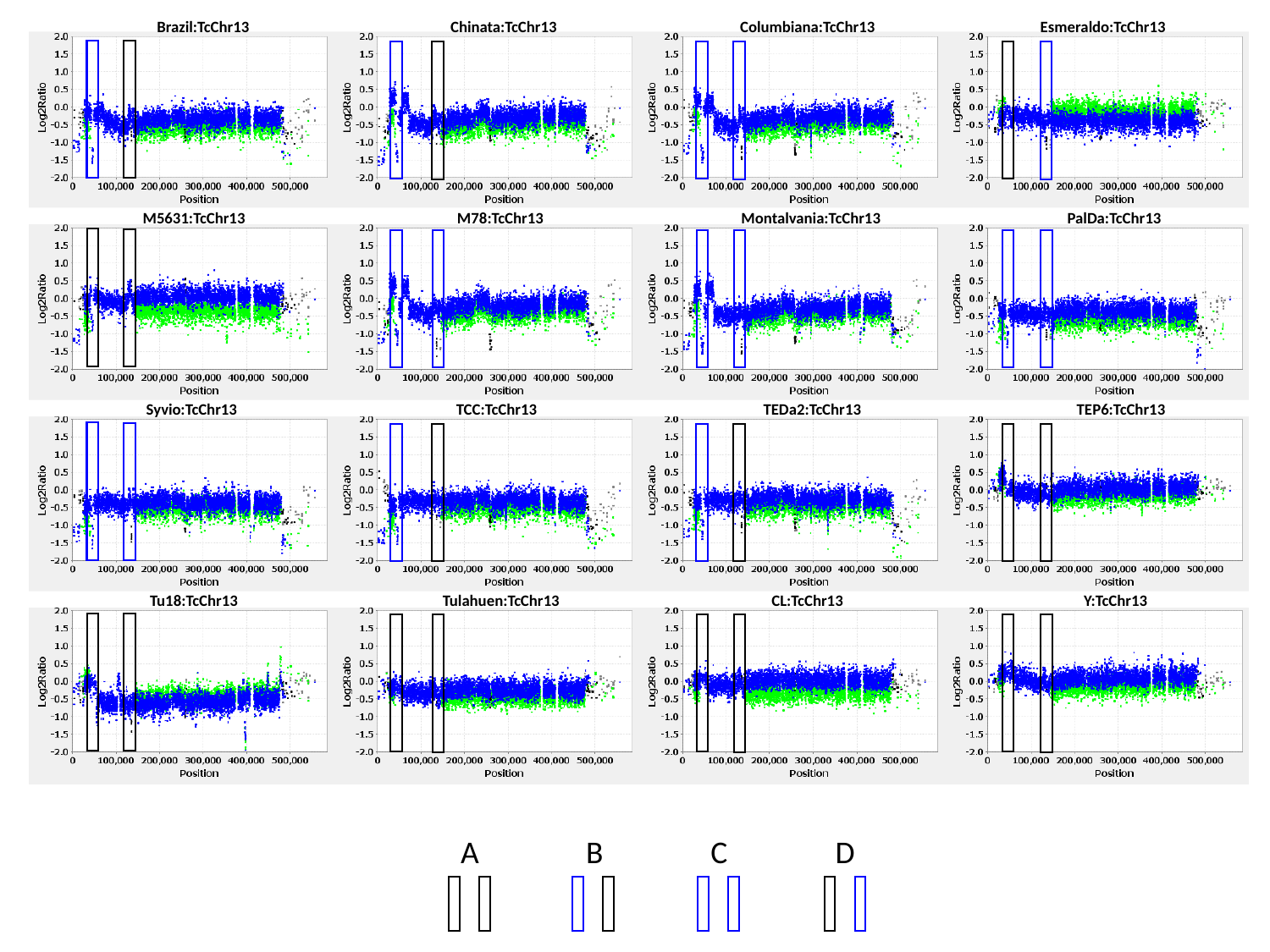

Brazil:TcChr13 Chinata:TcChr13 Columbiana:TcChr13 Esmeraldo:TcChr13
 M5631:TcChr13 M78:TcChr13 Montalvania:TcChr13 PalDa:TcChr13
 Syvio:TcChr13 TCC:TcChr13 TEDa2:TcChr13 TEP6:TcChr13
 Tu18:TcChr13 Tulahuen:TcChr13 CL:TcChr13 Y:TcChr13
A
B
C
D

## Slide 14
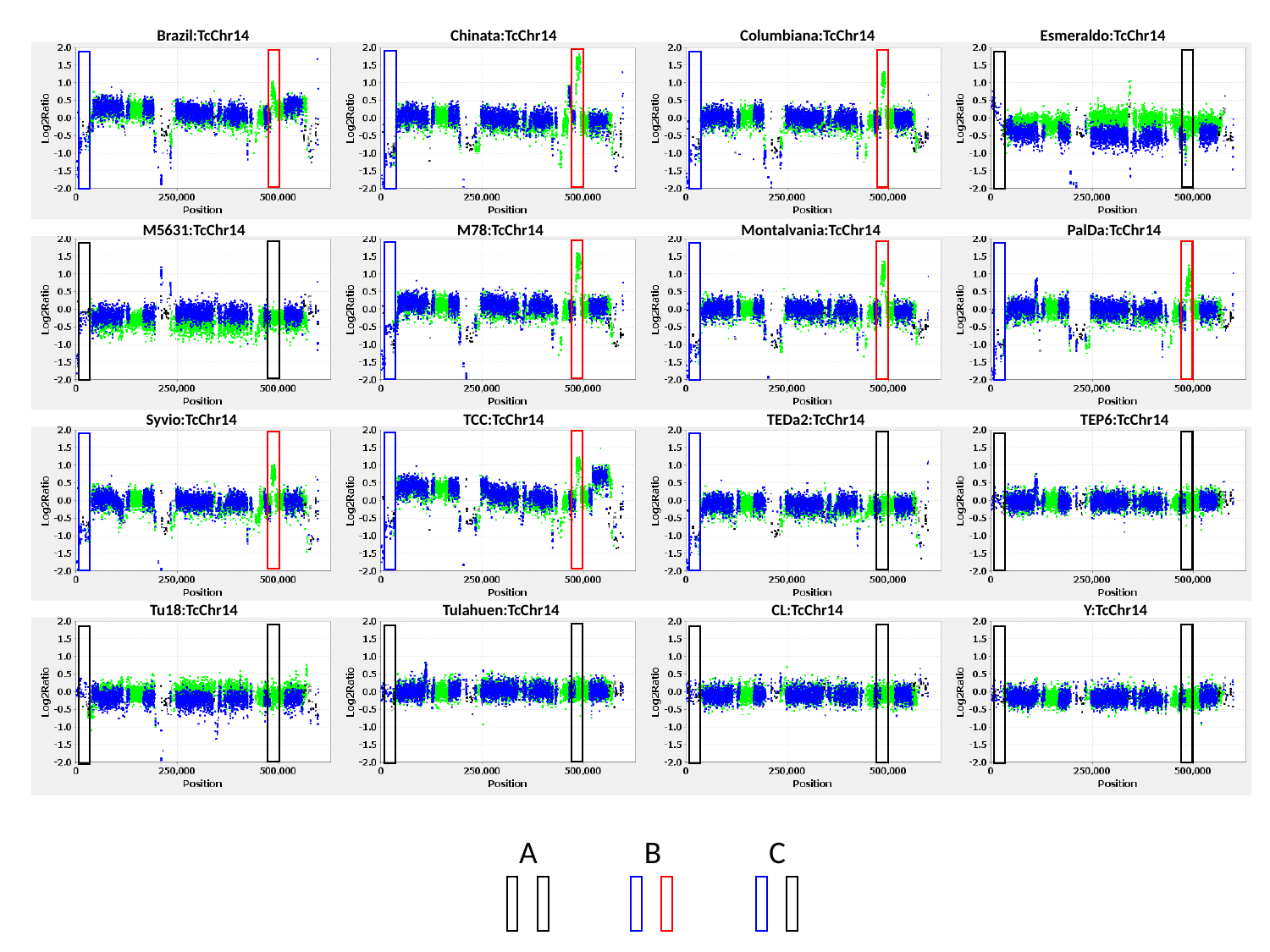

Brazil:TcChr14 Chinata:TcChr14 Columbiana:TcChr14 Esmeraldo:TcChr14
 M5631:TcChr14 M78:TcChr14 Montalvania:TcChr14 PalDa:TcChr14
 Syvio:TcChr14 TCC:TcChr14 TEDa2:TcChr14 TEP6:TcChr14
 Tu18:TcChr14 Tulahuen:TcChr14 CL:TcChr14 Y:TcChr14
A
B
C

## Slide 15
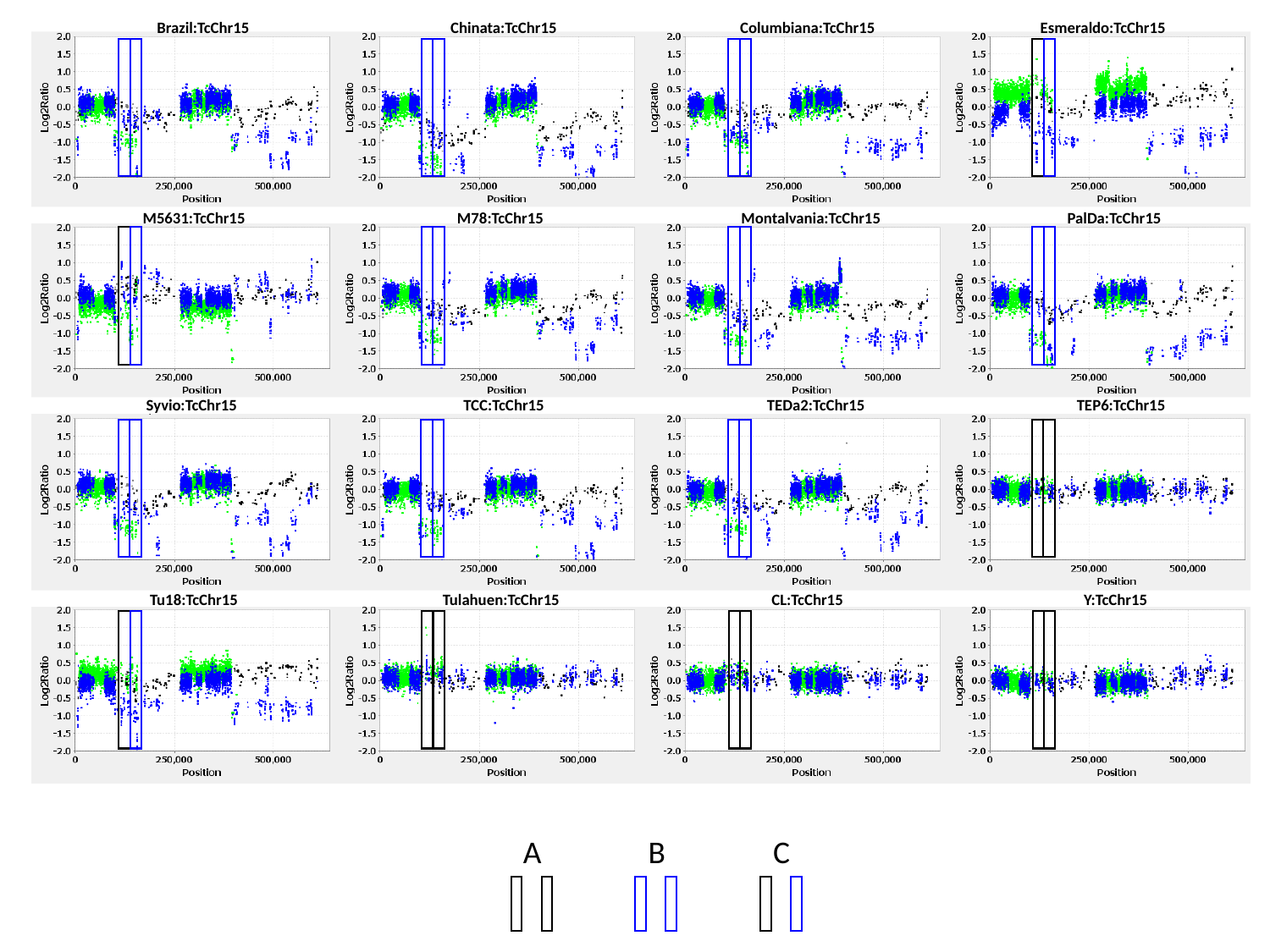

Brazil:TcChr15 Chinata:TcChr15 Columbiana:TcChr15 Esmeraldo:TcChr15
 M5631:TcChr15 M78:TcChr15 Montalvania:TcChr15 PalDa:TcChr15
 Syvio:TcChr15 TCC:TcChr15 TEDa2:TcChr15 TEP6:TcChr15
 Tu18:TcChr15 Tulahuen:TcChr15 CL:TcChr15 Y:TcChr15
A
B
C

## Slide 16
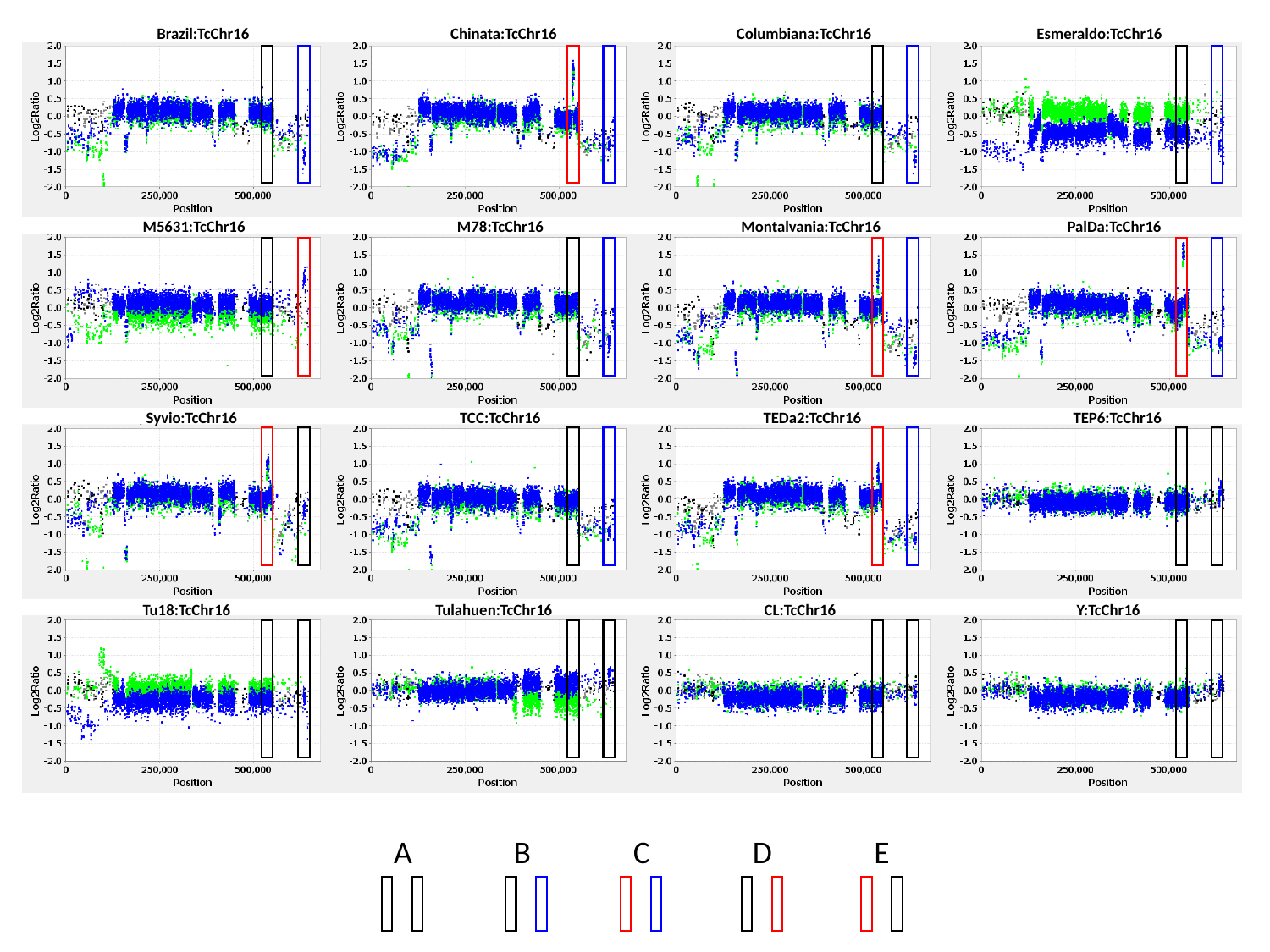

Brazil:TcChr16 Chinata:TcChr16 Columbiana:TcChr16 Esmeraldo:TcChr16
 M5631:TcChr16 M78:TcChr16 Montalvania:TcChr16 PalDa:TcChr16
 Syvio:TcChr16 TCC:TcChr16 TEDa2:TcChr16 TEP6:TcChr16
 Tu18:TcChr16 Tulahuen:TcChr16 CL:TcChr16 Y:TcChr16
A
B
C
D
E

## Slide 17
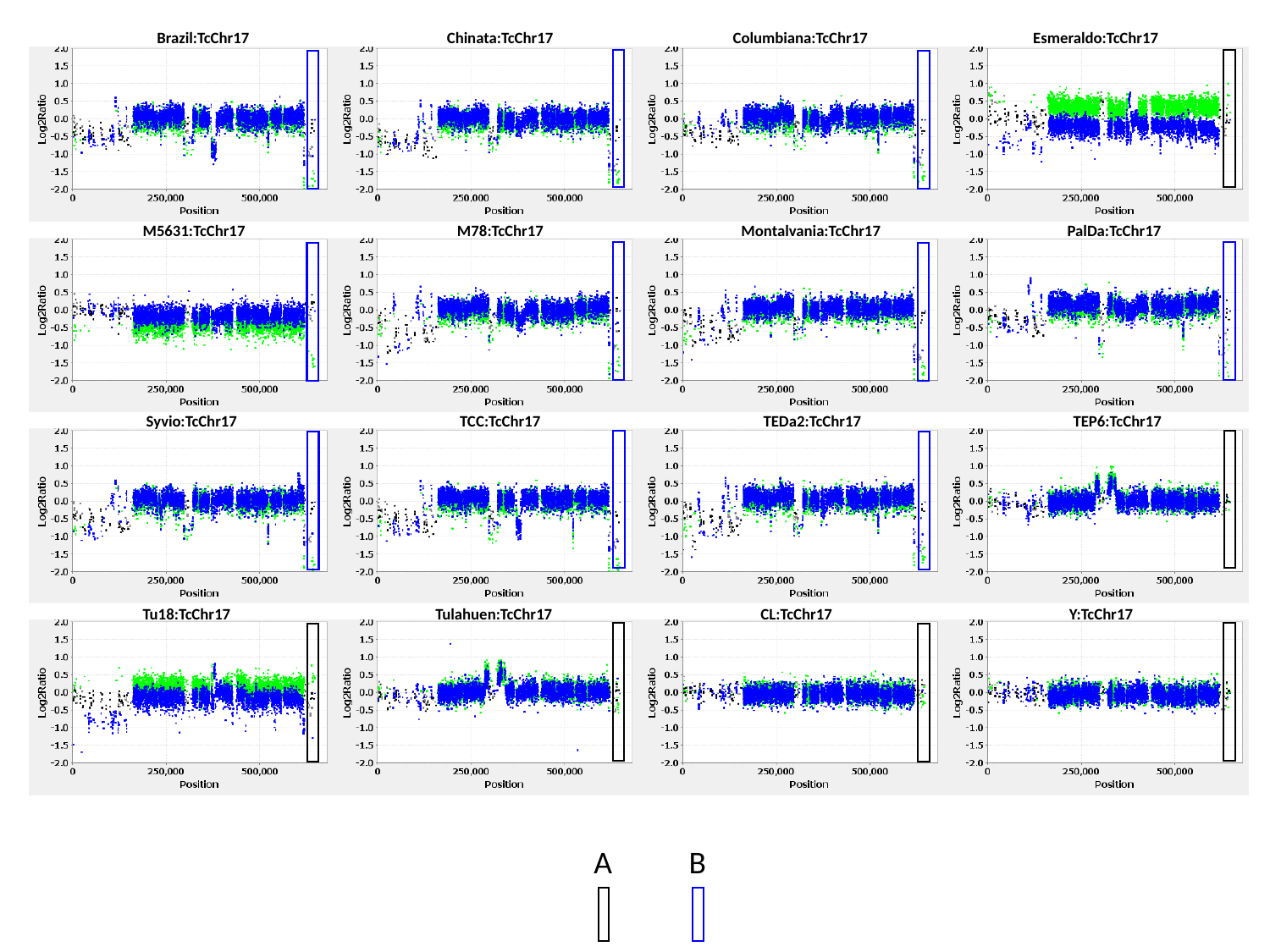

Brazil:TcChr17 Chinata:TcChr17 Columbiana:TcChr17 Esmeraldo:TcChr17
 M5631:TcChr17 M78:TcChr17 Montalvania:TcChr17 PalDa:TcChr17
 Syvio:TcChr17 TCC:TcChr17 TEDa2:TcChr17 TEP6:TcChr17
 Tu18:TcChr17 Tulahuen:TcChr17 CL:TcChr17 Y:TcChr17
A
B

## Slide 18
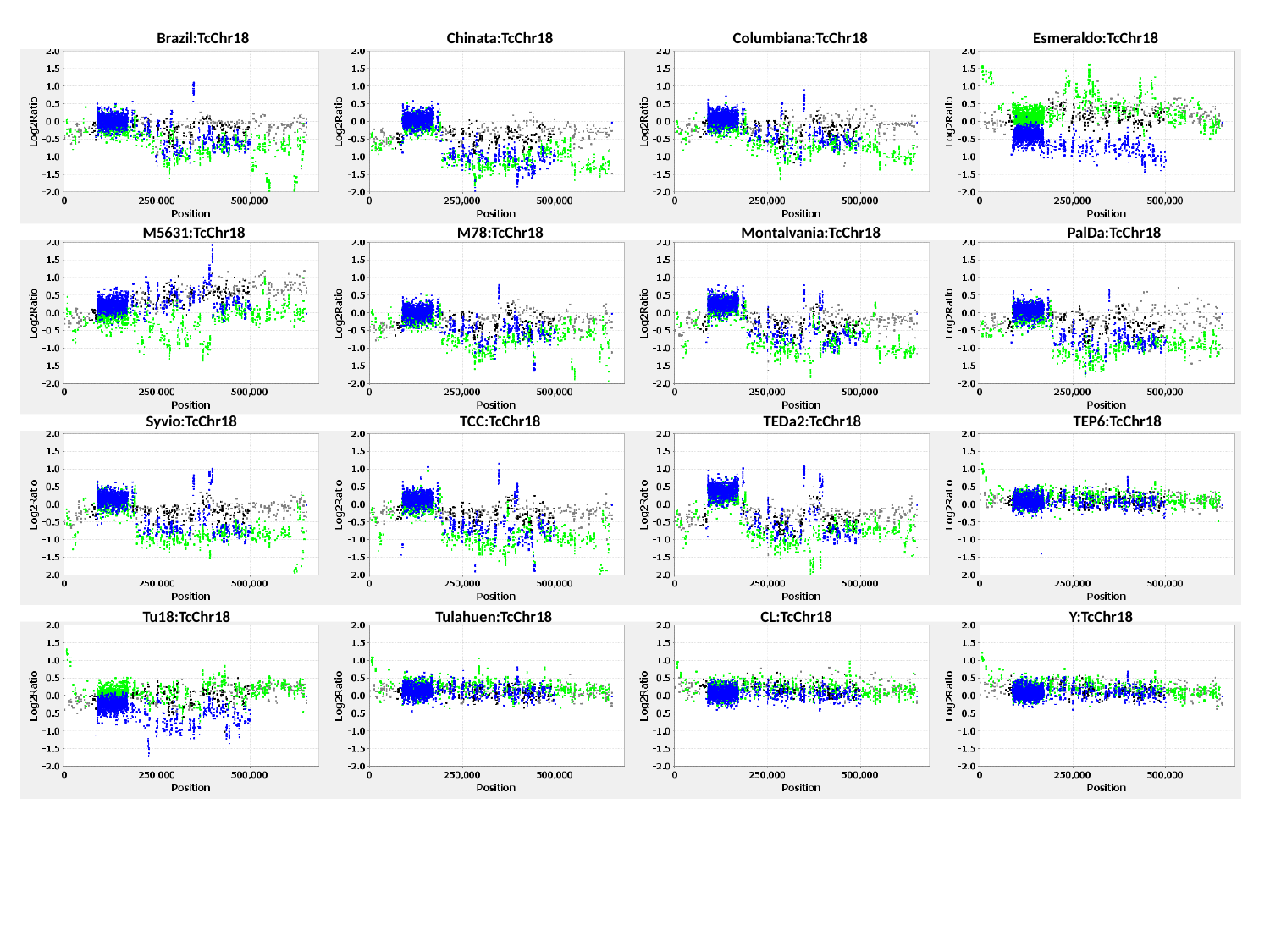

Brazil:TcChr18 Chinata:TcChr18 Columbiana:TcChr18 Esmeraldo:TcChr18
 M5631:TcChr18 M78:TcChr18 Montalvania:TcChr18 PalDa:TcChr18
 Syvio:TcChr18 TCC:TcChr18 TEDa2:TcChr18 TEP6:TcChr18
 Tu18:TcChr18 Tulahuen:TcChr18 CL:TcChr18 Y:TcChr18

## Slide 19
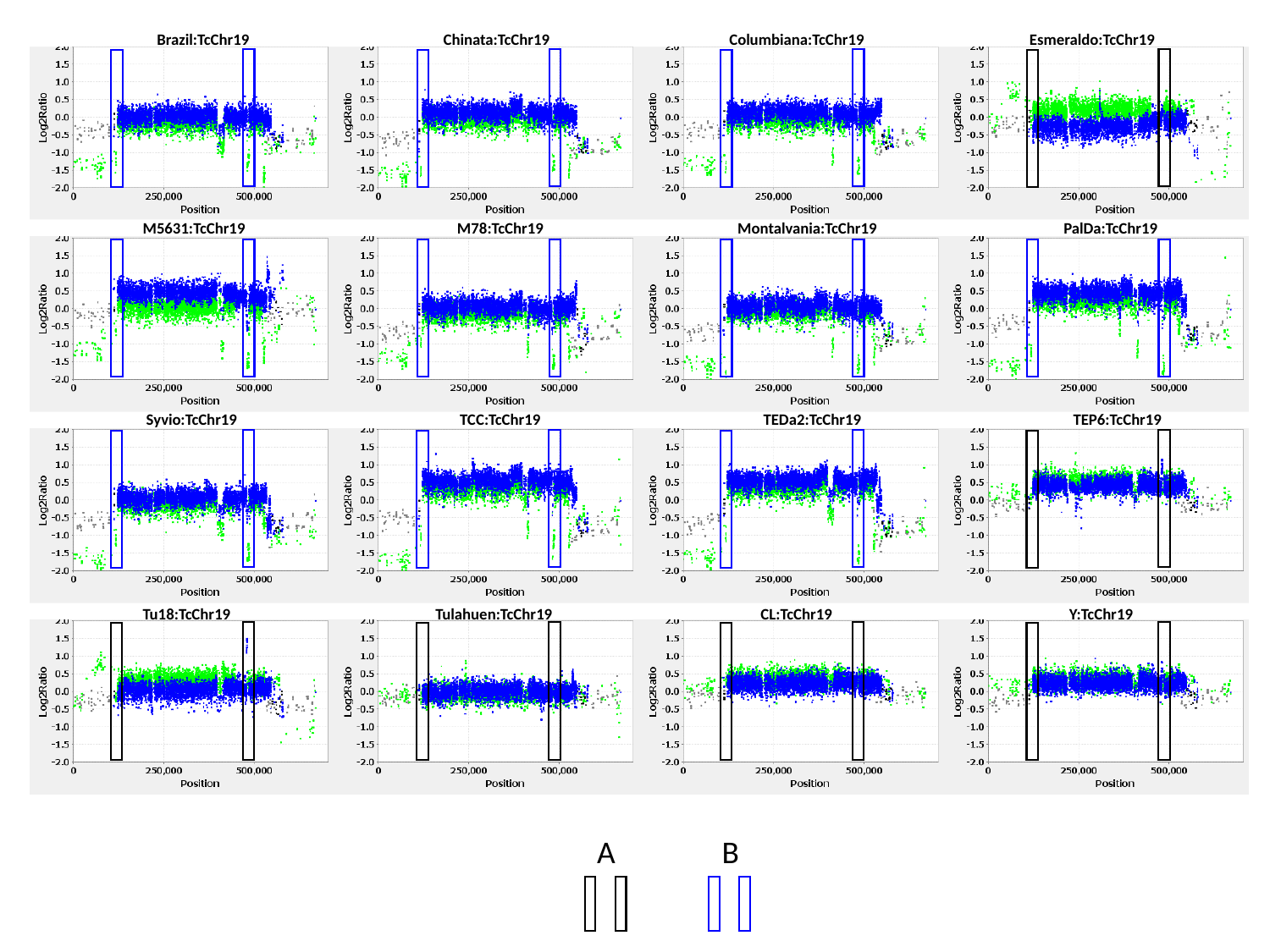

Brazil:TcChr19 Chinata:TcChr19 Columbiana:TcChr19 Esmeraldo:TcChr19
 M5631:TcChr19 M78:TcChr19 Montalvania:TcChr19 PalDa:TcChr19
 Syvio:TcChr19 TCC:TcChr19 TEDa2:TcChr19 TEP6:TcChr19
 Tu18:TcChr19 Tulahuen:TcChr19 CL:TcChr19 Y:TcChr19
A
B

## Slide 20
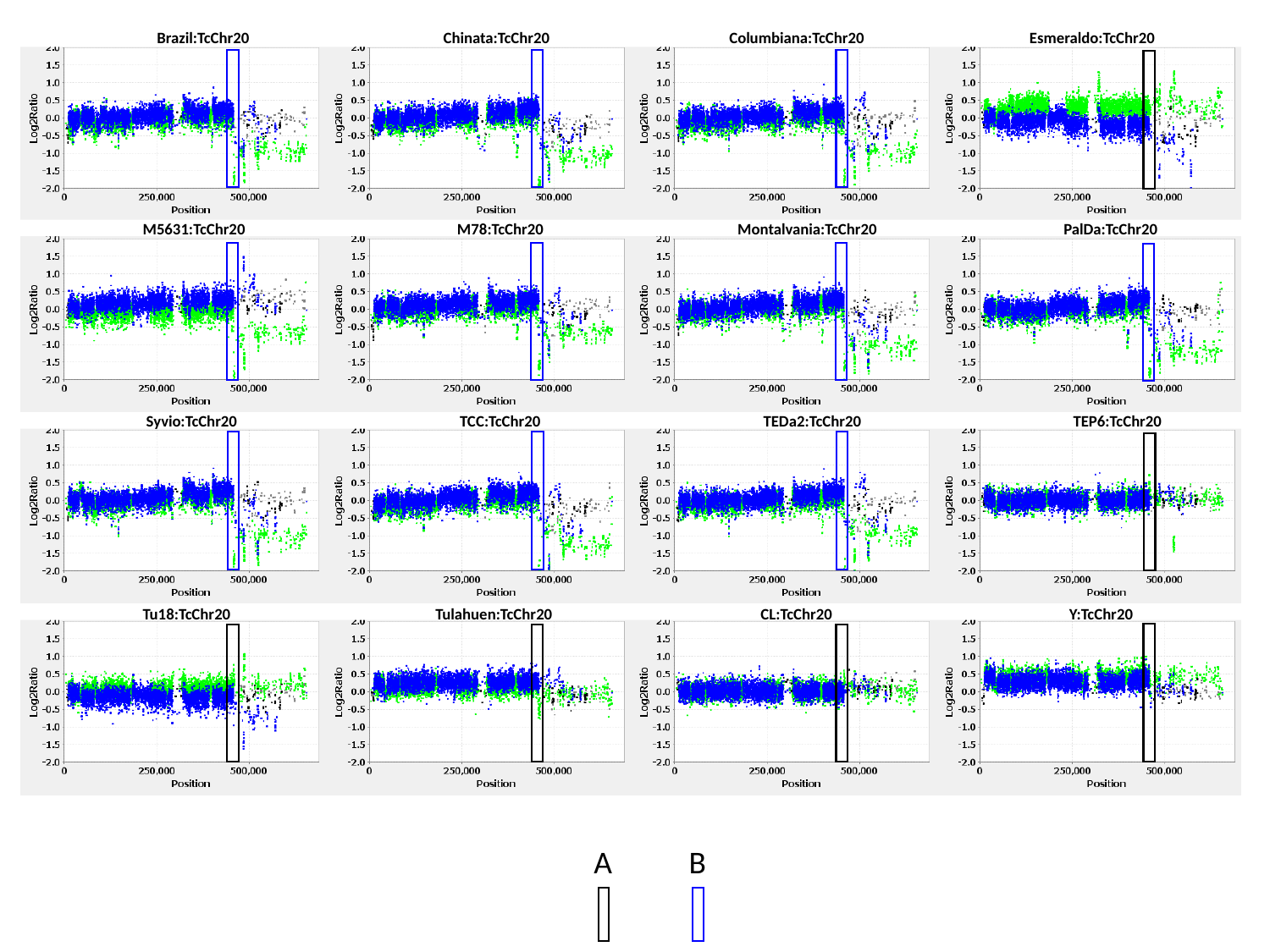

Brazil:TcChr20 Chinata:TcChr20 Columbiana:TcChr20 Esmeraldo:TcChr20
 M5631:TcChr20 M78:TcChr20 Montalvania:TcChr20 PalDa:TcChr20
 Syvio:TcChr20 TCC:TcChr20 TEDa2:TcChr20 TEP6:TcChr20
 Tu18:TcChr20 Tulahuen:TcChr20 CL:TcChr20 Y:TcChr20
A
B

## Slide 21
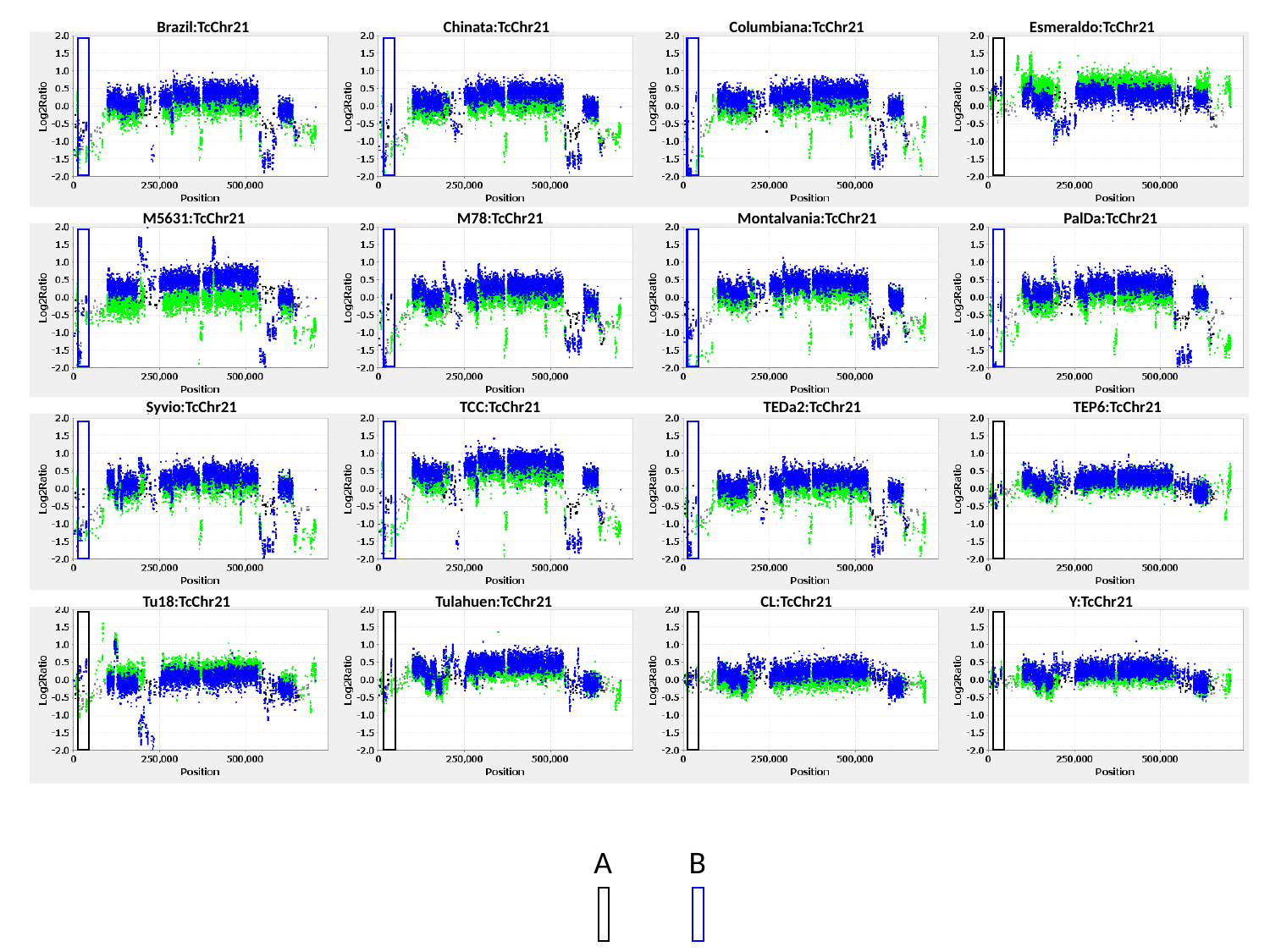

Brazil:TcChr21 Chinata:TcChr21 Columbiana:TcChr21 Esmeraldo:TcChr21
 M5631:TcChr21 M78:TcChr21 Montalvania:TcChr21 PalDa:TcChr21
 Syvio:TcChr21 TCC:TcChr21 TEDa2:TcChr21 TEP6:TcChr21
 Tu18:TcChr21 Tulahuen:TcChr21 CL:TcChr21 Y:TcChr21
A
B

## Slide 22
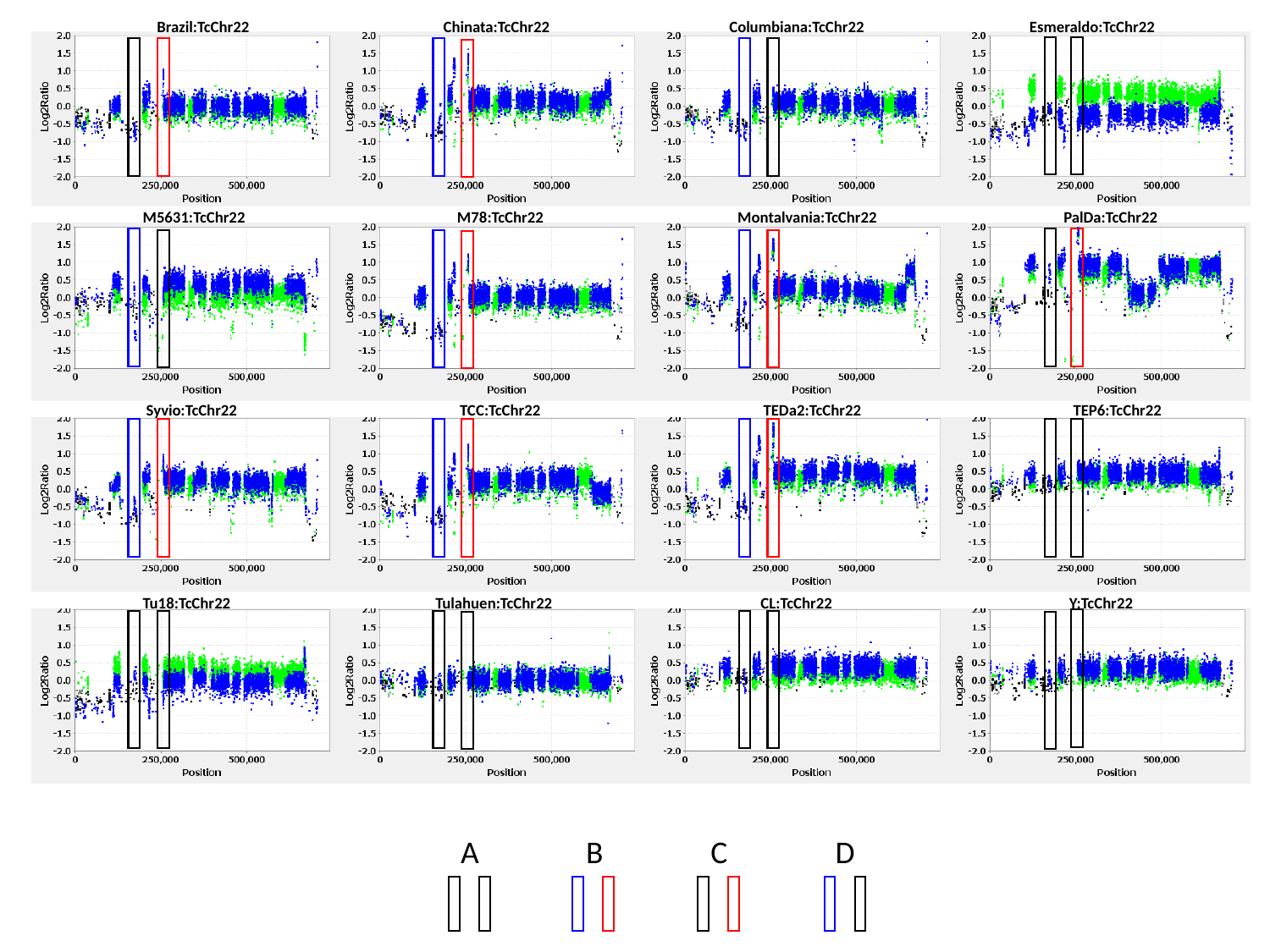

Brazil:TcChr22 Chinata:TcChr22 Columbiana:TcChr22 Esmeraldo:TcChr22
 M5631:TcChr22 M78:TcChr22 Montalvania:TcChr22 PalDa:TcChr22
 Syvio:TcChr22 TCC:TcChr22 TEDa2:TcChr22 TEP6:TcChr22
 Tu18:TcChr22 Tulahuen:TcChr22 CL:TcChr22 Y:TcChr22
A
B
C
D

## Slide 23
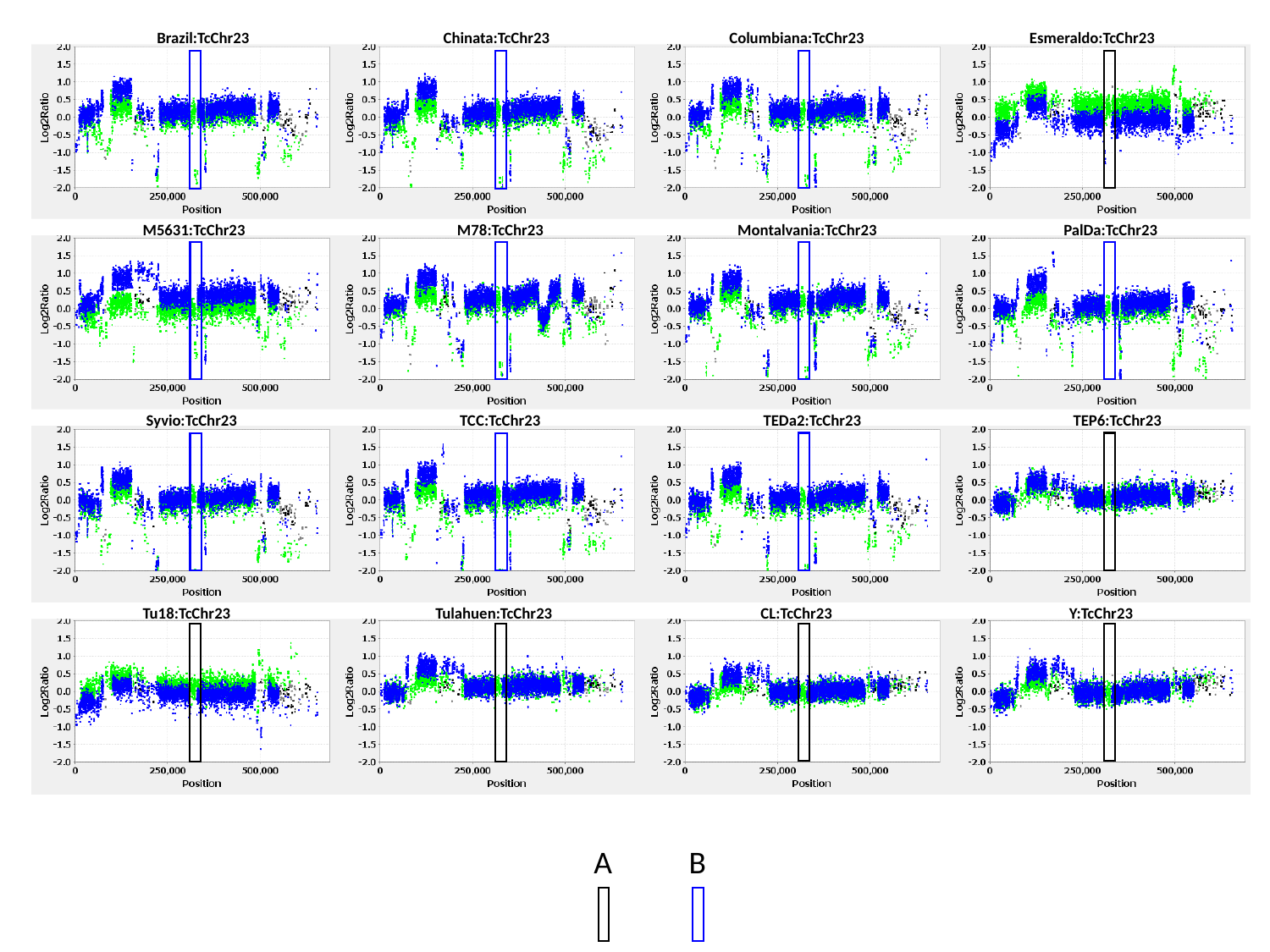

Brazil:TcChr23 Chinata:TcChr23 Columbiana:TcChr23 Esmeraldo:TcChr23
 M5631:TcChr23 M78:TcChr23 Montalvania:TcChr23 PalDa:TcChr23
 Syvio:TcChr23 TCC:TcChr23 TEDa2:TcChr23 TEP6:TcChr23
 Tu18:TcChr23 Tulahuen:TcChr23 CL:TcChr23 Y:TcChr23
A
B

## Slide 24
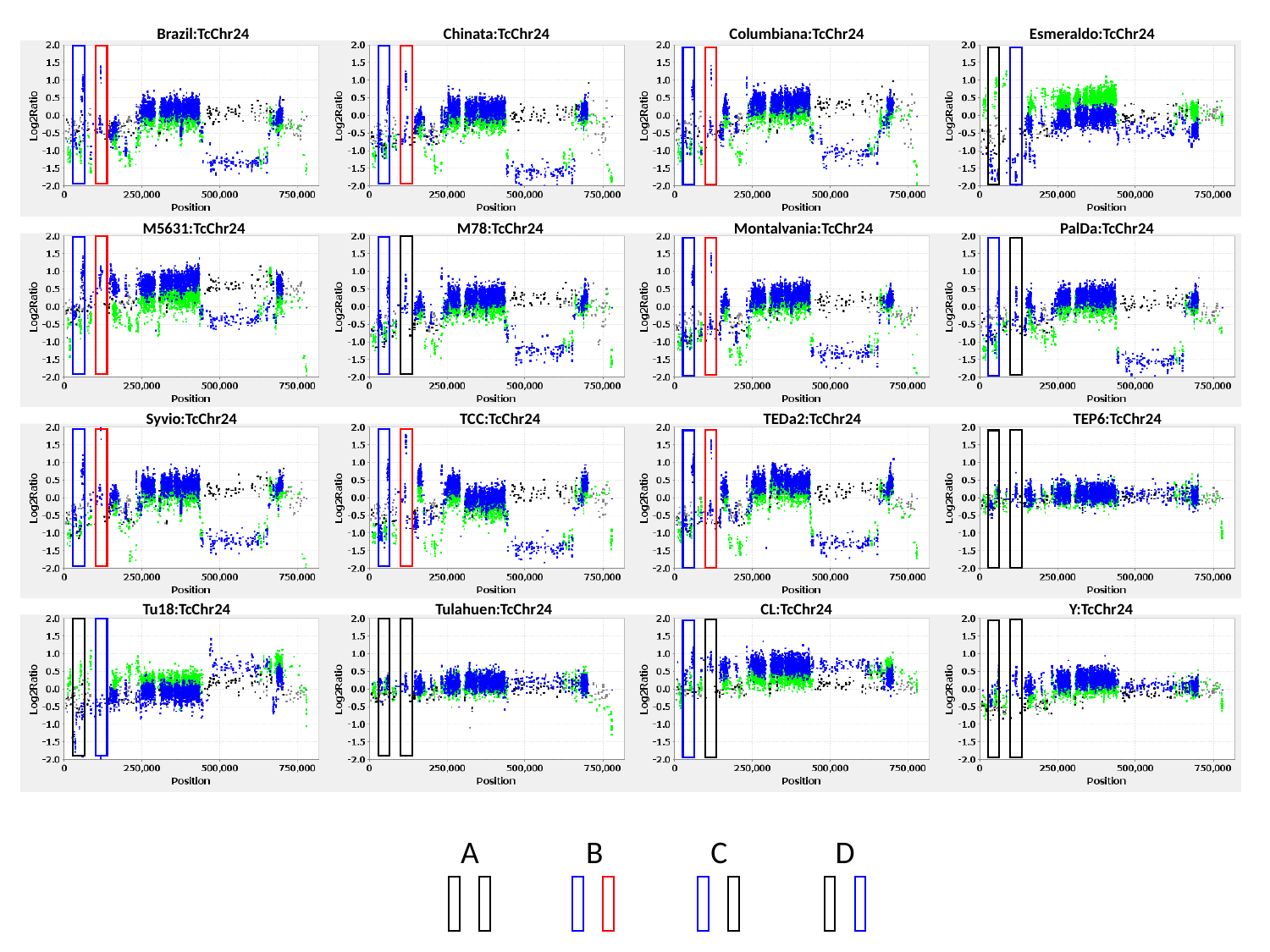

Brazil:TcChr24 Chinata:TcChr24 Columbiana:TcChr24 Esmeraldo:TcChr24
 M5631:TcChr24 M78:TcChr24 Montalvania:TcChr24 PalDa:TcChr24
 Syvio:TcChr24 TCC:TcChr24 TEDa2:TcChr24 TEP6:TcChr24
 Tu18:TcChr24 Tulahuen:TcChr24 CL:TcChr24 Y:TcChr24
A
B
C
D

## Slide 25
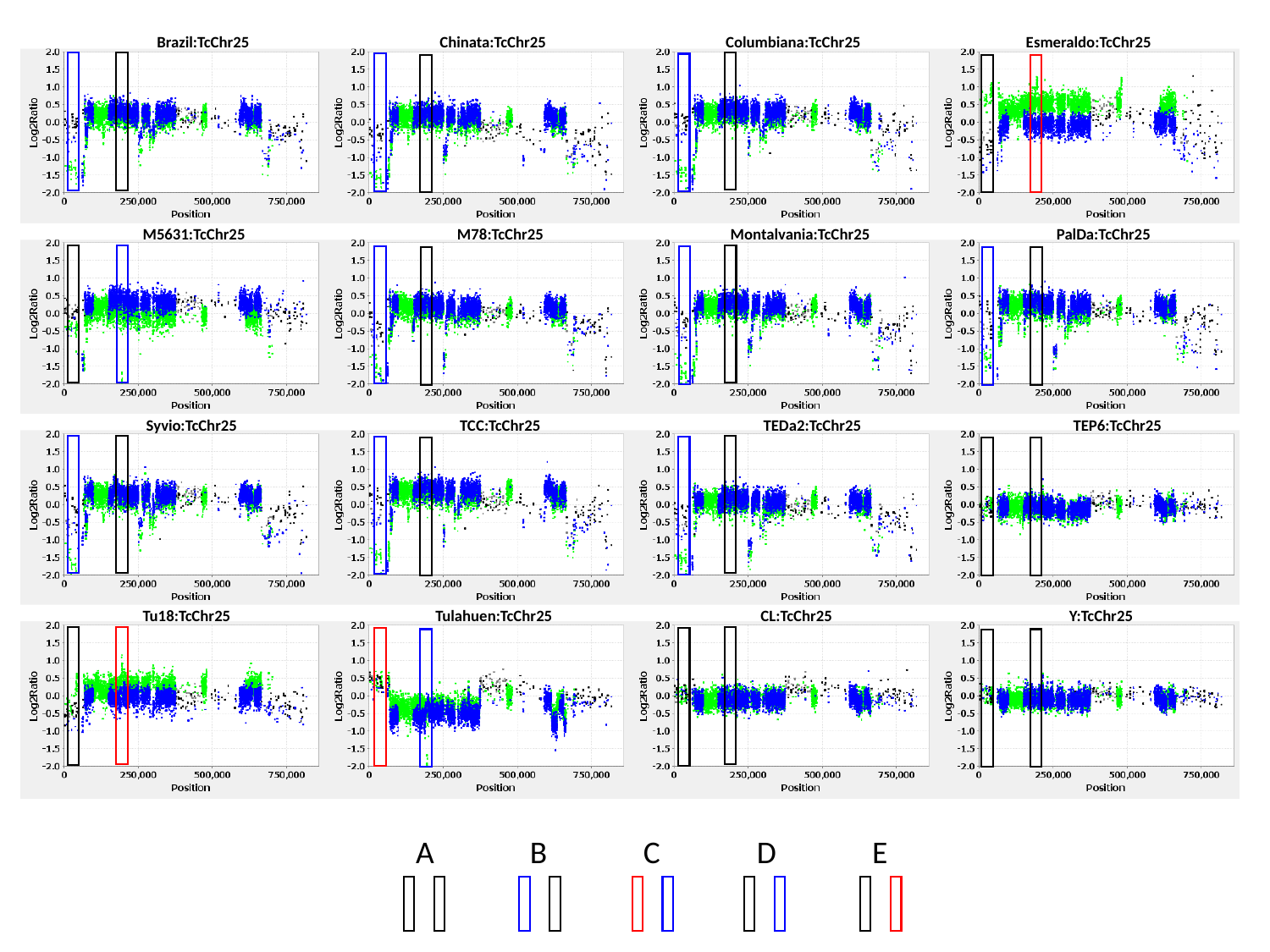

Brazil:TcChr25 Chinata:TcChr25 Columbiana:TcChr25 Esmeraldo:TcChr25
 M5631:TcChr25 M78:TcChr25 Montalvania:TcChr25 PalDa:TcChr25
 Syvio:TcChr25 TCC:TcChr25 TEDa2:TcChr25 TEP6:TcChr25
 Tu18:TcChr25 Tulahuen:TcChr25 CL:TcChr25 Y:TcChr25
A
B
C
D
E

## Slide 26
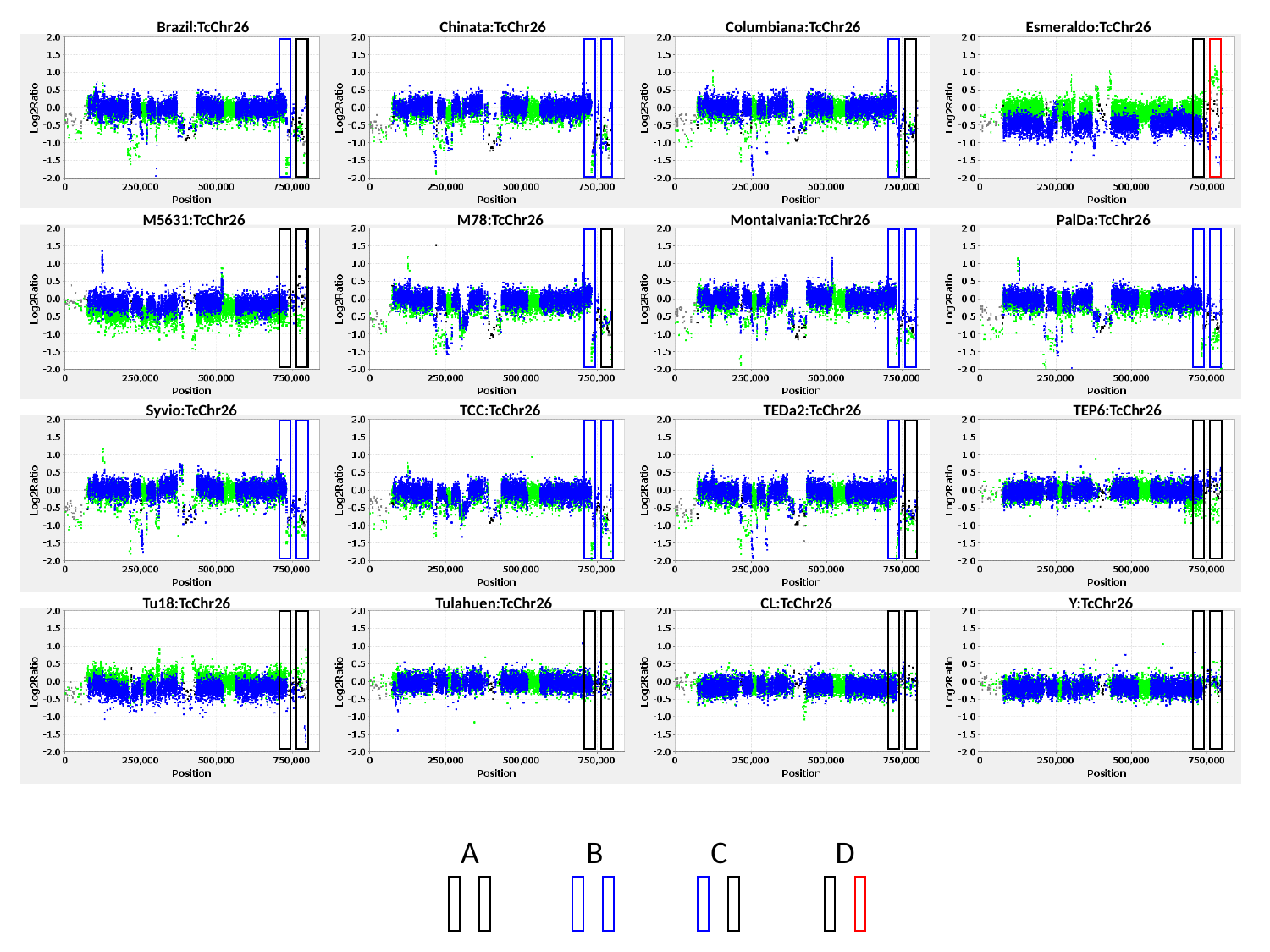

Brazil:TcChr26 Chinata:TcChr26 Columbiana:TcChr26 Esmeraldo:TcChr26
 M5631:TcChr26 M78:TcChr26 Montalvania:TcChr26 PalDa:TcChr26
 Syvio:TcChr26 TCC:TcChr26 TEDa2:TcChr26 TEP6:TcChr26
 Tu18:TcChr26 Tulahuen:TcChr26 CL:TcChr26 Y:TcChr26
A
B
C
D

## Slide 27
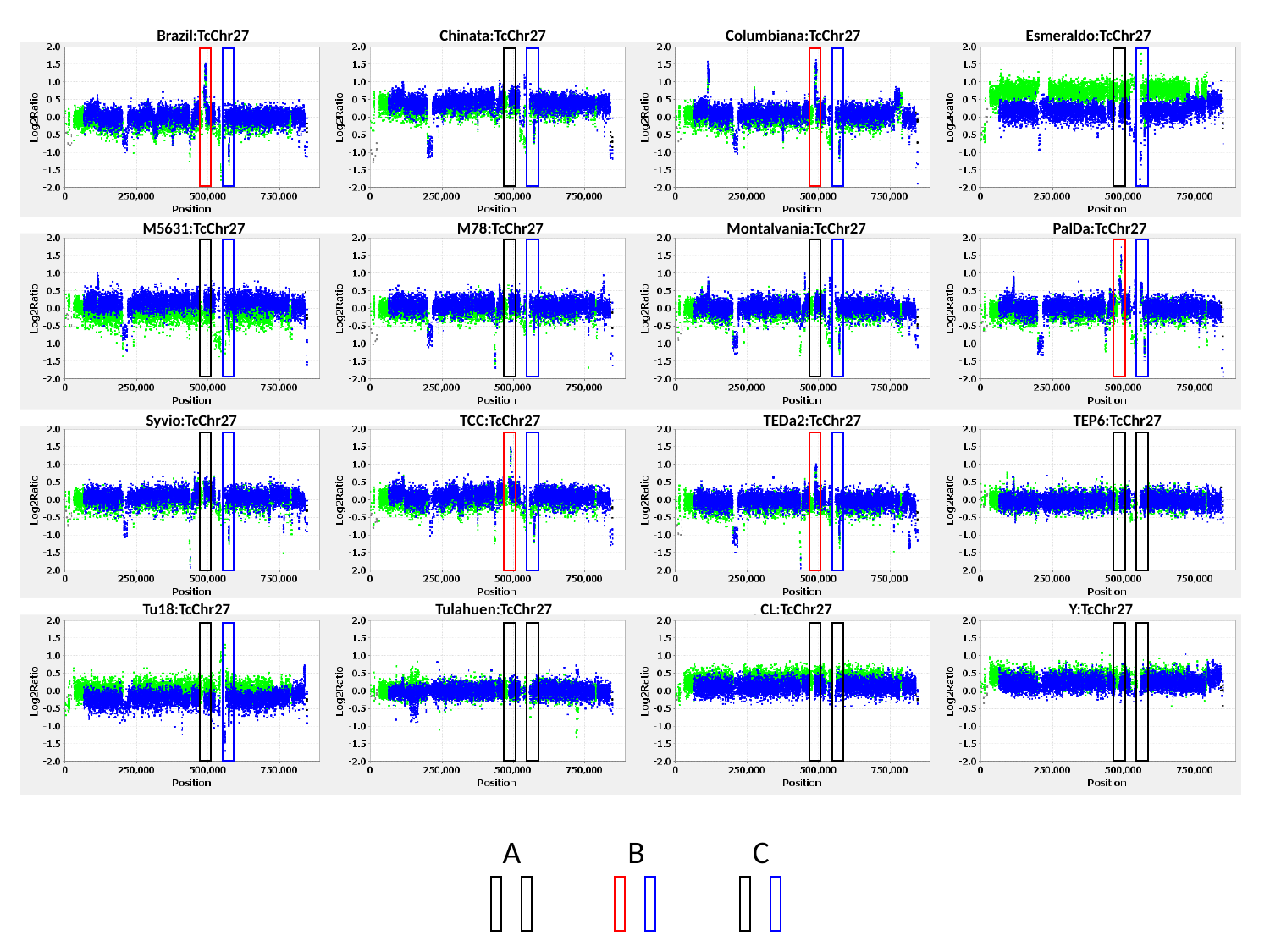

Brazil:TcChr27 Chinata:TcChr27 Columbiana:TcChr27 Esmeraldo:TcChr27
 M5631:TcChr27 M78:TcChr27 Montalvania:TcChr27 PalDa:TcChr27
 Syvio:TcChr27 TCC:TcChr27 TEDa2:TcChr27 TEP6:TcChr27
 Tu18:TcChr27 Tulahuen:TcChr27 CL:TcChr27 Y:TcChr27
A
B
C

## Slide 28
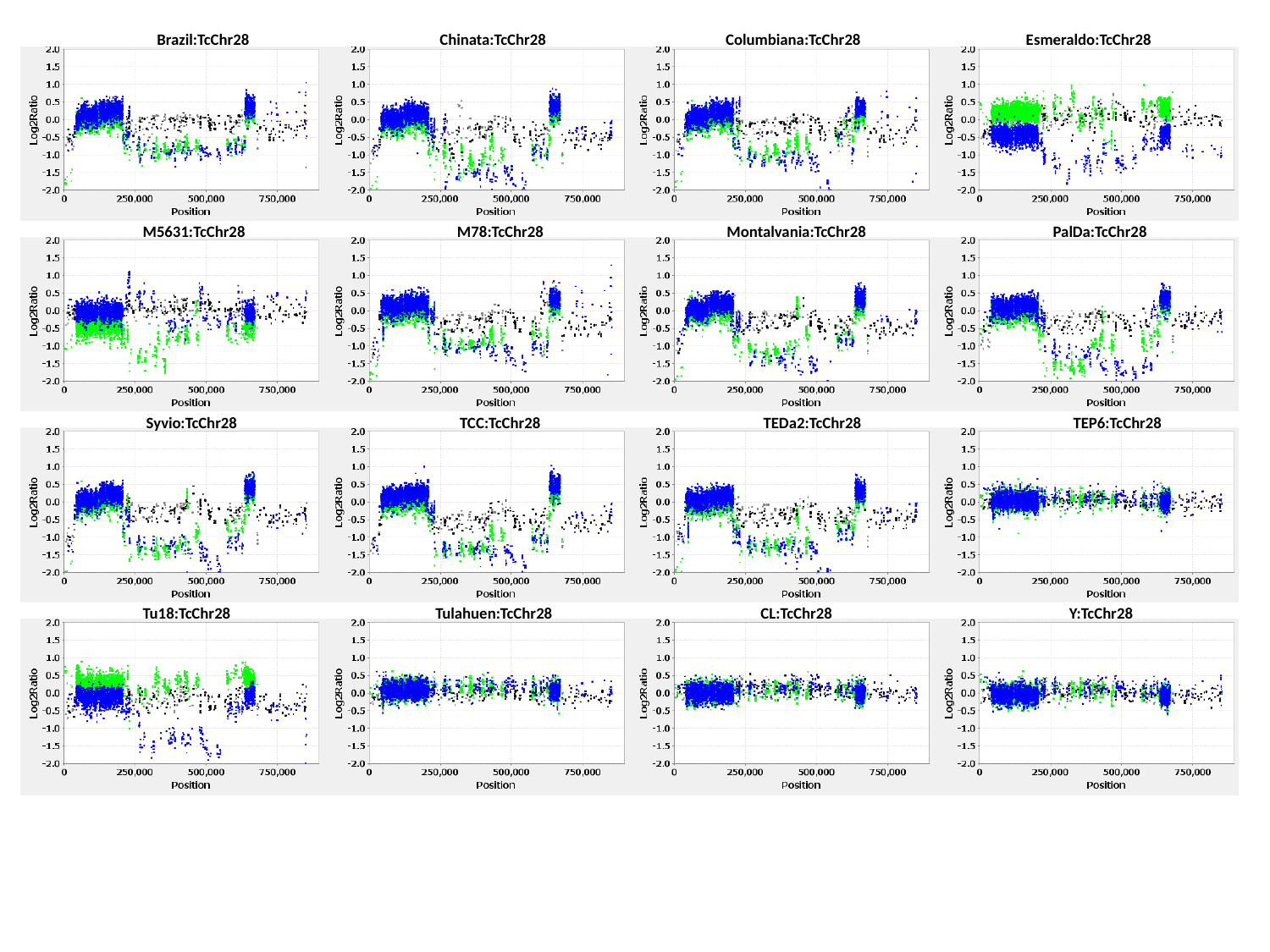

Brazil:TcChr28 Chinata:TcChr28 Columbiana:TcChr28 Esmeraldo:TcChr28
 M5631:TcChr28 M78:TcChr28 Montalvania:TcChr28 PalDa:TcChr28
 Syvio:TcChr28 TCC:TcChr28 TEDa2:TcChr28 TEP6:TcChr28
 Tu18:TcChr28 Tulahuen:TcChr28 CL:TcChr28 Y:TcChr28

## Slide 29
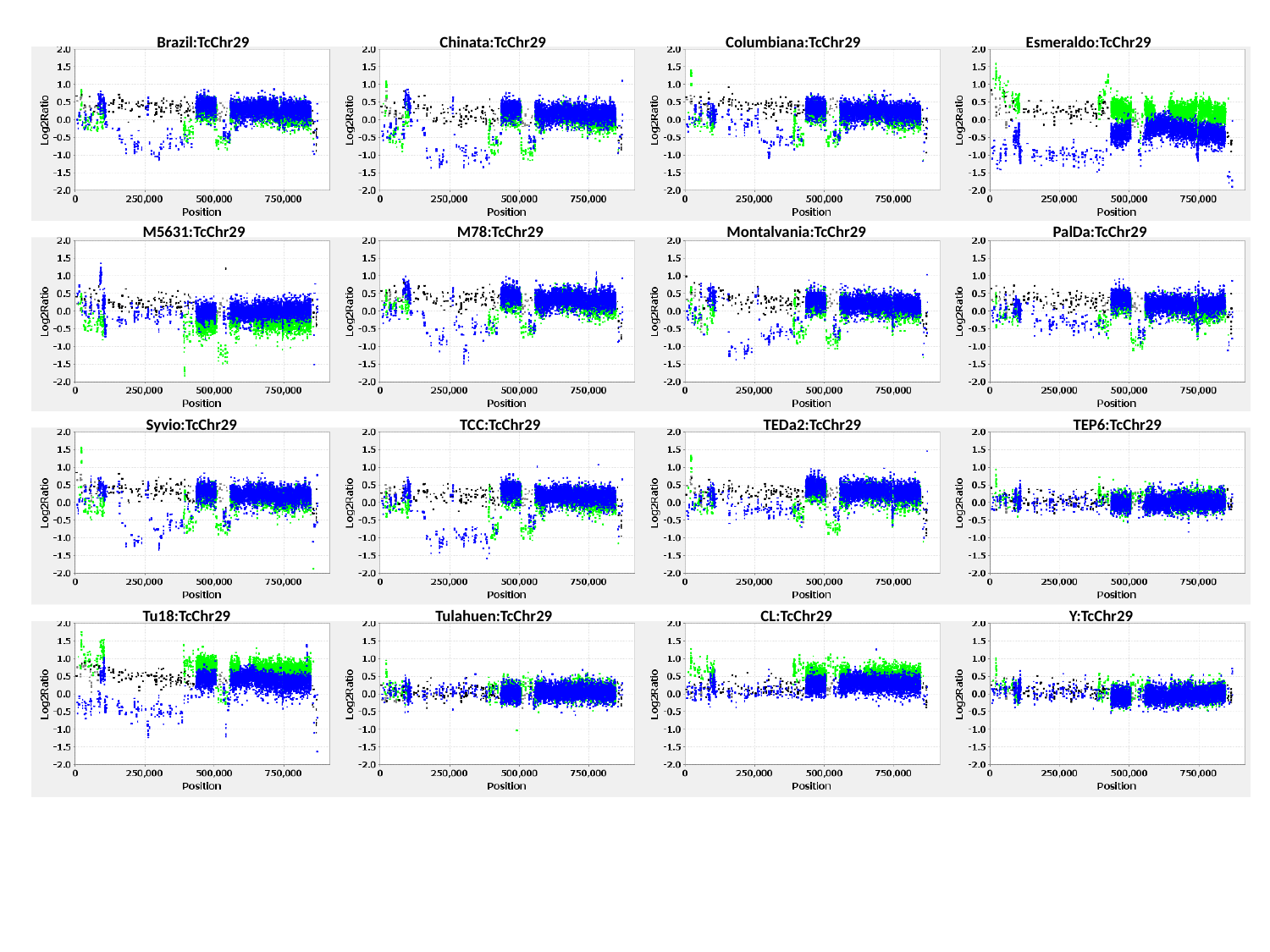

Brazil:TcChr29 Chinata:TcChr29 Columbiana:TcChr29 Esmeraldo:TcChr29
 M5631:TcChr29 M78:TcChr29 Montalvania:TcChr29 PalDa:TcChr29
 Syvio:TcChr29 TCC:TcChr29 TEDa2:TcChr29 TEP6:TcChr29
 Tu18:TcChr29 Tulahuen:TcChr29 CL:TcChr29 Y:TcChr29

## Slide 30
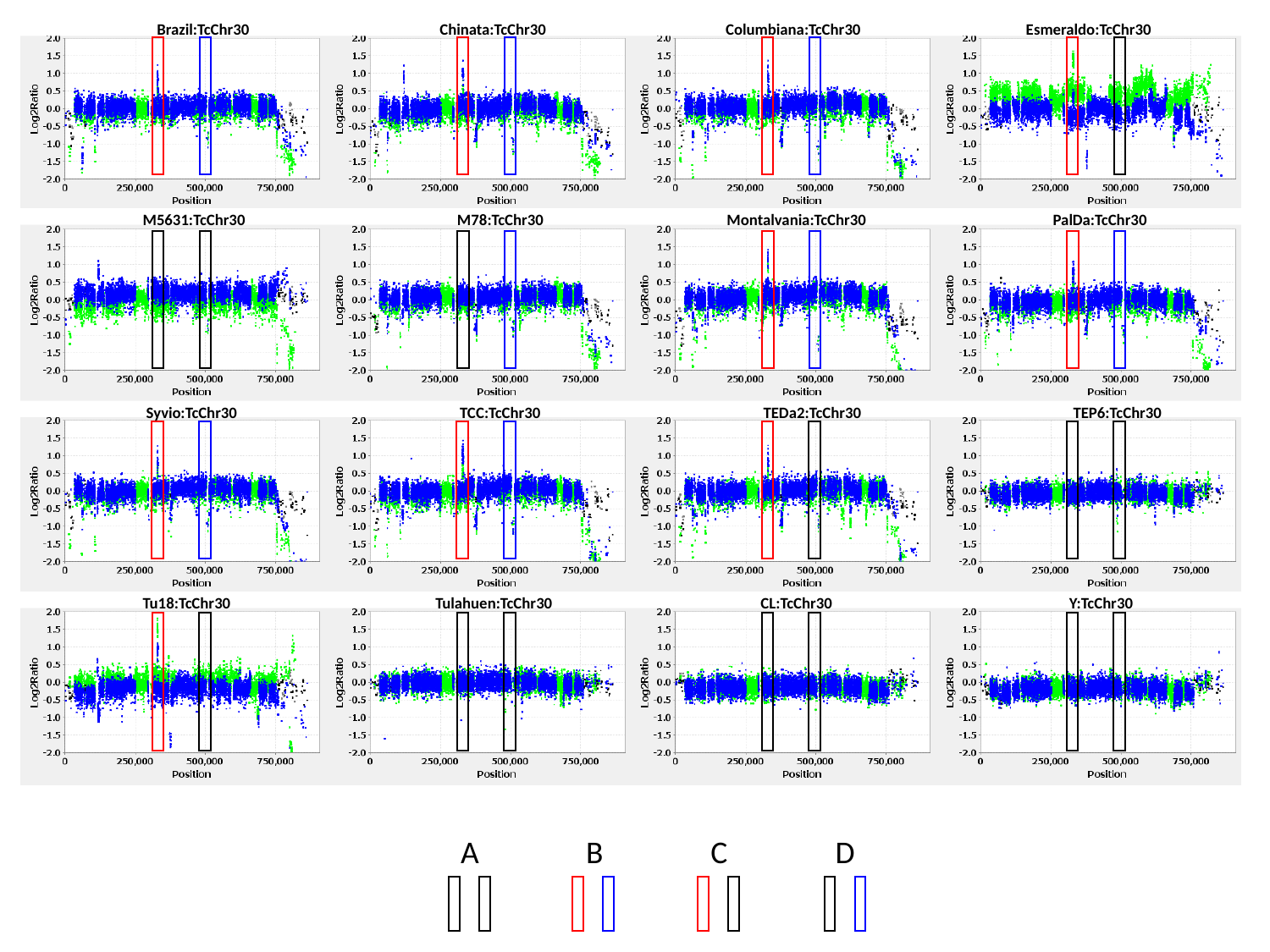

Brazil:TcChr30 Chinata:TcChr30 Columbiana:TcChr30 Esmeraldo:TcChr30
 M5631:TcChr30 M78:TcChr30 Montalvania:TcChr30 PalDa:TcChr30
 Syvio:TcChr30 TCC:TcChr30 TEDa2:TcChr30 TEP6:TcChr30
 Tu18:TcChr30 Tulahuen:TcChr30 CL:TcChr30 Y:TcChr30
A
B
C
D

## Slide 31
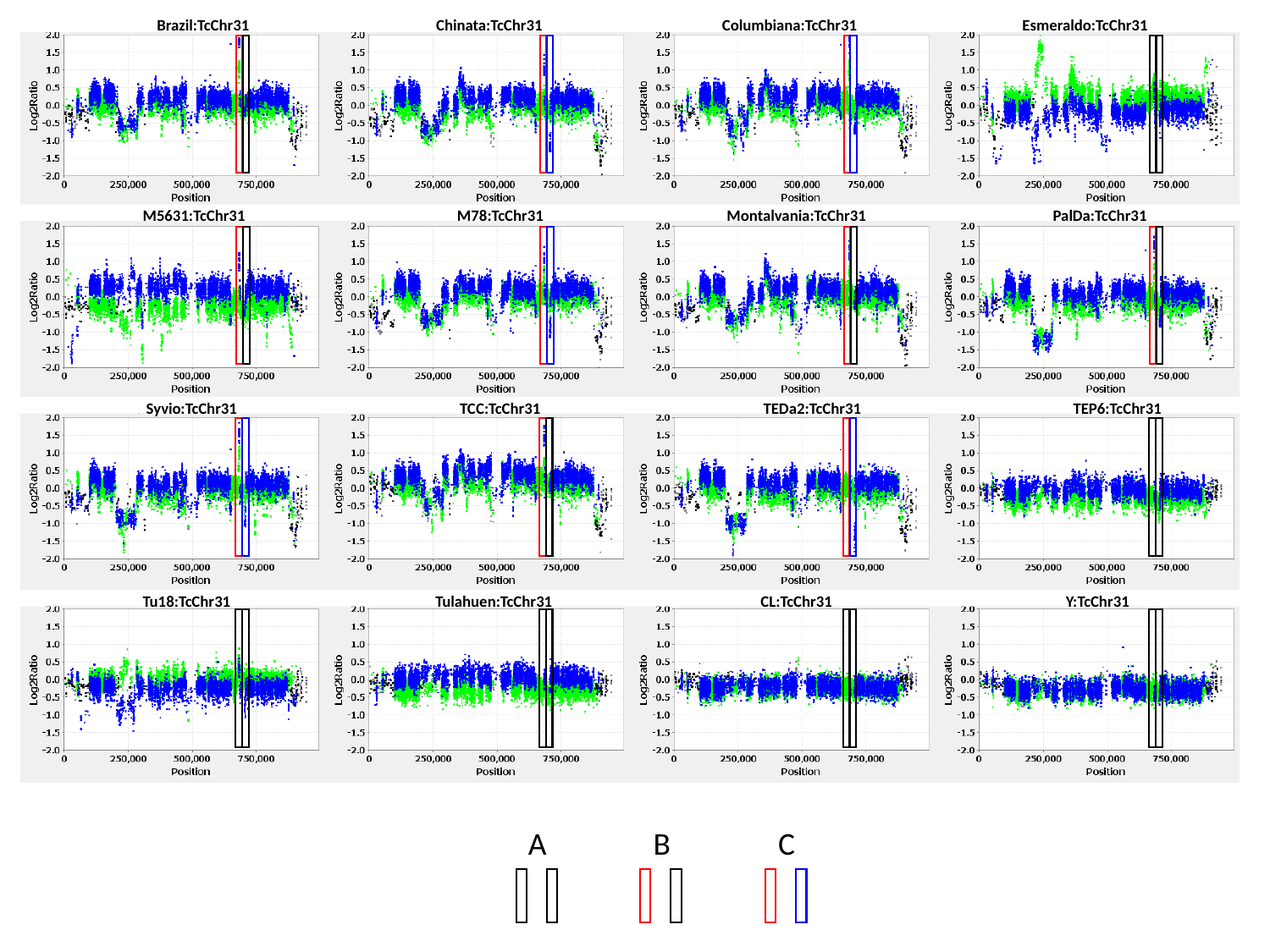

Brazil:TcChr31 Chinata:TcChr31 Columbiana:TcChr31 Esmeraldo:TcChr31
 M5631:TcChr31 M78:TcChr31 Montalvania:TcChr31 PalDa:TcChr31
 Syvio:TcChr31 TCC:TcChr31 TEDa2:TcChr31 TEP6:TcChr31
 Tu18:TcChr31 Tulahuen:TcChr31 CL:TcChr31 Y:TcChr31
A
B
C

## Slide 32
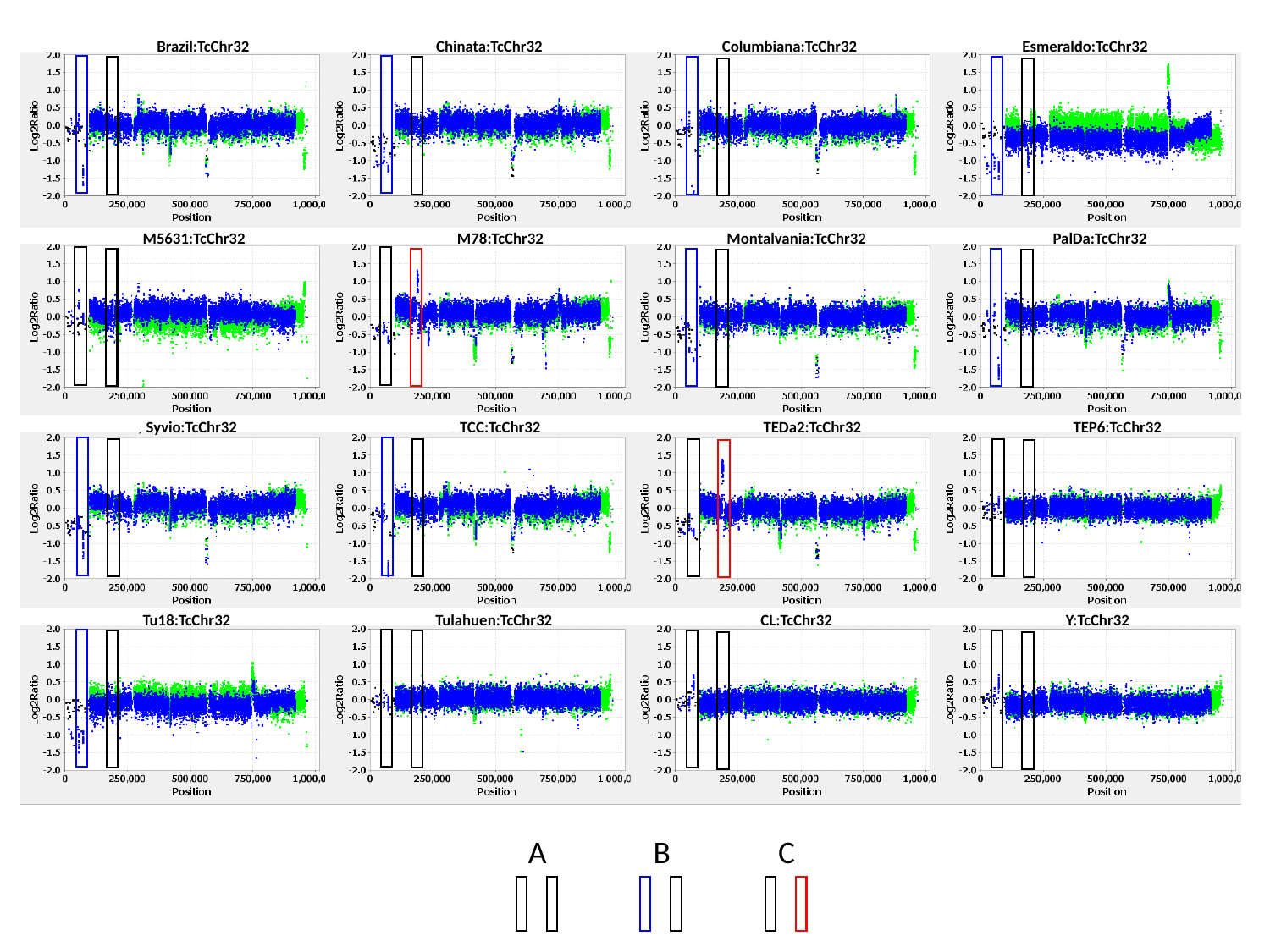

Brazil:TcChr32 Chinata:TcChr32 Columbiana:TcChr32 Esmeraldo:TcChr32
 M5631:TcChr32 M78:TcChr32 Montalvania:TcChr32 PalDa:TcChr32
 Syvio:TcChr32 TCC:TcChr32 TEDa2:TcChr32 TEP6:TcChr32
 Tu18:TcChr32 Tulahuen:TcChr32 CL:TcChr32 Y:TcChr32
A
B
C

## Slide 33
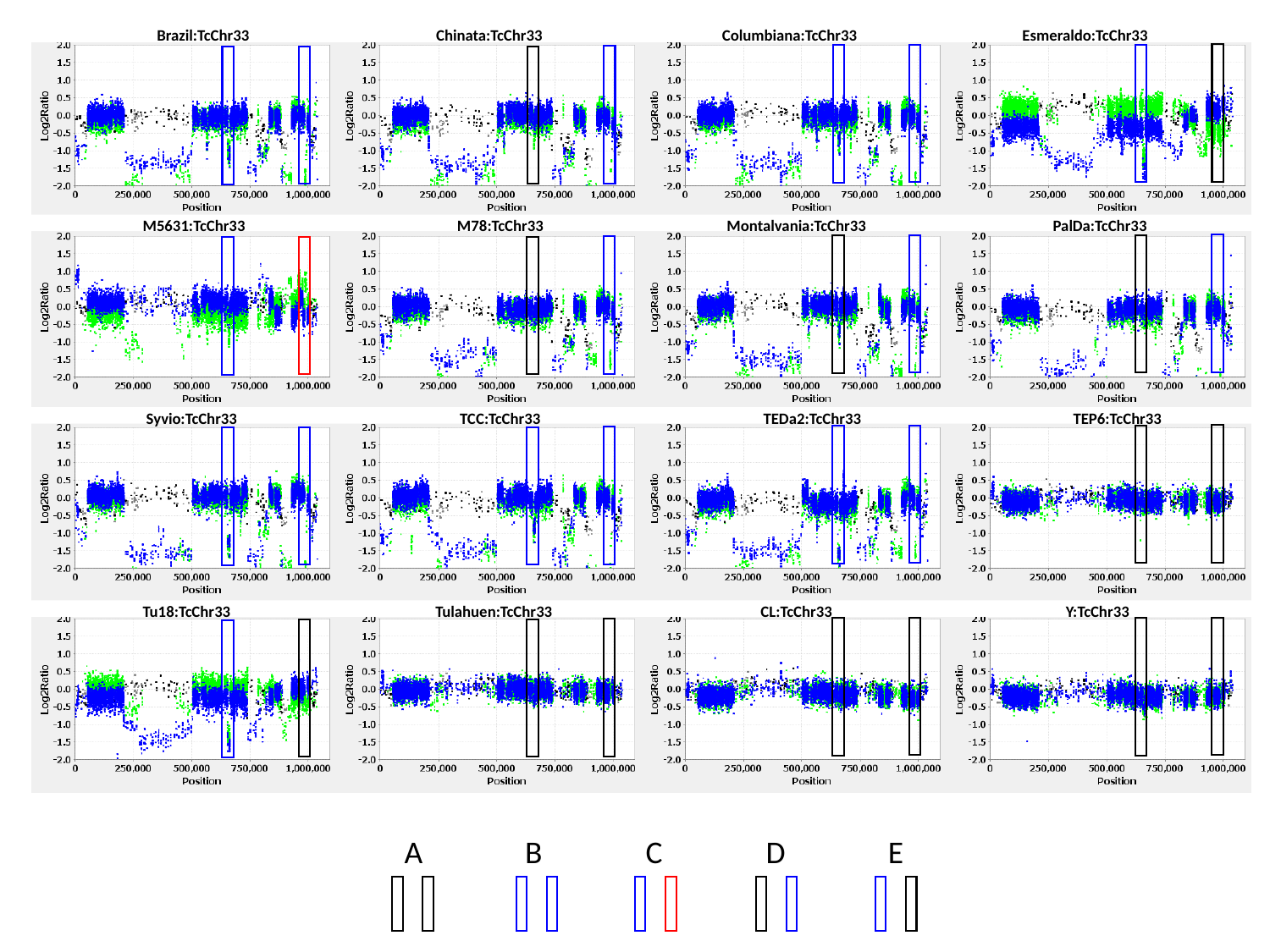

Brazil:TcChr33 Chinata:TcChr33 Columbiana:TcChr33 Esmeraldo:TcChr33
 M5631:TcChr33 M78:TcChr33 Montalvania:TcChr33 PalDa:TcChr33
 Syvio:TcChr33 TCC:TcChr33 TEDa2:TcChr33 TEP6:TcChr33
 Tu18:TcChr33 Tulahuen:TcChr33 CL:TcChr33 Y:TcChr33
A
B
C
D
E

## Slide 34
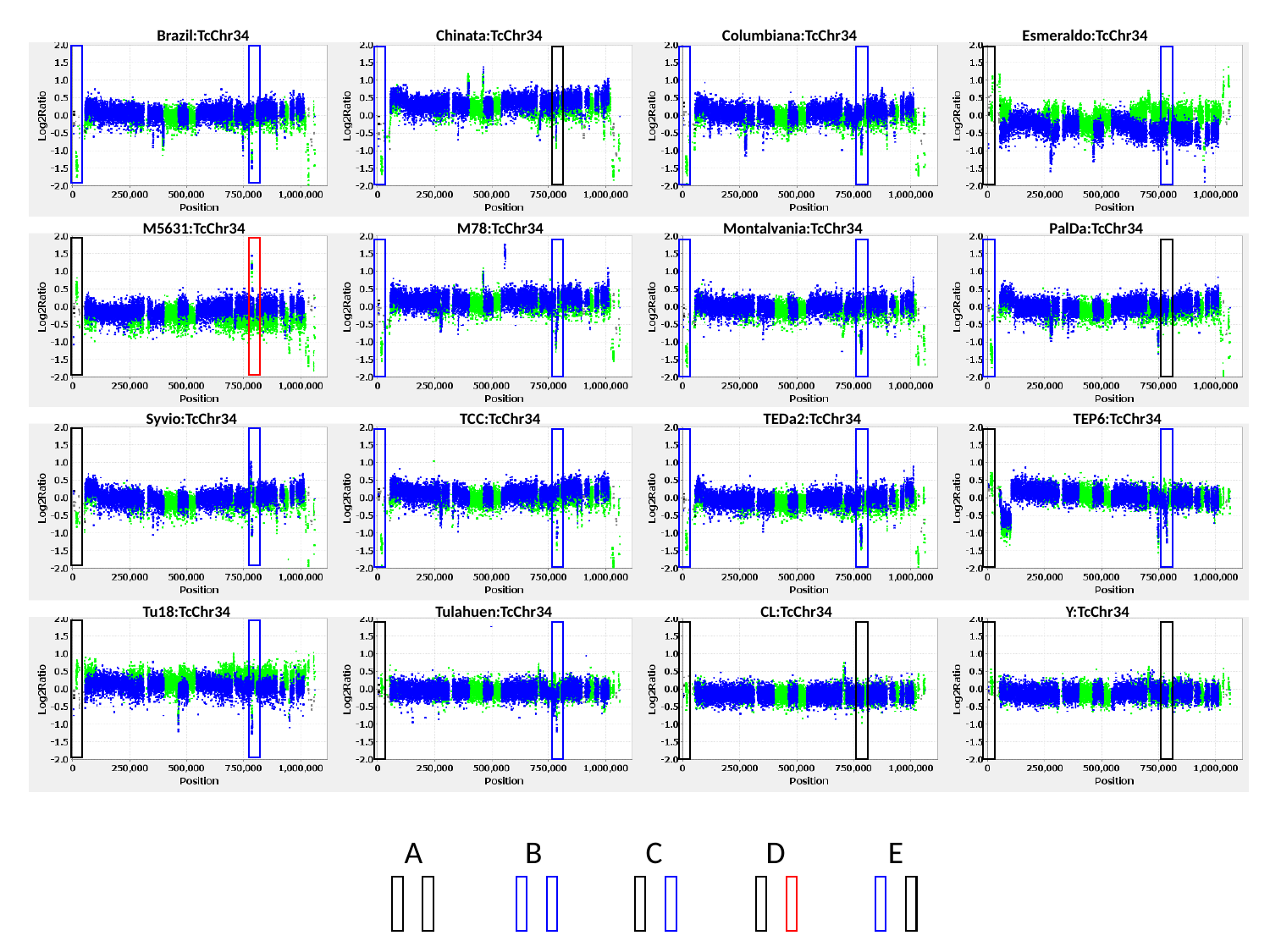

Brazil:TcChr34 Chinata:TcChr34 Columbiana:TcChr34 Esmeraldo:TcChr34
 M5631:TcChr34 M78:TcChr34 Montalvania:TcChr34 PalDa:TcChr34
 Syvio:TcChr34 TCC:TcChr34 TEDa2:TcChr34 TEP6:TcChr34
 Tu18:TcChr34 Tulahuen:TcChr34 CL:TcChr34 Y:TcChr34
A
B
C
D
E

## Slide 35
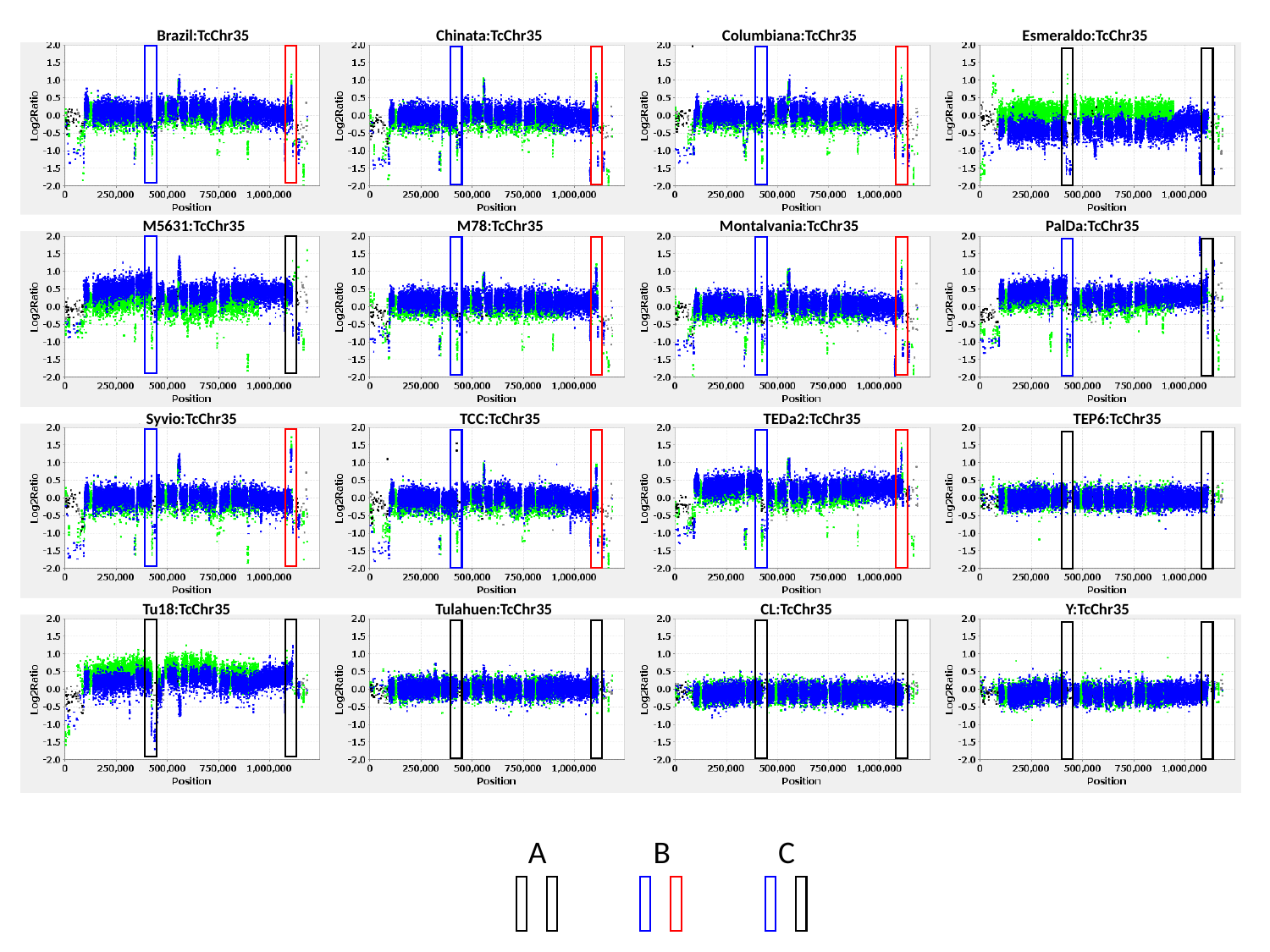

Brazil:TcChr35 Chinata:TcChr35 Columbiana:TcChr35 Esmeraldo:TcChr35
 M5631:TcChr35 M78:TcChr35 Montalvania:TcChr35 PalDa:TcChr35
 Syvio:TcChr35 TCC:TcChr35 TEDa2:TcChr35 TEP6:TcChr35
 Tu18:TcChr35 Tulahuen:TcChr35 CL:TcChr35 Y:TcChr35
A
B
C

## Slide 36
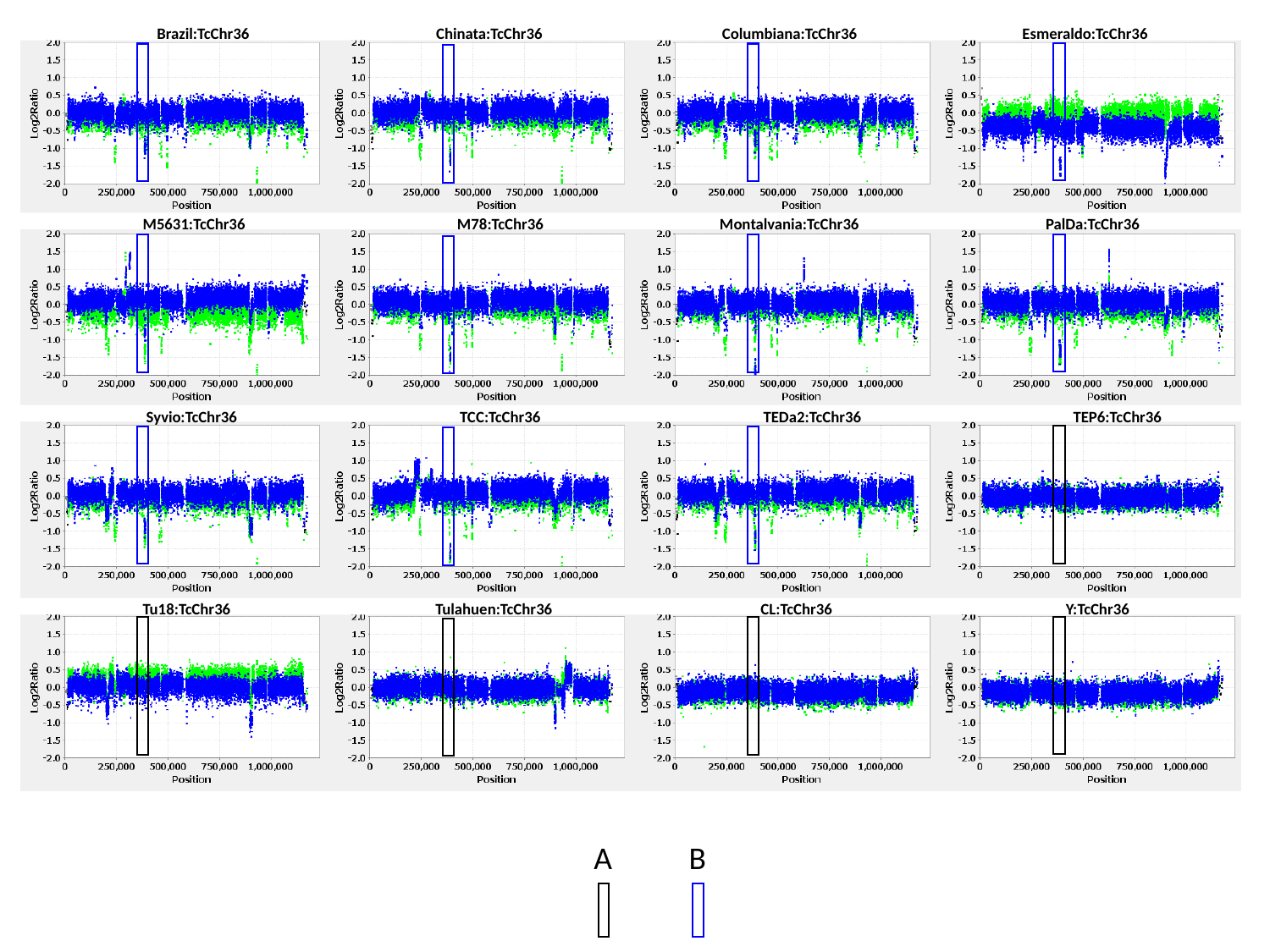

Brazil:TcChr36 Chinata:TcChr36 Columbiana:TcChr36 Esmeraldo:TcChr36
 M5631:TcChr36 M78:TcChr36 Montalvania:TcChr36 PalDa:TcChr36
 Syvio:TcChr36 TCC:TcChr36 TEDa2:TcChr36 TEP6:TcChr36
 Tu18:TcChr36 Tulahuen:TcChr36 CL:TcChr36 Y:TcChr36
A
B

## Slide 37
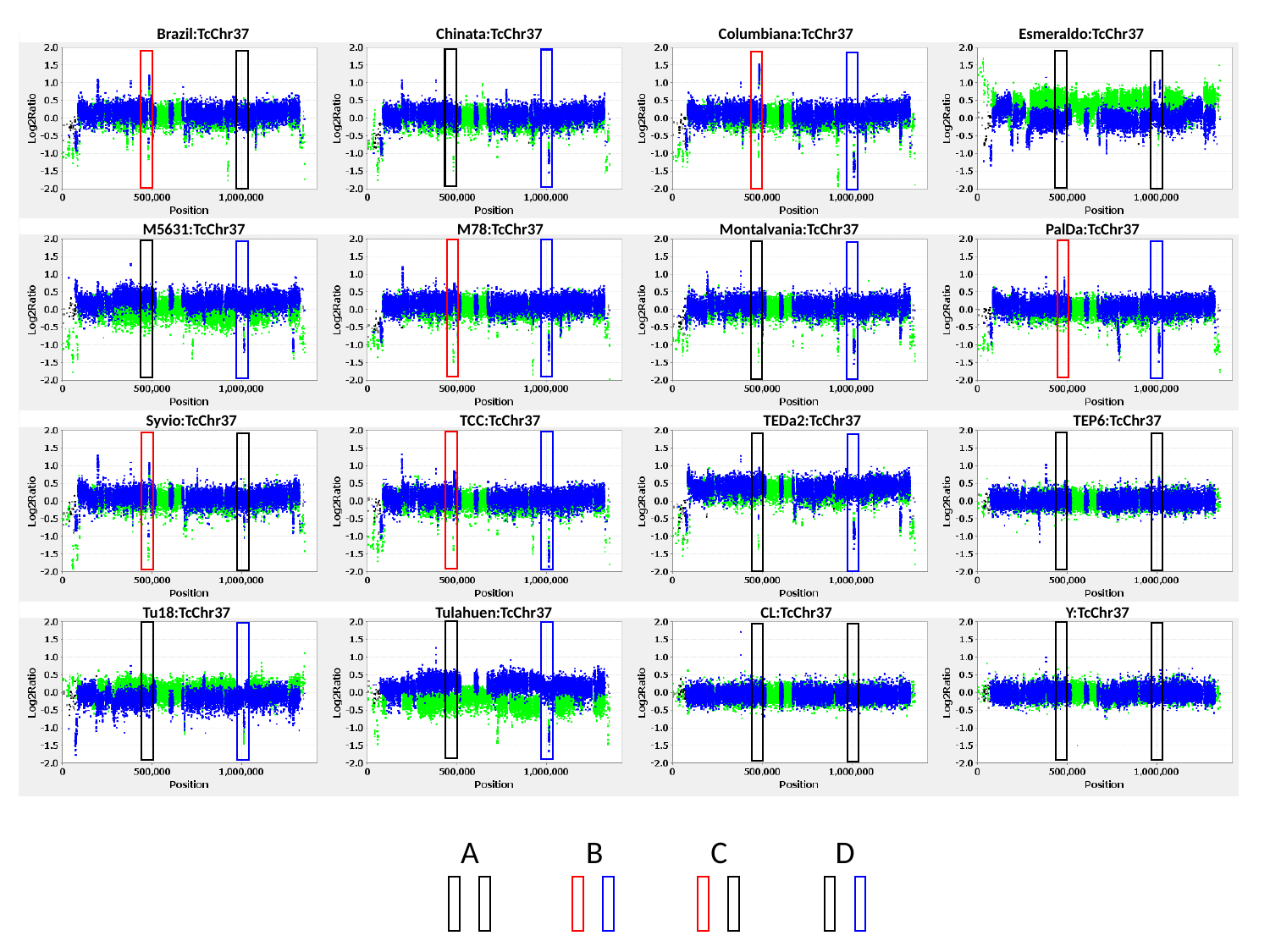

Brazil:TcChr37 Chinata:TcChr37 Columbiana:TcChr37 Esmeraldo:TcChr37
 M5631:TcChr37 M78:TcChr37 Montalvania:TcChr37 PalDa:TcChr37
 Syvio:TcChr37 TCC:TcChr37 TEDa2:TcChr37 TEP6:TcChr37
 Tu18:TcChr37 Tulahuen:TcChr37 CL:TcChr37 Y:TcChr37
A
B
C
D

## Slide 38
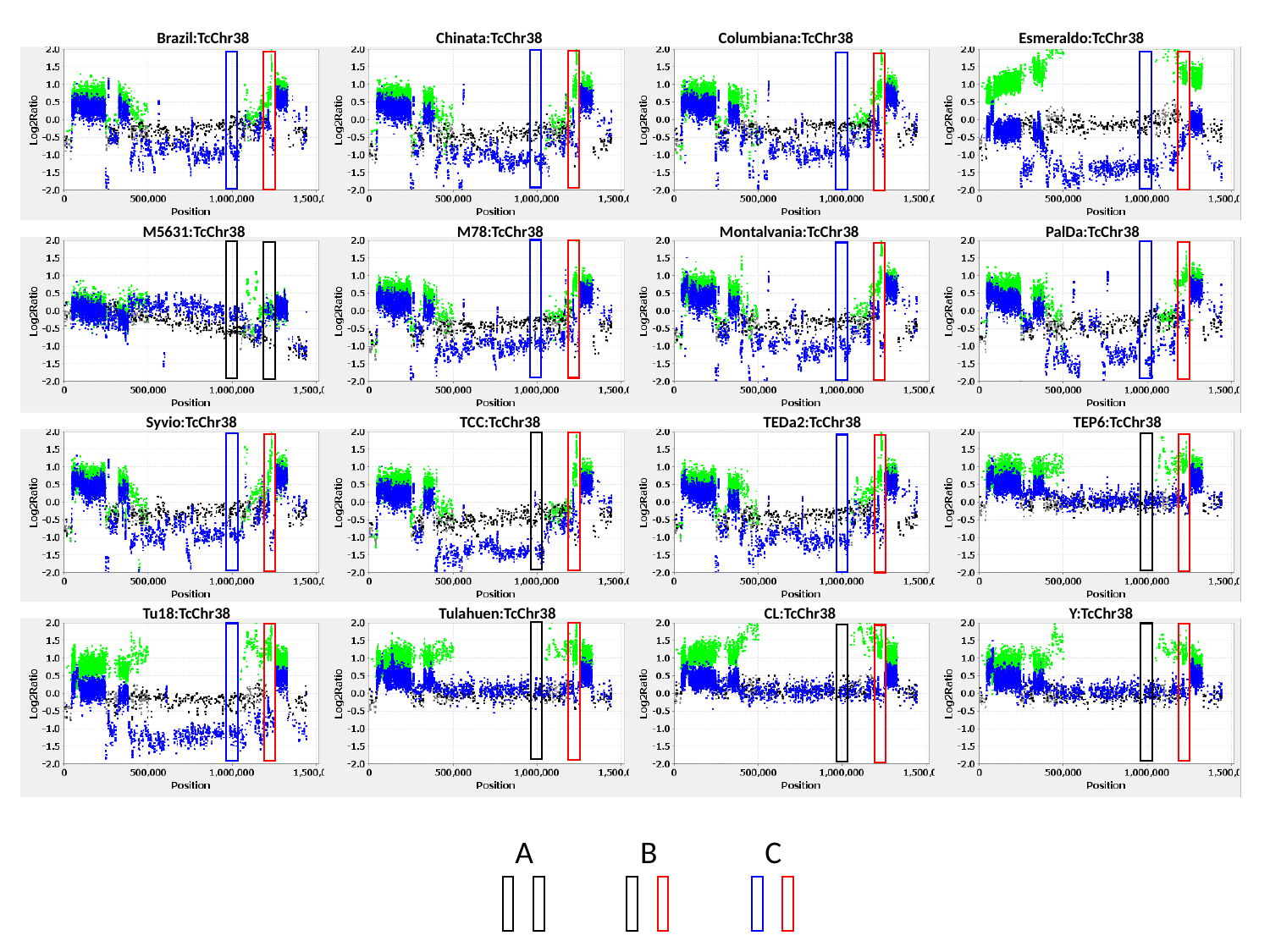

Brazil:TcChr38 Chinata:TcChr38 Columbiana:TcChr38 Esmeraldo:TcChr38
 M5631:TcChr38 M78:TcChr38 Montalvania:TcChr38 PalDa:TcChr38
 Syvio:TcChr38 TCC:TcChr38 TEDa2:TcChr38 TEP6:TcChr38
 Tu18:TcChr38 Tulahuen:TcChr38 CL:TcChr38 Y:TcChr38
A
B
C

## Slide 39
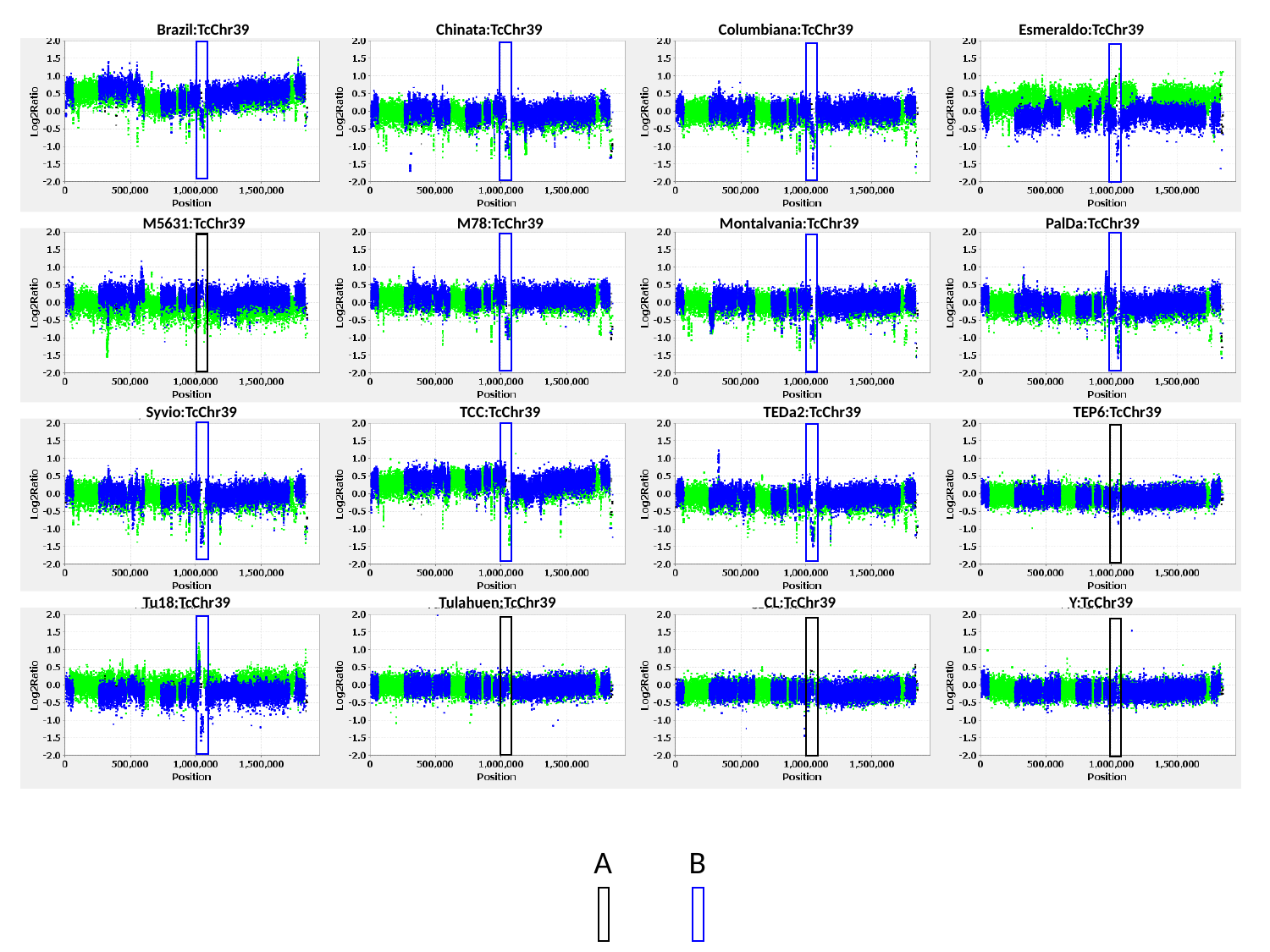

Brazil:TcChr39 Chinata:TcChr39 Columbiana:TcChr39 Esmeraldo:TcChr39
 M5631:TcChr39 M78:TcChr39 Montalvania:TcChr39 PalDa:TcChr39
 Syvio:TcChr39 TCC:TcChr39 TEDa2:TcChr39 TEP6:TcChr39
 Tu18:TcChr39 Tulahuen:TcChr39 CL:TcChr39 Y:TcChr39
A
B

## Slide 40
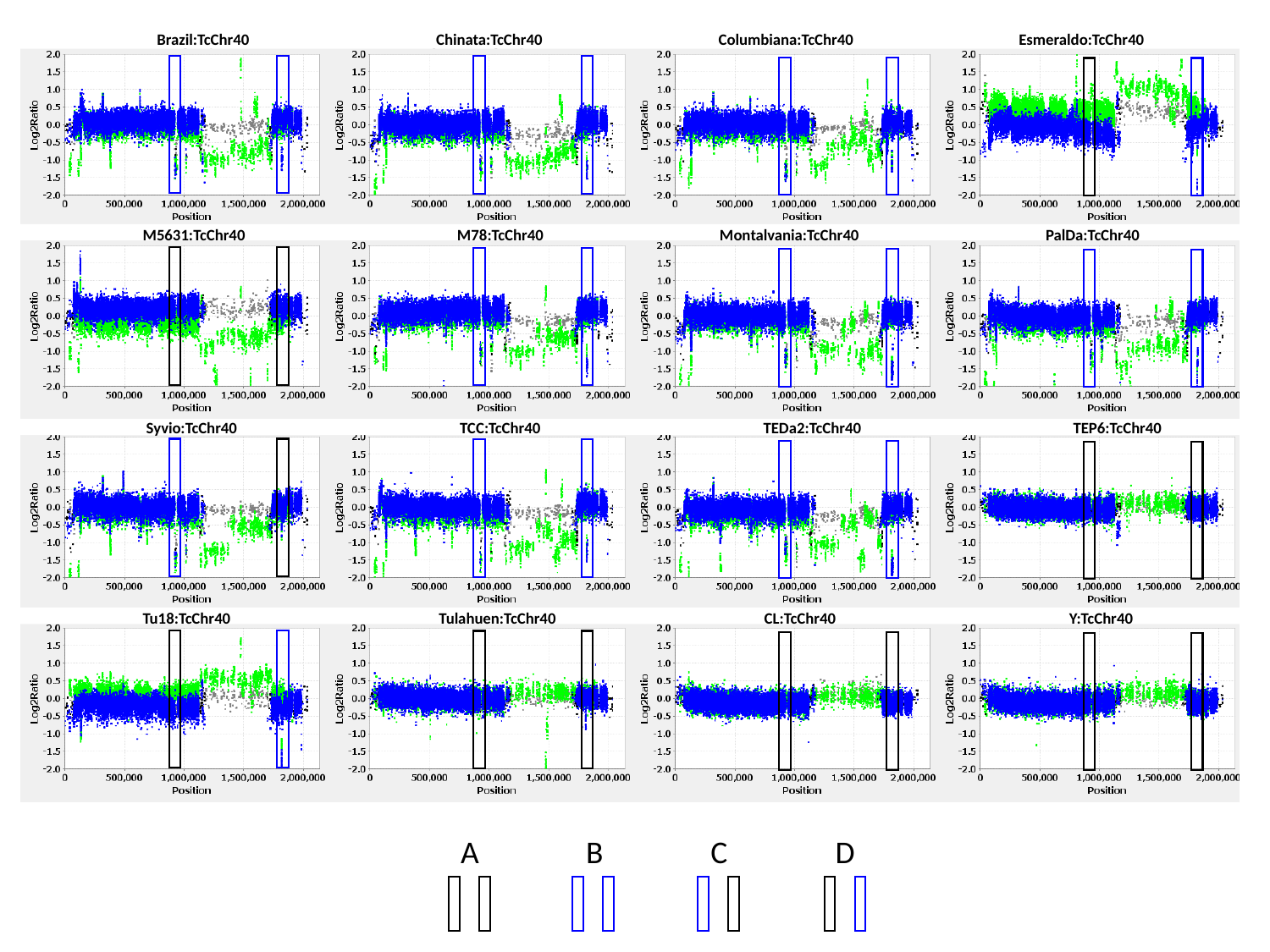

Brazil:TcChr40 Chinata:TcChr40 Columbiana:TcChr40 Esmeraldo:TcChr40
 M5631:TcChr40 M78:TcChr40 Montalvania:TcChr40 PalDa:TcChr40
 Syvio:TcChr40 TCC:TcChr40 TEDa2:TcChr40 TEP6:TcChr40
 Tu18:TcChr40 Tulahuen:TcChr40 CL:TcChr40 Y:TcChr40
A
B
C
D

## Slide 41
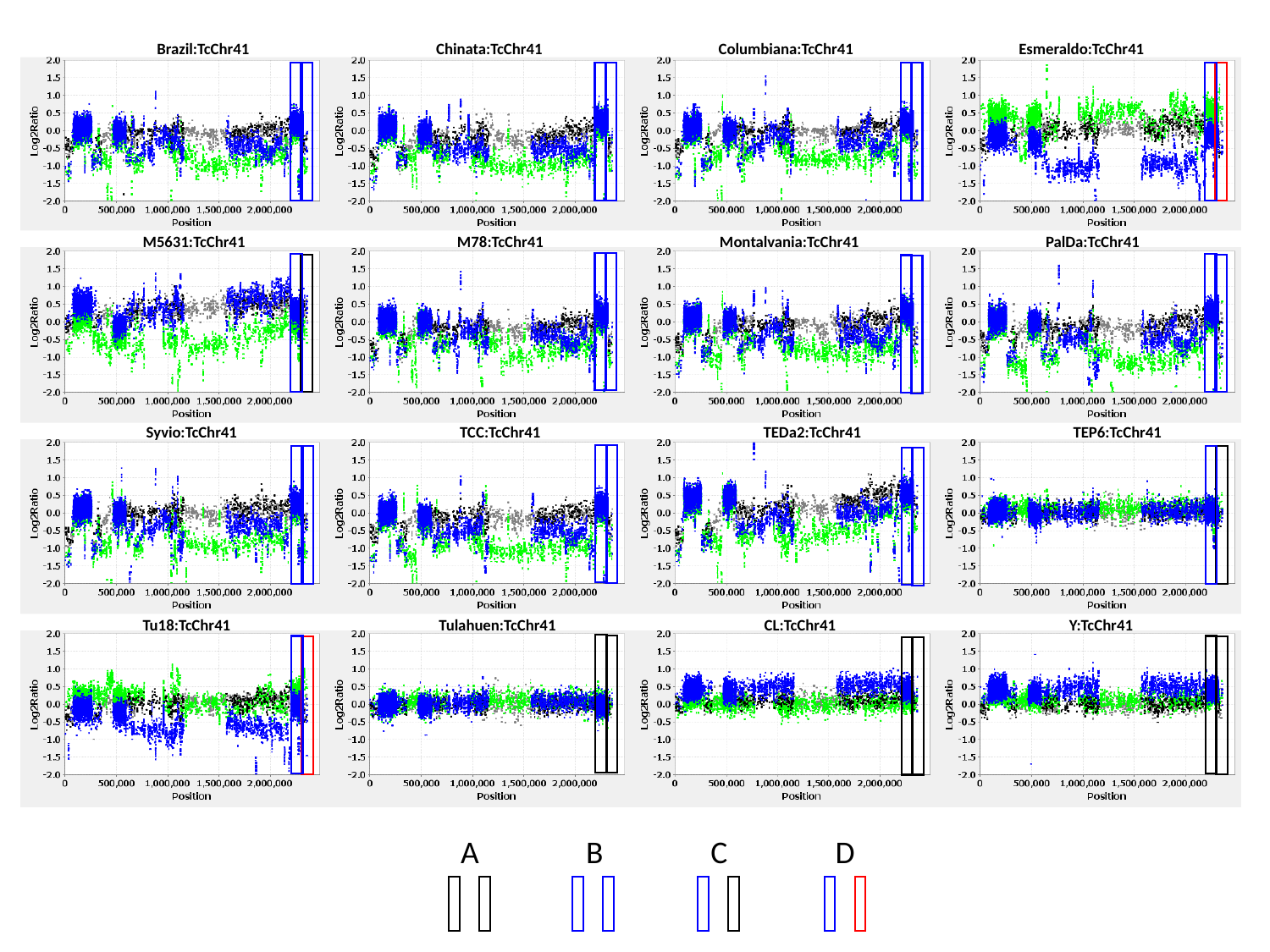

Brazil:TcChr41 Chinata:TcChr41 Columbiana:TcChr41 Esmeraldo:TcChr41
 M5631:TcChr41 M78:TcChr41 Montalvania:TcChr41 PalDa:TcChr41
 Syvio:TcChr41 TCC:TcChr41 TEDa2:TcChr41 TEP6:TcChr41
 Tu18:TcChr41 Tulahuen:TcChr41 CL:TcChr41 Y:TcChr41
A
B
C
D
