# Supplementary material for: Widespread, focal copy number variations (CNV) and whole chromosome aneuploidies in Trypanosoma cruzi strains revealed by array comparative genomic hybridization
Source: BMC Genomics. 2011 Mar 7;12:139. doi: 10.1186/1471-2164-12-139 (PMC3060142; doi:10.1186/1471-2164-12-139)
Supplement: Additional file 5 — MSWord file containing a chart of the distribution of genes associated with hotspot regions having less than 5 candidates on the arrays. Candidates were determined by having at least 5 probes within a maximum sequence size of 500 bp. [file 1471-2164-12-139-S5.DOCX]

Additional File 5. Distribution of genes associated with hotspot regions, less than 5 candidates on the arrays.

| **Gene Name/UTR** | **# Genome Occurrences^1^** | **#Array Occurrences^2^** | **# Array Candidates^3^** | **# Sig CNV^4^** | **% Sig of Candidates** |
| --- | --- | --- | --- | --- | --- |
| NAD(P)-dependent steroid | 1 | 1 | 1 | 1 | 100 |
| myo-inositol-1 phosphatase | 1 | 1 | 1 | 1 | 100 |
| 90 kDa | 28 | 19 | 1 | 1 | 100 |
| D-isomer specific | 4 | 3 | 1 | 1 | 100 |
| metallopeptidase (pseudogene) | 1 | 1 | 1 | 1 | 100 |
| monoglyceride lipase | 1 | 1 | 1 | 1 | 100 |
| mucin TcMUCI | 55 | 21 | 1 | 1 | 100 |
| dehydrogenase (pseudogene) | 1 | 1 | 1 | 1 | 100 |
| thymidylate kinase | 2 | 2 | 2 | 2 | 100 |
| syntaxin binding | 70 | 49 | 2 | 2 | 100 |
| proline racemase | 3 | 3 | 2 | 2 | 100 |
| ribonuclease mar1 | 4 | 4 | 4 | 3 | 75 |
| histone H2A | 18 | 11 | 3 | 2 | 66.6 |
| oligosaccharyl transferase | 3 | 3 | 3 | 2 | 66.6 |
| C-5 sterol | 3 | 3 | 3 | 2 | 66.6 |
| cellulosomal scaffoldin | 2 | 2 | 2 | 1 | 50 |
| haloacid dehalogenase-like | 2 | 2 | 2 | 1 | 50 |
| mucin-like glycoprotein | 28 | 23 | 2 | 1 | 50 |
| arginine kinase | 2 | 2 | 2 | 1 | 50 |
| thioredoxin-like protein | 2 | 2 | 2 | 1 | 50 |
| ATP-dependent protease | 2 | 2 | 2 | 1 | 50 |
| nucleoside phosphatase | 3 | 2 | 2 | 1 | 50 |
| variant-surface-glycoprotein phospholipase | 2 | 2 | 2 | 1 | 50 |
| poly(ADP-ribose) polymerase | 2 | 2 | 2 | 1 | 50 |
| alanine racemase | 2 | 2 | 2 | 1 | 50 |
| isocitrate dehydrogenase | 2 | 2 | 2 | 1 | 50 |
| clathrin assembly | 4 | 4 | 4 | 2 | 50 |
| nucleolar protein | 5 | 4 | 4 | 2 | 50 |
| NUP-1 protein | 3 | 3 | 3 | 1 | 33.3 |
| NADP-dependent alcohol | 4 | 3 | 3 | 1 | 33.3 |
| endosomal integral | 6 | 4 | 3 | 1 | 33.3 |
| GMP synthase | 4 | 4 | 3 | 1 | 33.3 |
| prostaglandin F2alpha | 3 | 3 | 3 | 1 | 33.3 |
| lipase putative | 9 | 9 | 3 | 1 | 33.3 |

1. # genome occurrences = number of instances for the annotation in the genome.
2. # array occurrences = number of annotated genes with probes on the arrays.
3. # array candidates = number of annotated genes represented by probes on the arrays and which had probe density of 5 unique probes per 500 bp. Genes could have insufficient probe density due to repeat regions or they could have too few probes due to length of the gene (minimum of 5 probes).
4. # sig CNV = CNV with a minimum log2 ratio difference of +/- 0.5, for a minimum of 5 probes over a segment size of 500 bp present in at least 5 test strains.
